# Molecular and biological analysis revealed genetic diversity and high virulence strain of *Toxoplasma gondii* in Japan

**DOI:** 10.1371/journal.pone.0227749

**Published:** 2020-02-03

**Authors:** Junpei Fukumoto, Akinori Yamano, Motomichi Matsuzaki, Hisako Kyan, Tatsunori Masatani, Tomohide Matsuo, Toshihiro Matsui, Mami Murakami, Yasuhiro Takashima, Ryuma Matsubara, Michiru Tahara, Takaya Sakura, Fumihiko Takeuchi, Kisaburo Nagamune

**Affiliations:** 1 Department of Parasitology, National Institute of Infectious Diseases, Shinjyuku-ku, Tokyo, Japan; 2 Graduate School of Life and Environmental Sciences, University of Tsukuba, Tsukuba, Ibaraki, Japan; 3 RIKEN Center for Advanced Intelligence Project, Chuo-ku, Tokyo, Japan; 4 Okinawa Prefectural Institute of Health and Environment, Uruma, Okinawa, Japan; 5 Transboundary Animal Diseases Research Center, Joint Faculty of Veterinary Medicine, Kagoshima University, Korimoto, Kagoshima, Japan; 6 Laboratory of Parasitology, Joint Faculty of Veterinary Medicine, Kagoshima University, Korimoto, Kagoshima, Japan; 7 Graduate School of Applied Biological Sciences and Faculty of Applied Biological Sciences, University of Gifu, Gifu, Gifu, Japan; 8 Department of Gene Diagnostics and Therapeutics, Research Institute National Center for Global Health and Medicine, Shinjyuku-ku, Tokyo, Japan; 9 Faculty of Life and Environmental Sciences, University of Tsukuba, Tsukuba, Ibaraki, Japan; NIH, UNITED STATES

## Abstract

*Toxoplasma gondii* is classified into 16 haplogroups based on a worldwide genotyping study of the parasite. However, only a few isolates from Japan were included in this analysis. To conduct more precise genotyping of *T*. *gondii*, we examined the genotypes of Japanese isolates in this study. DNA sequences of 6 loci were determined in 17 Japanese isolates and compared with those of strains of 16 haplogroups. As a result, Japanese isolates were classified into four groups. We investigated the virulence of some Japanese isolates and found a highly virulent strain in mice, comparable to that of RH strain, although this Japanese isolate was sister to strains of haplogroup 2, which show moderate virulence in mice. We further investigated whether this high virulence isolate had different virulence mechanism and strategy to adapt to Japanese host from other strains by comparing the virulence-related genes, ROP5, 18 and the immunomodulatory gene, ROP16 of the isolate with those of archetypical strains (GT1, ME49 and VEG). This analysis indicated the high virulence of the isolate in mice was partly explained by gene sequences of ROP5 and ROP16. These findings lead to the elucidation of biodiversity of *T*. *gondii* and have potential to optimize the diagnostic protocol.

## Introduction

*Toxoplasma gondii* is a parasitic protist and a member of the phylum Apicomplexa. Felines serve as definitive hosts, and other mammals, including humans, and birds are intermediate hosts [1]. Nearly one-third of the world’s population is infected with *T*. *gondii* [2]. Intake of oocysts or tissue cysts is a major source of the infection. *T*. *gondii* infection is latent in most hosts, but individuals who are immunosuppressed due to acquired immunodeficiency syndrome (AIDS) or organ transplantation may develop lethal diseases, including encephalitis [3]. In a pregnant woman with initial *T*. *gondii* infection, a fetus may be infected through vertical transmission, resulting in stillbirth, miscarriage and serious symptoms such as retinochoroiditis, hydrocephalus and psychomotor retardation [3]. Since *T*. *gondii* also causes abortion in livestock [4,5], it is a pathogen of medical and veterinary importance.

In the early 1990s, *T*. *gondii* was thought to be genetically clonal in comparison with other protozoan pathogens. For example, in 1992, single nucleotide polymorphisms (SNPs) in *T*. *gondii* were examined, including in SAG1 and ROP1 loci, using restriction fragment length polymorphism (RFLP). This analysis led to classification of *T*. *gondii* into two clones with different virulence in mice [6]. Following this study, RFLP analysis of 6 loci in 106 strains isolated in Europe and North America led to reclassification of *T*. *gondii* into 3 clones (types I, II and III) [7]. This reclassification has been confirmed using microsatellite markers, gene open reading frames, intron sequences and RFLP research [8]. "3-clone hypothesis" was corresponded with the result of analysis for strains isolated in Europe and North America and had been widely believed for the last decades. However, clones distinct from types I-III have been recently found in South America [9–11] and isolated from wild animals in North America [12]. In these analyses, many clones with RFLP patterns not corresponding to types I-III have been found, but the recent RFLP analysis explained mutations in atypical clones are due to frequent recombination between them [13,14].

Types I-III show different virulence in mammals [6]. Type I is a highly virulent strain and exhibits a 100% lethal dose (LD_100_) in mouse upon injection of 1 parasite. Type II is a low virulence strain (LD_50_ >10^3^), and type III is avirulent (LD_50_ >10^5^) and isolated mainly from livestock. Quantitative trait locus (QTL) analysis revealed major virulence factors of *T*. *gondii* by crossing of high and low virulent clones. ROP18 was first identified as a virulence factor by analysis of crosses between types I and II and types I and III [15, 16]. ROP18 targets the host endoplasmic reticulum-bound transcription factor ATF6β [17] and phosphorylates immunity-related GTPases (IRGs) to escape host clearance [18]. ROP5 has no inherent kinase activity, but works together with ROP18 to phosphorylate IRGs [19]. There is a high copy number of genes encoding ROP5, which is in a tandem repeat in the *T*. *gondii* genome [20,21]. QTL analysis also identified ROP16, which modulates host immune response through the phosphorylation of STAT3 and STAT6 [22], and ROP16 leucine 503 is indispensable for activation of STAT3 [23].

*T*. *gondii* was reclassified into 6 clades and 16 haplogroups using genome-wide SNPs in 62 strains [13,24]. These analyses mainly used strains from Europe, North and South America, and Africa, with insufficient isolates from Asia and Oceania to allow characterization. Therefore, to complete worldwide genotyping of *T*. *gondii*, further studies are needed using isolates from Asia, in which there are few molecular epidemiological studies and little information on haplogroups. *T*. *gondii* isolated in Okinawa prefecture, the southernmost part of Japan, have been classified by RFLP analysis using one gene (GRA6), based on the 3-clone hypothesis [25,26]. In this analysis, about half of isolates from pigs (46.9%) and goats (42.9%) in Okinawa were classified into haplogroup 1, and almost all of these strains exhibited extreme virulence in mice. However, this result needs to be reexamined because it was based on the conventional 3-clone hypothesis, and new haplogroups were not considered. Given that haplogroup 3, which consists of avirulent strains, is mainly isolated from livestock in Europe and North America, the finding that many pigs and goats in Okinawa may be infected with haplogroup 1 poses a critical problem for public health.

In this study, we conducted phylogenetic analyses and examined phenotypes related with virulence in Japanese isolates of *T*. *gondii*. We also investigated whether known genes responsible for virulence and immunology determine the characteristic of Japanese isolates. This detailed typing and evaluation of virulence are epidemiologically important and helpful for development of vaccination, drug therapy and diagnostic protocols.

## Materials and methods

### Parasite and culture

RH (ATCC50838), ME49 (ATCC50840) and TgCatJpOk1, TgCatJpOk2, TgCatJpOk3 and TgCatJpOk4 (isolates from Japan) were used in the assay. The freeze stocks of TgCatJpOk1-4, TgCatJpGi1/TaJ and TgCatJpTy1/k-3 were provided by Hisako Kyan (Okinawa Prefectural Institute of Health and Environment), Tomohide Matsuo (Transboundary Animal Diseases Research Center, Joint Faculty of Veterinary Medicine, Kagoshima University) and Yasuhiro Takashima (Graduate School of Applied Biological Sciences and Faculty of Applied Biological Sciences, University of Gifu). The parasites were maintained in human foreskin fibroblasts (HFFs) cultured in Dulbecco’s modified Eagle’s medium (DMEM; Wako, Osaka, Japan) supplemented with 10% fetal bovine serum (FBS; Bovogen Biologicals, East Keilor, VIC, Australia), 2 mM L-glutamine (Sigma-Aldrich, St. Louis, MO, USA), 10 mM Hepes buffer (Sigma-Aldrich) and 10 μg/mL gentamicin (Sigma-Aldrich), and serially passaged at 37°C under 5% CO_2_. TgCatJpOk1-4 were used in experiments within 6 month culture.

### Transfer of isolates from in vivo to in vitro

TgCatJpOk1-4 were intraperitoneally inoculated into CD-1 mice (8 weeks old, female, Charles River Laboratories Japan, Yokohama, Japan). After death or survival for one month, brain and lungs were removed and homogenized separately in 1 mM D-PBS (1 ml) (Wako). After passing the lung suspension through a 21G injection needle (Terumo Corp., Tokyo, Japan) twice, the suspension was filtered with a cell strainer of 100-μm pore size (Becton Dickinson, Franklin Lakes, NJ, USA) and a filter unit of 5-μm pore size (Merck Millipore, Darmstadt, Germany) to purify the parasite. After centrifugation at 400×g for 10 min at room temperature, the supernatant was removed and the rest of the suspension was washed with Hanks’ Balanced Salt Solution (Sigma-Aldrich) supplemented with 5 mM Hepes Buffer and 5 mM EGTA (Sigma-Aldrich). After another wash, the rest of the suspension was mixed with culture medium (5 ml) and inoculated in HFFs. The brain suspension (350 μl) was treated in the same manner as the lung suspension and inoculated in HFFs. The treated brain suspension was mixed with 750 μl of pepsin solution (15 μM pepsin, 85 mM NaCl (Wako), 85 μM HCl (Wako)) and statically cultured at 37°C under 5% CO_2_ for 10 min. After pepsin treatment, the solution was mixed with 500 μl of neutralizer (1.2% sodium bicarbonate (Sigma-Aldrich), pH 8.3) and statically cultured for 10 min [27]. Finally, the suspension was filtrated through a filter unit of 5-μm pore size, centrifuged, suspended in culture medium (5 ml), and inoculated in HFFs.

### Phylogenetic tree analysis

To build phylogenetic trees, sequence data for uracil phosphoribosyl transferase (UPRT) intron 1, UPRT intron 7, hypothetical protein (HP) intron 2, dense granule protein 6 (GRA6), dense granule protein 7 (GRA7), and surface antigen gene 1 (SAG1) of representative strains were obtained from the GenBank (http://www.ncbi.nlm.nih.gov/). GenBank IDs of these sequences are described in S3 Table. We utilize the GenBank IDs of ME49, whose six loci are deposited in GenBank, to get the sequences not reposited in GenBank. Firstly, we got the accession number of the sequences of ME49 in ToxoDB (http://toxodb.org/toxo/) from the GenBank IDs of this strain. Next, we logged in each gene page in ToxoDB by using the accession number. We selected Show Alignment option in Isolate Alignments in this Gene Region section. In the output pages, we copied the interest parts of sequences not deposited in GenBank aligned with that of ME49 and pasted the sequence on the plain text to make a multi FASTA file of each locus finally (S1 File).

The genome of TgCatJpOk1-4, TgCatJpGi1/TaJ and TgCatJpTy1/k-3 was extracted using NucleoSpin^®^ Tissue (Macherey-Nagel) and the other genome of Japanese isolates were provided by Hisako Kyan. Six loci of Japanese isolates were amplified by PCR using the primer set (S1 Table) and read (Eurofins Genomics, Tokyo, Japan). Each sequence was aligned using MAFFT ver. 7 [28]. Phylogenetic analysis of the 6 sequences (3,641 bps) was conducted in RAxML ver. 8.1.5 using the maximum likelihood method and the GTR+Γ+I model [29]. This model was selected as the substitution model following a model test of IQ-TREE ver. 1.4.4 (http://www.iqtree.org/). Bootstrap analysis was conducted 1,000 times to evaluate the reliability of the phylogenetic tree.

### Population genetic analysis

Population genetic analysis of *T*. *gondii* was performed by STRUCTURE ver. 2.3.4 (http://pritchardlab.stanford.edu/structure.html) using linkage model. STRUCTURE was run with 100,000 burn-ins and 1,000,000 iterations, and the analysis was conducted 10 times on each K value (K = 2–10).

### Genome sequencing, assembly and analysis

The genome of TgCatJpOk3 and TgCatJpOk4 were read by Illumina Hiseq 2500 (Macrogen Japan, Kyoto, Japan) and Illumina Hiseq 2000 (Macrogen Japan), respectively. These genomic sequences were deposited in DDBJ Sequence Read Archive (DRA Accession; DRA008914 (TgCatJpOk3) and DRA007306 (TgCatJpOk4)). The pair-end reads were mapped to the *T*. *gondii* ME49 strain genome (release-28) obtained from ToxoDB using bowtie 2 [30]. The draft genome was visualized using Integrated Genomics Viewer ver. 2.3.4 (http://software.broadinstitute.org/software/igv/). ROP5, ROP16, and ROP18 gene sequences of TgCatJpOkJp3 and TgCatJpOkJp4 were aligned with those of archetypical strains (GT1, ME49 and VEG) using MAFFT ver. 7 [28]. These sequences are described in S2 File. Sequence reads of GT1 (SRR516419), ME49 (SRR6793863), VEG (SRR516406) downloaded from NCBI SRA database were mapped to the *T*. *gondii* ME49 strain genome (release-28) using bowtie 2 to analysis the sequence coverage around the ROP5 coding region (S3 File).

### Virulence assay in mice

*T*. *gondii* was diluted to 2.0×10^1^, 2.0×10^3^, and 2.0×10^5^ parasites/ml in 1 mM D-PBS with 100 μM CaCl_2_. The diluent (500 μl) was intraperitoneally inoculated in CD1 mice (8–12 weeks old, female, 5 per group) using a 27-G injection syringe (Terumo). In each group, we used 10 mice for the experiment, respectively. Survival and clinical signs were checked twice a day, and survival was recorded every day to analyze the statistics of survival curves. Moribund mice in a coma, with no movement, no body extension and no response to any stimulus, were euthanized by deep anesthesia of isoflurane (Merck, Whitehouse Station, NJ). No mouse died before meeting criteria for euthanasia. At 30 days after infection, surviving mice were sacrificed to minimize the pain. The mouse was euthanized with the same way mentioned above.

All animal experiments were conducted in accordance with the Guidelines for Animal Experimentation of the Japanese Association for Laboratory Animal Science and were approved by the Institutional Animal Care and Use Committee of the National Institute of Infectious Diseases (permission numbers: 115108). All surgery was performed under isoflurane anesthesia, and all efforts were made to minimize suffering. All the staff who carried out these animal experiments has taken the class for the animal care and handling by the Institutional Animal Care and Use Committee.

### Invasion assay

An invasion assay was performed using a previously described method [31].

### Tissue cyst formation

HFFs were incubated confluently on a cover slip and 1.0×10^4^ *T*. *gondii* were inoculated and statically cultured at 37°C for 5 days. The medium was replaced with medium to induce tissue cyst formation (RPMI1640 (Sigma-Aldrich), 1% FBS, 50 mM Hepes Buffer, 10 μg/mL gentamicin, pH 8.1) [32]. The tissue cyst was induced at 37°C in an air incubator for 7 days. The cyst wall was stained with fluorescein-labeled *Dolichos Biflorus* Agglutinin (Vector Labs, Burlingame, CA, USA), with observation using LSM510 (Carl Zeiss, Oberkochen, Germany).

## Results

### Phylogenetic analyses of Japanese isolates

**Fig 1:**
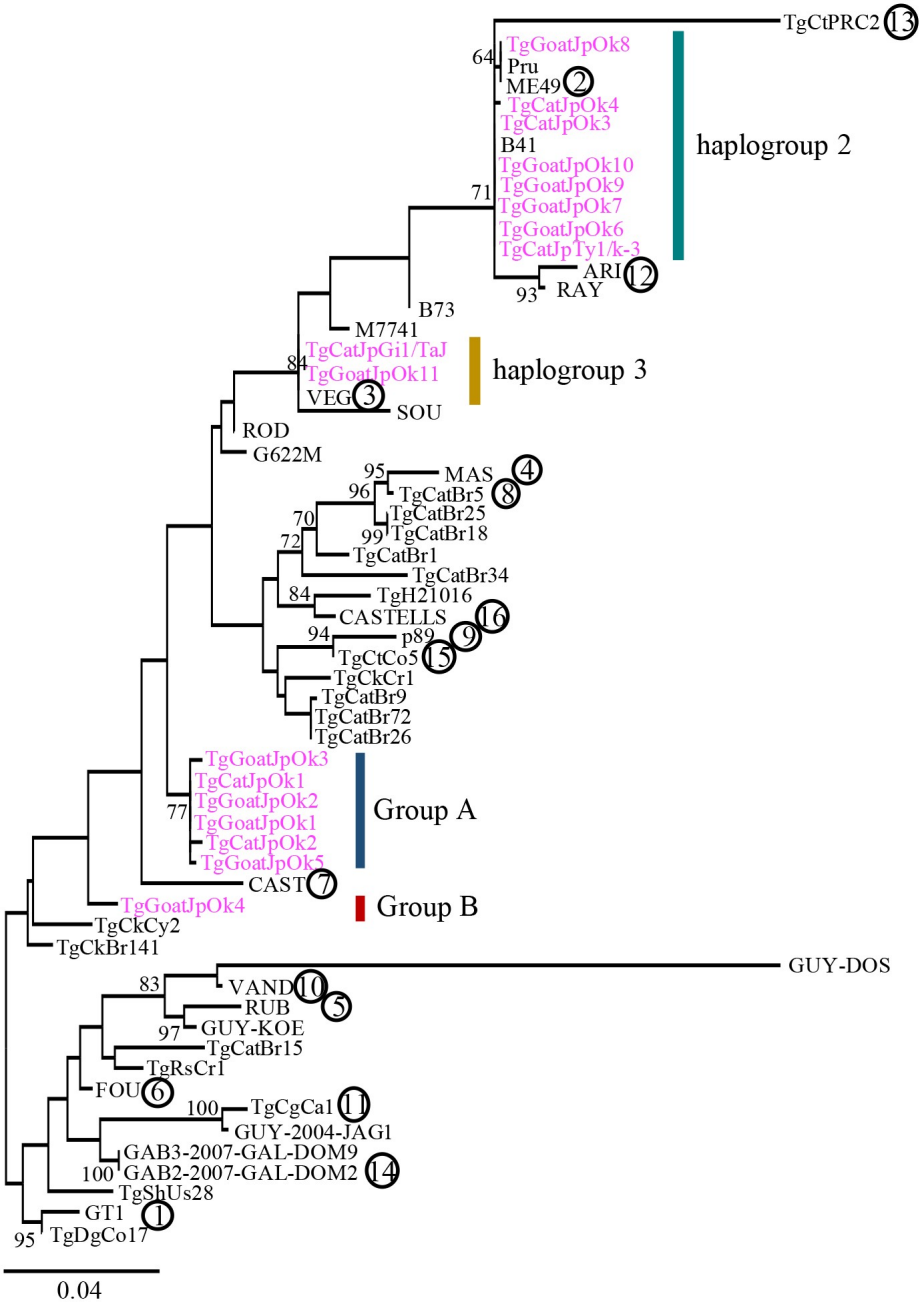
Phylogenetic analysis of Japanese wild isolates of *T*. *gondii*. Maximum likelihood analysis was performed using 3 genes and 3 intron sequences from 17 Japanese isolates and strains of 16 haplogroups. The number in the circle indicates representative strains of haplogroups. Detail information of other isolates is shown in S2 Table. Bootstrap value (BP) more than 60% is shown at nodes. Japanese isolates are shown in magenta.

To reveal phylogenetic position of *T*. *gondii* isolated from Japan, we conducted a phylogenetic analysis using the maximum likelihood method. A phylogenetic tree was constructed from DNA sequences of 3,641 bps from six loci (UPRT intron 1, UPRT intron 7, HP intron 2, GRA6, GRA7, SAG1), which are traditionally used for phylogenetic analysis, of 17 Japanese isolates (Table 1) and 43 strains of 16 haplogroups (Fig 1). A high or moderate bootstrap (BP) value at each node supported each clade containing haplogroups 2, 12 and 13 (BP = 71%), 4 and 8 (BP = 72%), and 5 and 10 (BP = 83%), respectively. This result showed the justification of use of six loci to construct phylogenetic tree because the clade formation was so similar to previous findings [13,24]. Japanese isolates were clustered into four genetically distinct groups. Eight isolates were sister to strains in haplogroups 2, although we could not determine the accurate phylogenetic position because of the low BP value. Group A formed a clade including only Japanese isolates with a moderate BP value (BP = 77%). TgCatJpGi1/TaJ and TgGoatJpOk11 were sisters to VEG of haplogroup 3 with a high BP value (BP = 84%) and were classified into haplogroup 3. Group B, which is composed of only TgGoatJpOk4, did not have the definitive phylogenetic position (BP = 35%).

**Table 1:** Japanese isolates used in this study.

Isolates	Isolated host	Isolated area	References
TgCatJpOk1	Cat	Okinawa island, Okinawa, Japan	Present study
TgCatJpOk2	Cat	Okinawa island, Okinawa, Japan	Present study
TgCatJpOk3	Cat	Okinawa island, Okinawa, Japan	Present study
TgCatJpOk4	Cat	Miyako island, Okinawa, Japan	Present study
TgCatJpTy1/k-3	Cat	Tokyo, Japan	[33]
TgCatJpGi1/TaJ	Cat	Gifu, Japan	[34]
TgGoatJpOk1	Goat	Okinawa island, Okinawa, Japan	[26]
TgGoatJpOk2	Goat	Okinawa island, Okinawa, Japan	[26]
TgGoatJpOk3	Goat	Okinawa island, Okinawa, Japan	[26]
TgGoatJpOk4	Goat	Okinawa island, Okinawa, Japan	[26]
TgGoatJpOk5	Goat	Okinawa island, Okinawa, Japan	[26]
TgGoatJpOk6	Goat	Okinawa island, Okinawa, Japan	[26]
TgGoatJpOk7	Goat	Okinawa island, Okinawa, Japan	[26]
TgGoatJpOk8	Goat	Okinawa island, Okinawa, Japan	[26]
TgGoatJpOk9	Goat	Okinawa island, Okinawa, Japan	[26]
TgGoatJpOk10	Goat	Okinawa island, Okinawa, Japan	[26]
TgGoatJpOk11	Goat	Okinawa island, Okinawa, Japan	[26]
TgGoatJpOk12	Goat	Okinawa island, Okinawa, Japan	[26]
TgGoatJpOk13	Goat	Okinawa island, Okinawa, Japan	[26]

**Fig 2:**
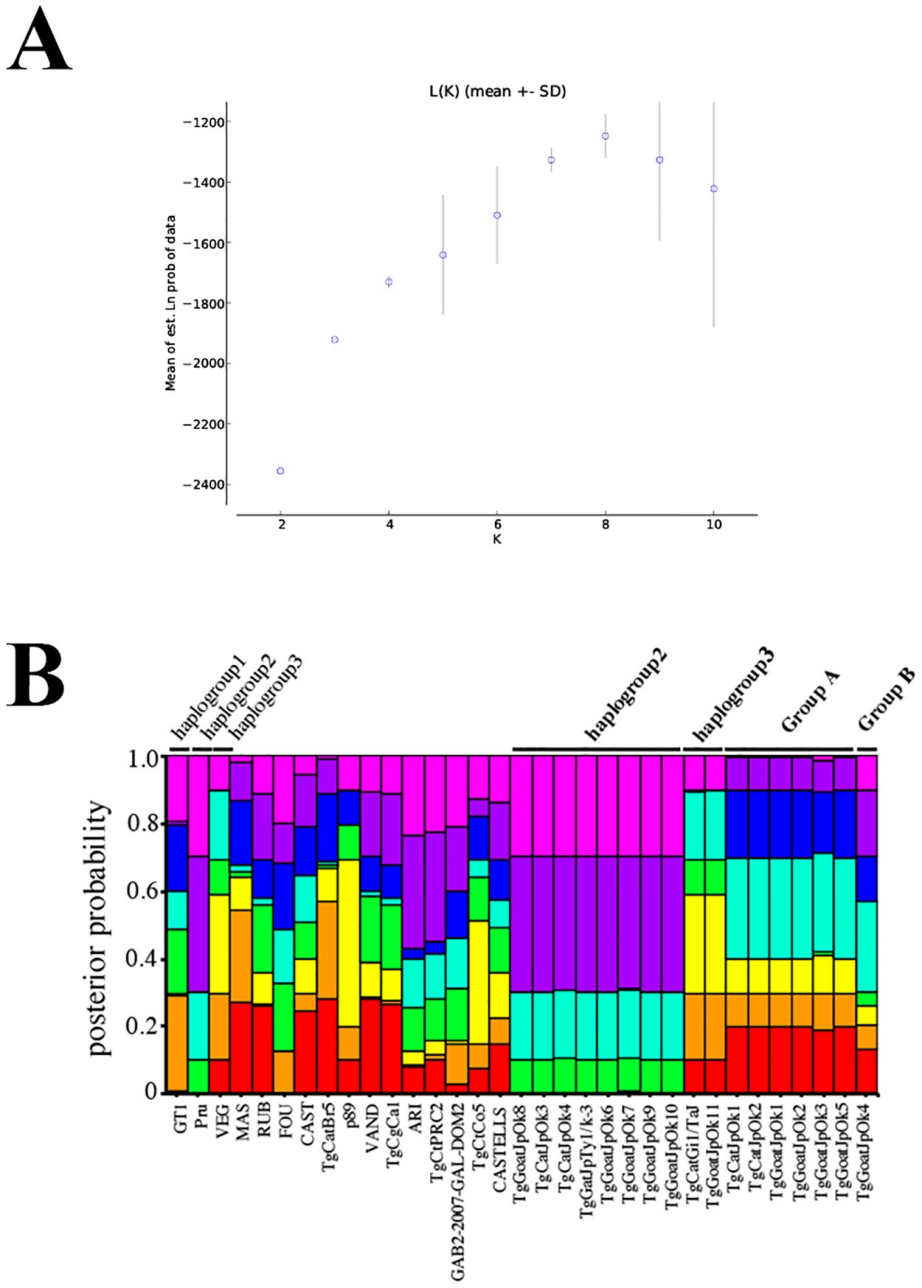
Population structural analysis of reference clones and Japanese isolates. (A) STRUCTURE program indicated that K = 8 is the most probable score, consistent with previous findings [13]. (B) Genetic composition of *T*. *gondii* for K = 8.

To further investigate phylogenetic relationships of Japanese isolates by comparison with those of representative strains, genetic composition analysis was conducted using STRUCTURE [35]. We determined K = 8 as the most probable score because the inflection point of L(K) was at K = 8 (Fig 2A), which means that the genetic structure of *T*. *gondii* comprises 8 different compositions. The estimation of the ancestral number was matched to that of previous study [13]. At K = 8, each strain of groups A and B had completely different component patterns from the other strains (Fig 2B). This result supports the classification of Japanese isolates on our phylogenetic tree analysis. Collectively, the phylogenetic analysis revealed that 16 Japanese isolates are classified into four group (group A and B, haplogroup 2 and 3) and there are clones of *T*. *gondii* with different genetic composition from known strains in Japan.

### Virulence of Japanese isolates in mice

**Fig 3:**
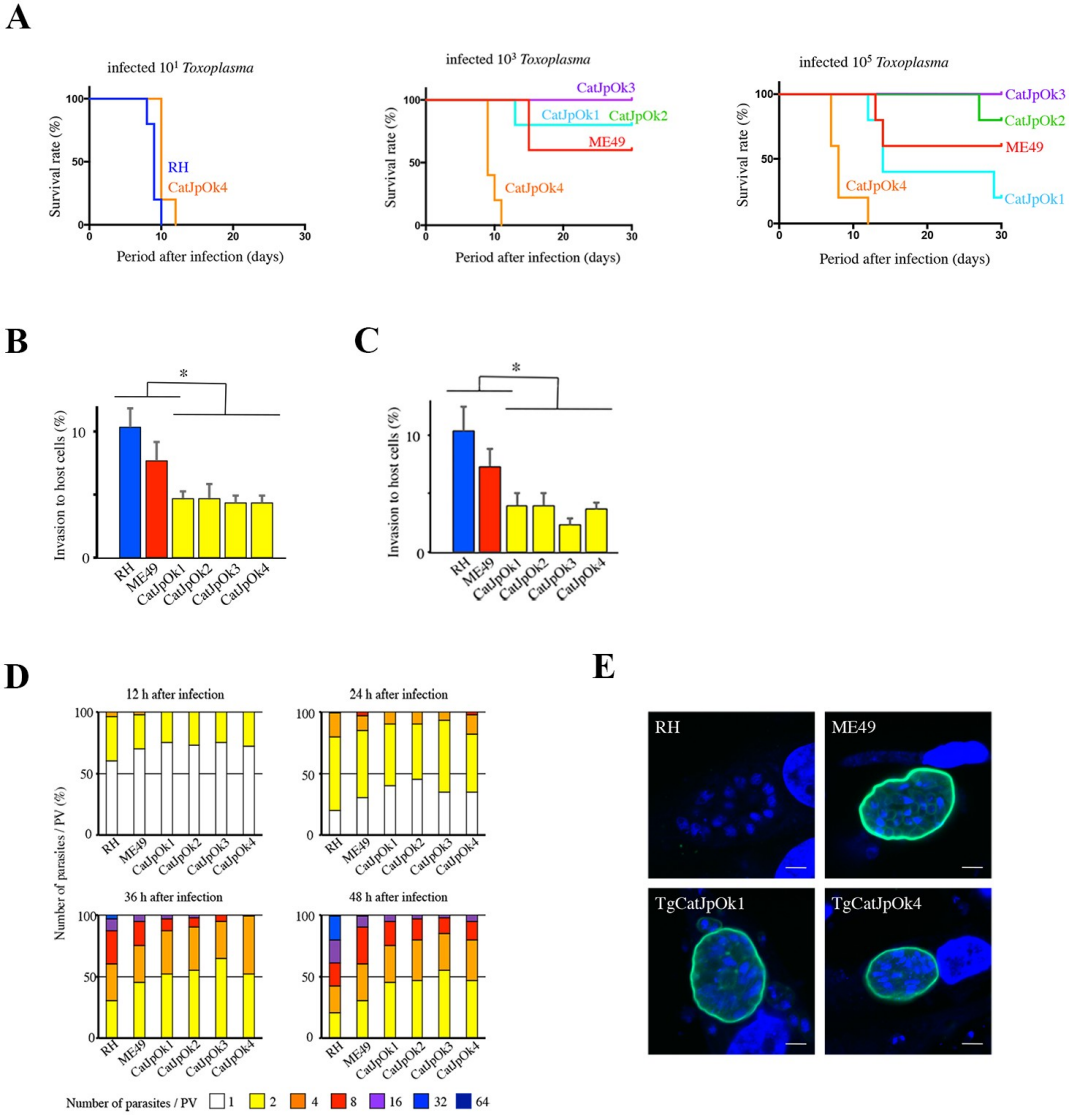
Virulence phenotypes of Japanese isolates of *T*. *gondii*. (A) Survival rates following intraperitoneal inoculation of 10^1^, 10^3^ and 10^5^ parasites in mice (n = 5 in each group). Survival was monitored for 30 days after inoculation. (B, C) Rates of invasion of host cells (B, HFFs; C, mouse embryonic fibroblasts (MEFs)) for 30 min after inoculation with a virulent haplogroup 1 strain (RH), low virulent haplogroup 2 strain (ME49), and Japanese isolates. Values are shown as mean + SEM, n = 3, * *p* < 0.05, Tukey-Kramer test. (D) Proliferative rates in HFFs infected with RH, ME49 and Japanese isolates determined by counting the number of parasites in parasitophorous vacuoles (n = 3). (E) Tissue cyst formation by RH, ME49, TgCatJpOk1 and TgCatJpOk4. The tissue cyst wall was stained by FITC-conjugated *Dolichos biflorus* agglutinin and is shown in green. Nuclei were stained with DAPI and are shown in blue. Scale bars = 5 μm.

To examine the virulence of the four Japanese isolates TgCatJpOk1 and TgCatJpOk2 (group A) and TgCatJpOk3 and TgCatJpOk4 (haplogroup 2) in mice, 10^1^, 10^3^, and 10^5^ *T*. *gondii* were inoculated into CD1 mice intraperitoneally and survival was monitored (Fig 3A). Mice inoculated with extremely virulent strain RH (haplogroup 1) and TgCatJpOk4 succumbed completely within about 10 days after injection of 10^1^ parasites. Therefore, we concluded that TgCatJpOk4 has almost equivalent virulence to the RH strain. All mice inoculated with TgCatJpOk3 survived after infection, even with 10^3^ and 10^5^ parasites, indicating that TgCatJpOk3 is avirulent within the range of infection of 10^5^ parasites, as observed for haplogroup 3 strains (avirulent). TgCatJpOk3 and TgCatJpOk4 were sister to strains of haplogroups 2 in the phylogenetic tree (Fig 1) but those of haplogroup 2 exhibit mild virulence in mice. Therefore, Japanese isolates classified into haplogroup 2 may have different propensity related to virulence from haplogroup 2 strains. The survival rates of mice inoculated with TgCatJpOk1 or TgCatJpOk2 were similar to that with ME49 (haplogroup 2 strain; moderately virulent), indicating modest virulence of these strains.

The variable virulence of *T*. *gondii* can be explained by differences in invasive and proliferative abilities in host cells. To investigate these properties, Japanese isolates (TgCatJpOk1-4) were compared with strains of haplogroups 1 and 2. The invasive capacity of four Japanese isolates was significantly lower than that of haplogroups 1 and 2 strains in human and mouse fibroblasts (Fig 3B and 3C). There was no significant difference in proliferative ability between each strain at 12 and 24 h after infection, but the number of parasites in a parasitophorous vacuole (PV) after 36 and 48 h in the Japanese isolates was less than in the haplogroup 1 parasite (Fig 3D), implying that the proliferative capacities of the Japanese isolates were lower than that of the haplogroup 1 strain. There was no marked difference between haplogroup 2 strains and Japanese isolates.

*T*. *gondii* can be infectious in humans in the form of tissue cyst. The capacity for tissue cyst formation varies among strains [36] and the well established RH laboratory strain lacks the ability of cyst formation. These results provoked us to check the ability of the Japanese isolates TgCatJpOk1 and TgCatJpOk4 to form tissue cysts. Following the induction of cyst formation, the membrane encircling these isolates was able to bind to *Dolichos biflorus* agglutinin, which recognizes specific glycosylation of CST1 protein associated with the cyst wall (Fig 3E). This result pointed out that these Japanese isolates have the ability to form cyst walls and transform into tissue cyst.

### Sequence variations of virulence and immunomodulatory factors of Japanese isolates

ROP5 and ROP18 are implicated in the virulence in mice and ROP16 interferes with the host immune system, which were revealed by QTL analysis on crosses of archetypical strains (types I, II and III) [15,16,20,22]. To elucidate the molecular mechanism underlying different virulence of TgCatJpOk3 (avirulent) and TgCatJpOk4 (high virulent) in mice from that of strains belonging to haplogroup2 (moderate virulent), we determined draft genome sequences of TgCatJpOk3 and TgCatJpOk4 to compare the sequences of the virulence genes with those of types I-III (Table 2).

**Table 2:** Quality data of whole-genome-sequence and assembly.

	Quality scores	
	TgCatJpOk3	TgCatJpOk4
Reference size (bp)	65,668,596	65,668,596
Read length (bp)	101	101
Number of reads	101,796,604	64,876,556
Mapped reads	82,638,024	58,986,960
Mean Coverage ± SD	124.6499 ± 84.7693	90.6966 ± 72.5837
GC Percentage (%)	51.55	51.27

**Fig 4:**
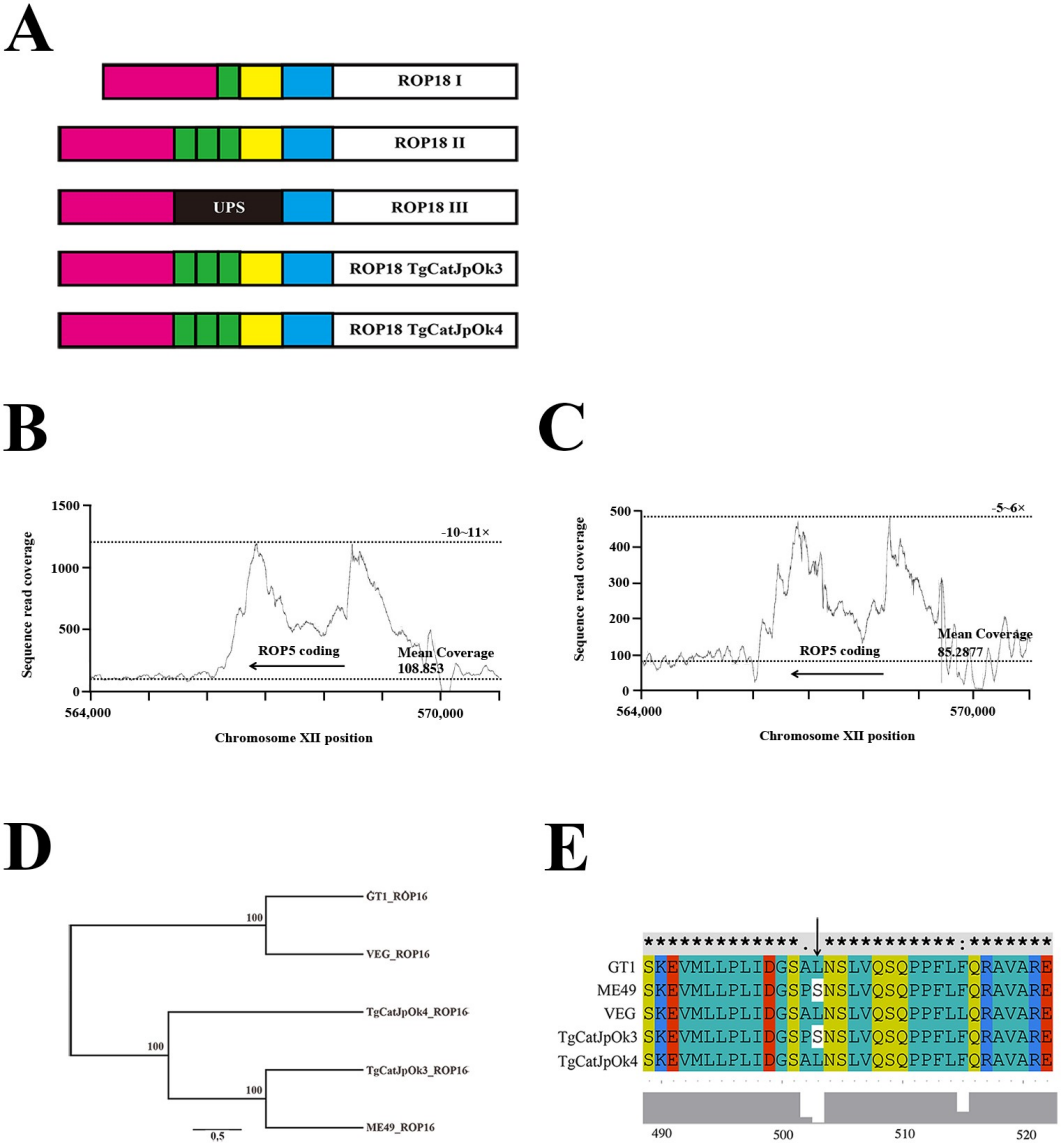
Comparison of TgCatJpOk4 genes responsible for virulence of *T*. *gondii* with those in 3 archetypical strains. (A) Schematic of the ROP18 gene sequence. UPS; upstream segment (B,C) Copy number of ROP5. The copy number is indicated by the read coverage of TgCatJpOk3 (B) and TgCatJpOk4 (C) around the ROP5 coding region on the ME49 genome (reference genome). Arrows show the coding region of ROP5 on the chromosome XII. Mean coverages indicate the average depth in TGME49_chrXII:564000–566000, whose position was adjacent to the ROP5 coding region. (D) Phylogenetic analysis of the ROP16 amino acid sequence of TgCatJpOk3, TgCatJpOk4 and type I (GT1), type II (ME49), and type III (VEG) strains. (E) Alignment of amino acid sequences from 489 to 522 of ROP16. The arrow shows the leucine residue that is required for activation of STAT3 [23].

The insertion sequence in the promoter region of ROP18 in type III strains inhibits transcription of the gene and the number of transcriptions is correlated with a virulence of *T*. *gondii* in mice [37]. To evaluate the sequence of this promoter region in TgCatJpOk3 and TgCatJpOk4, we aligned the sequences to those of type I-III strains. The sequence of the TgCatJpOk3 promoter was coincident with that of type II strains (Fig 4A), which is reasonable considering their phylogenetic proximity (Fig 1). Similarly, sequence of the TgCatJpOk4 promoter was congruent with that of type II strains. Collectively, these findings indicate that TgCatJpOk3 and TgCatJpOk4 ROP18 are active considering the previous report [37].

The gene for ROP5 is composed of a tandem repeat in the *T*. *gondii* genome [20]. The type II strain has a longer repeat (9 copies) than the type I and III strains (6 copies), and type II ROP5 does not serve as a virulence factor [20]. We investigated the genetic structure of ROP5 in TgCatJpOk3 and TgCatJpOk4 draft genome. The copy number of ROP5 in TgCatJpOk3 was similar to that of types II, considering the sequence coverage around the ROP5 coding region (Fig 4B and S3 File). Whereas the sequence depth around the ROP5 coding region was only several times higher than the mean coverage of the genome, similar to types I and III (Fig 4C and S3 File). The amino acid at position 389 of type I and III ROP5 (virulent types) is arginine in 5 isoforms and histidine in one isoform, but the amino acid at this position of type II ROP5 (avirulent types) is arginine in all isoforms [20]. Considering the previous study referring to ROP5 isoforms [20] and the base composition in read sequences of TgCatJpOk3 and TgCatJpOk4 coding for an amino acid at position 389 of ROP5 (Table 3), it might be possible to infer that 83% of isoforms have arginine and 17% of isoforms have histidine at position 389 of TgCatJpOk4 ROP5, whereas all isoforms have arginine at this position of TgCatJpOk3 ROP5. In conclusion, the repeat pattern and base composition indicated that ROP5 gene was an avirulent type in TgCatJpOk3 and a virulent type in TgCatJpOk4.

**Table 3:** The base composition in read sequences of TgCatJpOk3 and TgCatJpOk4 encoding an amino acid at position 389 of ROP5.

	Nucleotide positions in ROP5		
	1165	1166	1167
TgCatJpOk3	C (100%)	G (100%)	C (100%)
TgCatJpOk4	C (100%)	A (83%)	T (83%)
		G (17%)	C (17%)

ROP16 disturbs the IFN-γ signaling pathway and modulates host immune response in infection with *T*. *gondii* [22,38]. Type I or III ROP16 can phosphorylate and activate STAT3 and STAT6 during infection, whereas type II ROP16 does not have this effect. Leucine at position 503 of type I or III ROP16 is essential for activation of STAT3 through phosphorylation, and a type II ROP16 mutant with serine-503 replaced by leucine can activate STAT3 [23]. An alignment of the amino acid sequence of ROP16 of TgCatJpOk3 and TgCatJpOk4 showed that these sequences are close to that of type II ROP16 (Fig 4D). However, the 503 position of TgCatJpOk4 ROP16 is leucine although that of TgCatJpOk3 ROP16 is serine (Fig 4E). This result suggests that TgCatJpOk4 ROP16 can activate STAT3 through phosphorylation but TgCatJpOk3 ROP16 can not do so.

## Discussion

We used six sequences (UPRT intron 1, UPRT intron 7, HP intron 2, GRA6, GRA7, SAG1) in our phylogenetic analysis. The topology of phylogenetic tree reconstructed well that of previous study using the data of genome-wide SNPs of various strains of *T*. *gondii* [13]. However, our dataset formed a clade that differed partially from a previous study [13]. The different dataset and method (maximum likelihood estimation) may have caused this discrepancy. Similarly, STRUCTURE analysis estimated the ancestral number (K = 8) matched to the number previous reported [13] but representative strains had much more genetic components than previously reported, probably due to use of different data set.

The phenotyping analysis of four Japanese isolates revealed that TgCatJpOk3 and TgCatJpOk4 exhibited different virulence in mice from strains of haplogroup 2 (avirulent or high virulent, respectively) although these strains were classified into haplogroup 2, whose strains exhibit moderate virulence in mice. The genomic comparative analysis showed the virulent difference between TgCatJpOk4 and strains of haplogroup 2 was explained partially by the copy number of ROP5 and mutation at position 503 of ROP16. Whereas a difference of virulence between TgCatJpOk3 and strains of haplogroup 2 was not identified in this analysis. Considering the type of virulence and immunomodulatory factors, TgCatJpOk4 may adopt a strategy similar to that of haplogroup 1 to survive in Japanese host and environment. The cause of the low invasive and proliferative capacity of TgCatJpOk4 could not be understood at the molecular level in this analysis. TgCatJpOk4 has potential for differentiation to a tissue cyst, in contrast to the RH strain (haplogroup 1). This difference may be attributed to distinct *in vitro* phenotypes in TgCatJpOk4 and RH strain.

We questioned whether mutation at position 503 of ROP16 of TgCatJpOK4 is caused by a drifted variant occurring in type II ROP16 or a genetic recombination in sexual crosses between strains of haplogroup 2 and other strains. We performed a BLAST search using amino acid sequence of TgCatJpOK4 ROP16. As a result, the sequence of *T*. *gondii* ARI strain which consists of haplogroup 12 was matched completely with that of TgCatJpOK4 (100% identity). Therefore, the mutation of TgCatJpOK4 ROP16 is possibly derived from that of haplogroup 12.

TgCatJpOK3 and TgCatJpOK4 were classified into haplogroup 2 but these strains showed different phenotypes from strains of haplogroup 2. Especially, TgCatJpOK4 has different variance of ROP5 and ROP16. However, the resolution of our phylogenetic analysis could not divide these strains into other groups. Therefore, whole genome sequence analysis is required in a further study to decide accurately phylogenetic position of these isolates. Recently, the whole genome sequences of 62 clones were reported [24]. This result and our information are likely to be useful for further analysis of phylogeny of *T*. *gondii* and for development of medical and veterinary remedies for parasitic disease.

## Supporting information

S1 Table Primer sets used in this study. (XLSX)

S2 Table Clones used to construct phylogenetic tree in this study. (XLSX)

S3 Table Accession number of each loci in NCBI used to construct phylogenetic tree in this study. All sequences of loci marked with asterisks (*) are not reposited in NCBI. We got the sequences from alignment of TGME49_211630 or TGME49_312480 with that of the other strains using an alignment tool in Isolate Alignments in this Gene Region section of ToxoDB (http://toxodb.org/toxo/). Abbreviations are shown as dense granule protein 6; GRA6, dense granule protein 7; GRA7, surface antigen gene 1; SAG1, hypothetical protein intron 2; HP2, uracil phosphoribosyl transferase intron 1; UPRT1 and uracil phosphoribosyl transferase intron 7; UPRT7.
(XLSX)

S1 File Sequences of 6 loci in each strain used to build a phylogenetic tree. (PDF)

S2 File Sequences of ROP5, ROP16 and ROP18 in TgCatJpOk3 and TgCatJpOk4. It is noted that bases in the coding sequences are shown in bold letters.
(PDF)

S3 File Sequence coverages around the ROP5 coding region in GT1, ME49 and VEG. (A,B,C) Sequence coverages around the ROP5 coding region in GT1 (A), ME49 (B) and VEG (C) are shown. Arrows indicate the coding region of ROP5 on the chromosome XII.
(TIF)
