# Supplementary material for: Molecular and biological analysis revealed genetic diversity and high virulence strain of Toxoplasma gondii in Japan
Source: PLoS One. 2020 Feb 3;15(2):e0227749. doi: 10.1371/journal.pone.0227749 (PMC6996823; doi:10.1371/journal.pone.0227749)
Supplement: S1 File — (PDF) [file pone.0227749.s004.pdf]

Dense granule protein 6 (GRA6)

>GT1
ATGGCACACGGTGGCATCCATCTGAGGCAGAAGCGTAACTTCTGTCCT
GTAACTGTCTCCACAGTTGCTGTGGTCTTTGTAGTCTTCATGGGTGTA
CTCGTCAATTCGTTGGGTGGAGTCGCTGTCGCAGCAGACAGCGGTGG
TGTTAAGCAGACCCCTTCGGAAACCGGTTCGAGCGGTGGACAGCAAGA
AGCAGTGGGGACCACTGAAGACTATGTCAACTCTTCGGCGATGGGCG
GTGGCCAAGGCGACTCGTTAGCTGAAGATGATACAACCTCCGAAGCGG
CGGAGGGCGACGTTGACCCTTTTCCCGTGCTGGCGAATGAGGGGAAG
TCGGAGGCGCGTGGCCCGTCGCTCGAGGAAAGAATCGAAGAACAGGG
CACAAGACGACGTTACTCCTCTGTTCAAGAACCACAAGCGAAGGTGCC
TAGCAAACGAACACAGAAACGCCACAGACTCATTGGTGCTGTGGTGTT
GGCAGTATCTGTGGCAATGCTTACCGCTTTCTTTCTTCGAAGGACTGG
ACGACGCTCTCCCCAAGAACCATCTGGGGATGGTGGTGGAAATGATGC
AGGCAATAATGCTGGGAACGGTGGGAATGAAGGCAGAGGTTACGGAG
GCAGAGGTGAAGGAGGAGCCGAGGATGACAGGCGCCCGTTGCACCCG
GAACGTGTGAATGTGTTTGATTATTAA

>ME49
ATGGCACACGGTGGCATCTATCTGAGGCAGAAGCGTAACTTCTGTCCT
TTAACTGTCTCCACAGTTGCTGTGGTCTTTGTAGTCTTCATGGGTGTA
CTCGTCAATTCGTTGGGTGGAGTCGCTGTCGCAGCAGACAGCGGTGG
TGTTAGGCAGACCCCTTCGGAAACCGGTTCGAGCGGTGGACAGCAAG
AAGCAGTGGGGACCACTGAAGACTATGTCAACTCTTCGGCGATGGGC
GGTGGCCAAGGCGACTCGTTAGCTGAAGATGATACAACCTCCGATGCG
GCGGAGGGCGACGTTGACCCTTTTCCCGCGCTGGCGAATGAGGGGAA
GTCGGAGGCGCGTGGCCCGTCGCTCGAGGAAAGAATCGAAGAACAGG
GCACAAGACGACGTTACTCCTCTGTTCAAGAACCACAAGCGAAGGTGC
CTAGCAAACGAACACAGAAACGCCACAGACTCATTGGTGCTGTGGTGT
TGGCAGTATCTGTGGCAATGCTTACCGCTTTCTTTCTTCGAAGGACTG
GACGACGCTCTCCCCAAGAACCATCTGGGGGTGGTGGTGGAAATGAT
GCAGGCAATAATGCTGGGAACGGTGGGAATGAAGGCAGAGGTGAAGG
AGGCGAGGATGACAGGCGCCCGTTGCACCCGGAAGTGTGAATGAGT
TTGATTTTTAA

>VEG
ATGGCACACGGTGGCATCCATCTGAGGCAGAAGCGTAACTTCTGTCCT
TTAACTGTCTCCACAGTTGCTGTGGTCTTTGTAGTTTTCATGGGTGTA
CTCGTCAATTCGTTGGGTGGAGTCGCTGTCGCAGCAGACAGCGATGG
TGTTAAGCAGACCCCTTCGGAAACCGGTTCGAGCGGTGGACAGCAAGA
AGCAGTGGGGACCACTGAAGACTATGTCAACTCTTCGGCGATGGGCG
GTGGCCAAGGCGACTCGTTAGCTGAAGATGATACAACCTCCGATGCGG
CGGAGGGCGACGTTGACCCTTTTCCCGTGCTGGCGAATGAGGGGAAG
TCGGAGGCGCGTGGCCCGTCGCTCGAGGAAAGAATCGAAGAACAGGG
CACAAGACGACGTTACTCCTCTGTTCAAGAACCACAAGCGAAGGTGCC
TAGCAAACGAACACAGAAACGCCACAGACTCATTGGTGCTGTGGTGTT
GGCAGTATCTGTGGCAATGCTTACCGCTTTCTTTCTTCGAAGGACTGG
ACGACGCTCTCCCCCAGAACCATCTGGGGATGGTGGTGGAAATGATG
CAGGCAATAATGCTGGGAACCGTGGGAATGAAGGCAGAGGTTACGGA
GGCAGAGGTGAAGGAGGAGGCGAGGATGACAGGCGCGCGTTGCACCC
GGAACGTGTGAATGTGTTTGATTATTAA

>MAS
ATGGCACACGGTGGCATCTATCTGAGGCAGAAGCGTAACTTCTGTCCT
TTAACTGTCTCCACAGTTGCTGTGGTCTTTGTAGTCTTCATGGGTGTA
CTCGTCAATTCGTTGGGTGGAGTCGCTGTCGCAGCAGACAGCGGTGG
TGTTAAGCAGACCCCTTCGGAAACCGGTTCGAGCGGTGGACAGCAAGA
AGCAGTGGGGACCACTGAAGACTATGTCAACTCTTCGGCGATGGGCG
GTGGCCAAGGCGACTCGTTAGCTGAAGATGATACAACCTCCGATGCGG
CGGAGGGCGACGTTGACCCTTTTCCCGCGCTGGCGAATGAGGGGAAG
TCGGAGGCGCGTGGCCCGTCGCTCGAGGAAAGAATCGAAGAACAGGG
CACAAGACGACGTTACTCCTCTGTTCAAGAACCACAAGCGAAGGTGCC
TAGCAAACGAACACAGAAACGCCACAGACTCATTGGTGCTGTGGTGTT
GGCAGTATCTGTGGCAATGCTTACCGCTTTCTTTCTTCGAAGGACTGG
ACGACGCTCTCCCCAAGAACCATCTGGGGGTGGTGGTGGAAATGATG
CAGGCAATAATGCTGGGAACGGTGGGAATGAAGGCAGAGGTTACGGA
GGCAGAGGTGAAGGAGGCGAGGATGGCAGGGGCCCGTTGCACCCGG
GAAGTGTGAATGAGTTTGATTTTTAA

>RUB
ATGGCACACGGTGGCATCCATCTGAGGCAGAAGCGTAACTTCTGTCCT

TTAACTGTCTCCACAGTTGCTGTGGTCTTTGTAGTCTTCATGGGTGTA
CTCGTCAATTCGTTGGGTGGAGTCGCTGTCGCAGCAGACAGCGGTGG
TGTTAAGCAGACCCCTTCGGAAACCGGTTTGAGCGGTGGACAGCAAGA
AGCAGTGGGGACCACTGAAGACTATGTCAACTCTTCGGCGATGGGCG
GTGGCCAAGGCGACTCGTTAGCTGAAGATGATACAACCTCCGATGCGG
CGGAGGGCGACGTTGACCCTTTTCCCGTGCTGGCGAATGAGGGGAAG
TCGGAGGCGCGTGGCCCGTCGCTCGAGGAAAGAATCGAAGAACAGGG
CACAAGACGACGTTACTCCTCTGTTCAAGAACCACAAGCGAAGGTGCC
TAGCAAACGAACACAGAAACGCCACAGACTCATTGGTGCTGTGGTGTT
GGCAGTATCTGTGGCAATGCTTACCGCTTTCTTTCTTCGAAGGACTGG
ACGACGCTCTCCCCCAGAACCATCTGGGCATGGTGGTGGAAATGATGC
AGGCAATAATGCTGGGAACGGTGGGAATGAAGGCAGAGGTTACGGAG
GCAGAGGTGAAGGAGGAGGCGAGGATGACAGGCGCGCGTTGCACCTG
GAACGTGTGAATGCGTTTGATTATTAA

>FOU
ATGGCACACGGTGGCATCCATCTGAGGCAGAAGCGTAACTTCTGTCCT
TTAACTGTCTCCACAGTTGCTGTGGTCTTTGTAGTCTTCATGGGTGTA
CTCGTCAATTCGTTGGGTGGAGTCGCTGTCGCAGCAGACAGCGGTGG
TGTTCAGCAGACCCCTTCGGAAACCGGTTCGAGCGGTGGACAGCAAG
AAGCAGTGGGGACCACTGAAGACTATGTCAACTCTTCGGCGATGGGC
GGTGGCCAAGGCGACTCGTTAGCTGAAGATGATACAACCTCCGATGCG
GCGGAGGGCGACGTTGACCCTTTTCCCGTGCTGGCGAATGAGGGGAA
GTCGGAGGCGCGTGGCCCGTCGCTCGAGGAAAGAATCGAAGAACAGG
GCACAAGACGACGTTACTCCTCTGTTCAAGAACCACAAGCGAAGGTGC
CTAGCAAACGAACACAGAAACGCCACAGACTCATTGGTGCTGTGGTGT
TGGCAGTATCTGTGGCAATGCTTACCGCTTTCTTTCTTCGAAGGACTG
GACGACGCTCTCCCCCAGAACCATCTGGGGATGGTGGTGGAAATGAT
GCAGGCAATAATGCTGGGAACGGTGGGAATGAAGGCAGAGGTTACAG
AGGCAGAGGTGAAGGAGGAGGCGAGGATGACGGGCGCGCGTTGCACC
CGGAACGTGTGAATGTGTTTGATTATTAA

>CAST
ATGGCACACGGTGGCATCCATCTGAGGCAGAAGCGTAACTTCTGTCCT
GTAACTGTCTCCACAGTTGCTGTGGTCTTTGTAGTCTTCATGGGTGTA
CTCGTCAATTCGTTGGGTGGAGTCGCTGTCGCAGCAGACAGCGGTGG

TGTTAAGCAGACCCCTTCGGAAACCGGTTCGAGCGGTGGACAGCAAGA
AGCAGTGGGGACCACTGAAGACTATGTCAACTCTTCGGCGATGGGCG
GTGGCCAAGGCGACTCGTCAGCTGAAGATGATACAACCTCCGAAGCGG
CGGAGGGCGACGTTGACCCTTTTCCCGTGCTGGCGAATGAGGGGAAG
TCGGAGGCGCGTGGCCCGTCGCTCGAGGAAAGAATCGAAGAACAGGG
CACAAGACGACGTTACTCCTCTGTTCAAGAACCACAAGCGAAGGTGCC
TAGCAAACGAACACAGAAACGCCACAGACTCATTGGTGCTGTGGTGTT
GGCAGTATCTGTGGCAATGCTTACCGCTTTCTTTCTTCGAAGGACTGG
ACGACGCTCTCCCCAAGAACCATCTGGGGATGGTGGTGGAAATGATGC
AGGCAATAATGCTGGGAACGGTGGGAATGAAGGCAGAGGTTACGGAG
GCAGAGGTGAAGGAGGAGCCGAGGATGACAGGCGCCCGTTGCACCCG
GAACGTGTGAATGTGTTTGATTATTAA

>TgCatBr5
ATGGCACACGGTGGCATCTATCTGAGGCAGAAGCGTAACTTCTGTCCT
TTAACTGTCTCCACAGTTGCTGTGGTCTTTGTAGTCTTCATGGGTGTA
CTCGTCAATTCGTTGGGTGGAGTCGCTGTCGCAGCAGACAGCGGTGG
TGTTAAGCAGACCCCTTCGGAAACCGGTTCGAGCGGTGGACAGCAAGA
AGCAGTGGGGACCACTGAAGACTATGTCAACTCTTCGGCGATGGGCG
GTGGCCAAGGCGACTCGTTAGCTGAAGATGATACAACCTCCGATGCGG
CGGAGGGCGACGTTGACCCTTTTCCCGCGCTGGCGAATGAGGGGAAG
TCGGAGGCGCGTGGCCCGTCGCTCGAGGAAAGAATCGAAGAACAGGG
CACAAGACGACGTTACTCCTCTGTTCAAGAACCACAAGCGAAGGTGCC
TAGCAAACGAACACAGAAACGCCACAGACTCATTGGTGCTGTGGTGTT
GGCAGTATCTGTGGCAATGCTTACCGCTTTCTTTCTTCGAAGGACTGG
ACGACGCTCTCCCCAAGAACCATCTGGGGGTGGTGGTGGAAATGATG
CAGGCAATAATGCTGGGAACGGTGGGAATGAAGGCAGAGGTTACGGA
GGCAGAGGTGAAGGAGGCGAGGATGGCAGGGGCCCGTTGCACCCGG
GAAGTGTGAATGAGTTTGATTTTTAA

>p89
ATGGCACACGGTGGCATCCATCTGAGGCAGAAGCGTAACTTCTGTCCT
TTAACTGTCTCCACAGTTGCTGTGGTCTTTGTAGTCTTCATGGGTGTA
CTCGTCAATTCGTTGGGTGGAGTCGCTGTCGCAGCAGACAGCGATGG
TGTTAAGCAGACCCCTTCGGAAACCGGTTCGAGCGGTGGACAGCAAGA
AGCAGTGGGGACCACTGAAGACTATGTCAACTCTTCGGCGATGGGCG

GTGGCCAAGGCGACTCGTTAGCTGAAGATGATACAGCCTCCGATGCGG
CGGAGGGCGACGTTGACCCTTTTCCCGTGCTGGCGAATGAGGGGAAG
TCGGAGGCGCGTGGCCCGTCGCTCGAGGAAAGAATCGAAGAACAGGG
CACAAGACGACGTTACTCCTCTGTTCAAGAACCACAAGCGAAGGTGCC
TAGCAAACGAACACAGAAACGCCACAGACTCATTGGTGCTGTGGTGTT
GGCAGTATCTGTGGCAATGCTTACCGCTTTCTTTCTTCGAAGGACTGG
ACGACGCTCTCCCCCAGAACCATCTGGGGATGATGGTGGAAATGATGC
AGGCAATAATGCTGGGAACGGTGGAATGAAGGCAGAGGTGAAGGAG
GAGGCGAGGATGACAGGCGCGCGTTGCACCCGGGAAGTGTGAATGTG
TTTGATTATTAA

>VAND
ATGGCACACGGTGGCATCCATCTGAGGCAGAAGCGTAACTTCTGTCCT
TTAACTGTCTCCACAGTTGCTGTGGTCTTTGTAGTCTTCATGGGTGTA
CTCGTCAATTCGTTGGGTGGAGTCGCTGTCGCAGCAGACAGCGGTGG
TGTTAAGCAGACCCCTTCGGAAACCGGTTCGAGCGGTGGACAGCAAGA
AGCAGTGGGGACCACTGAAGACTATGTCAACTCTTCGGCGATGGGCG
GTGGCCAAGGCGACTCGTTAGCTGAAGATGATACAACCTCCGATGCGG
CGGAGGGCGACGTTGACCCTTTTCCCGTGCTGGCGAATGAGGGGAAG
TCGGAGGCGCGTGGCCCGTCGCTCGAGGAAAGAATCGAAGAACAGGG
CACAAGACGACGTTACTCCTCTGTTCAAGAACCACAAGCGAAGGTGCC
TAGCAAACGAACACAGAAACGCCACAGACTCATTGGTGCTGTGGTGTT
GGCAGTATCTGTGGCAATGCTTACCGCTTTCTTTCTTCGAAGGACTGG
ACGACGCTCTCCCCCAGAACCATCTGGGGATGATGGTGGAAATGATGC
AGGCAATAATGCTGGGAACGGTGGAATGAAGGCAGAGGTTACAGAG
GCAGAGGTGAAGGAGGAGGCGAGGATGACAGGCGCGCGTTGCACCCG
GAACGTGTGAATGTGTTTGATTATTAA

>TgCgCa1
ATGGCACACGGTGGCATCTATCTGAGGCAGAAGCGTAACTTCTGTCCT
TTAACTGTCTCCACAGTTGCTGTGATCTTTGTAGTCTTCATGGGTGTA
CTCGTCAATTCGTTGGGTGGAGTCGCTTTCGCAGCAGACAGCGGTGG
TGTTAGGCAGACCCCTTCGGAAACCGGTTCGAGCGGTGGACAGCAAG
AAGCAGTGGGGACCACTGAAGACTATGTCAACTCTTCGGCGATGGGC
GGTGGCCAAGGCGACTCGTTAGCTGAAGATGATGATACAGCCTCCGAT
GCGGCGGAGGGCGACGTTGACCCTTTTCCCGCGCTGGCGAATGAGG

GGAAGTCGGAGGCGCGTGGCCCGTCGCTCGAGGAAAGAATCGAAGAA
CAGGGCACAAGACGACGTTATTCCTCTGTTCAAGAACCACAAGCGAAG
GTGCCTAGCAAACGAACACAGAAACGCCACAGACTCATTGGTGCTGTG
GTGTTGGCAGTATCTGTGGCAATGCTTACCGCTTTCTTTCTTCGAAGG
ACTGGACGACGCTCTCCCCAAGAACTATCTGGGGGTGGTGGTGGAAA
TGATGCAGGCAATAATGCTGGGAACGGTGGGAATGAAGGCAGAGGTG
ACGGAGGCAGAGGTGAAGGAGGAGGCGAGGATGACAGGCGCCCGTTG
CACCCGGAACGTGTGAATGAGTTTGATTATTAA

>ARI
ATGGCACACGGTGGCATCTATCTGAGGCAGAAGCGTAACTTCTGTCCT
TTAACTGTCTCCACAGTTGCTGTGATCTTTGTAGTCTTCATGGGTGTA
CTCGTCAATTCGTTGGGTGGAGTCGCTTTCGCAGCAGACAGCGGTGG
TGTTAGGCAGACCCCTTCGGAAACCGGTTCGAGCGGTGGACAGCAAG
AAGCAGTGGGGACCACTGAAGACTATGTCAACTCTTCGGCGATGGGC
GGTGGCCAAGGCGACTCGTTAGCTGAAGATGATGATACAGCCTCCGAT
GCGGCGGAGGGCGACGTTGACCCTTTTCCCGCGCTGGCGAATGAGG
GGAAGTCGGAGGCGCGTGGCCCGTCGCTCGAGGAAAGAATCGAAGAA
CAGGGCACAAGACGACGTTATTCCTCTGTTCAAGAACCACAAGCGAAG
GTGCCTAGCAAACGAACACAGAAACGCCACAGACTCATTGGTGCTGTG
GTGTTGGCAGTATCTGTGGCAATGCTTACCGCTTTCTTTCTTCGAAGG
ACTGGACGACGCTCTCCCCAAGAACCATCTGGGGGTGGTGGTGGAAA
TGATGCAGGCAATAATGCTGGGAACGGTGGGAATGAAGGCAGAGGTG
AAGGAGGAGGCGAGGATGACAGGCGCCCGTTGCACCCGGAACGTGTG
AATGAGTTTGATTATTAA

>TgCtPRC2
AGGCACACGGTGGCATCTATCTGAGGCAGAAGCGTAACTTCTGTCCTT
TAACTGTCTCCACAGTTGCTGTGGTCTTTGTAGTCTTCATGGGTGTAC
TCGTCAATTCGTTGGGTGGAGTCGCTGTCGCAGCAGACAGCGGTGGT
GTTAGGCAGACCCCTTCGGAAACCGGTTCGAGCGGTGGACAGCAAGA
AGCAGTGGGGACCACTGAAGACTATGTCAACTCTTCGGCGATGGGCG
GTGGCCAAGGCGACTCGTTAGCTGAAGATGATACAACCTCCGATGCGG
CGGAGGGCGACGTTGACCCTTTTCCCGCGCTGGCGAATGAGGGGAAG
TCGGAGGCGCGTGGCCCGTCGCTCGAGGAAAGAATCGAAGAACAGGG
CACAAGACGACGTTACTCCTCTGTTCAAGAACCACAAGCGAAGGTGCC

TAGCAAACGAACACAGAAACGCCACAGACTCATTGGTGCTGTGGTGTT
GGCAGTATCTGTGGCAATGCTTACCGCTTTCTTTCTTCGAAGGACTGG
ACGACGCTCTCCCCAAGAACCATCTGGGGGTGGTGGTGGAAATGATG
CAGGCAATAATGCTGGGAACGGTGGGAATGAAGGCAGAGGTGAAGGA
GGCGAGGATGACAGGCGCCCGTTGCACCCGGGAAGTGTGAATGAGTT
TGATTTTTAA

>GAB2-2007-GAL-DOM2
ATGGCACACGGTGGCATCTATCTGAGGCAGAAGCGTAACTTCTGTCCT
TTAACTGTCTCCACAGTTGCTGTGGTCTTTGTAGTCTTCATGGGTGTA
CTCGTCAATTCGTTGGGTGGAGTCGCTGTCGCAGCAGACAGCGGTGG
TGTTAGGCAGACCCCTTCGGAAACCGGTTCGAGCGGTGGACAGCAAG
AAGCAGTGGGGACCACTGAAGACTATGTCAACTCTTCGGCGATGGGC
GGTGGCCAAGGCGACTCGTTAGCTGAAGATGATACAACCTCCGATGCG
GCGGAGGGCGACGTTGACCCTTTTCCCGCGCTGGCGAATGAGGGGAA
GTCGGAGGCGCGTGGCCCGTCGCTCGAGGAAAGAATCGAAGAACAGG
GCACAAGACGACGTTACTCCTCTGTTCAAGAACCACAAGCGAAGGTGC
CTAGCAAACGAACACAGAAACGCCACAGACTCATTGGTGCTGTGGTGT
TGGCAGTATCTGTGGCAATGCTTACCGCTTTCTTTCTTCGAAGGACTG
GACGACGCTCTCCCCAAGAACCATCTGGGGGTGGTGGTGGAAATGAT
GCAGGCAATAATGCTGGGAACGGTGGGAATGAAGGCAGAGGTGAAGG
AGGCGAGGATGACAGGCGCCCGTTGCACCCGGGAAGTGTGAATGAGT
TTGATTTTTAA

>TgCtCo5
ATGGCACACGGTGGCATCCATCTGAGGCAGAAGCGTAACTTCTGTCCT
TTAACTGTCTCCACAGTTGCTGTGGTCTTTGTAGTCTTCATGGGTGTA
CTCGTCAATTCGTTGGGTGGAGTCGCTGTCGCAGCAGACAGCGATGG
TGTTAAGCAGACCCCTTCGGAAACCGGTTCGAGCGGTGGACAGCAAGA
AGCAGTGGGGACCACTGAAGACTATGTCAACTCTTCGGCGATGGGCG
GTGGCCAAGGCGACTCGTTAGCTGAAGATGATACAACCTCCGATGCGG
CGGAGGGCGACGTTGACCCTTTTCCCGTGCTGGCGAATGAGGGGAAG
TCGGAGGCGCGTGGCCCGTCGCTCGAGGAAAGAATCGAAGAACAGGG
CACAAGACGACGTTACTCCTCTGTTCAAGAACCACAAGCGAAGGTGCC
TAGCAAACGAACACAGAAACGCCACAGACTCATTGGTGCTGTGGTGTT
GGCAGTATCTGTGGCAATGCTTACCGCTTTCTTTCTTCGAAGGACTGG

ACGACGCTCTCCCCCAGAACCATCTGGGGATGATGGTGGAAATGATGC
AGGCAATAATGCTGGGAACGGTGGGAATGAAGGCAGAGGTGAAGGAG
GCGAGGATGACAGGCGCGCGTTGCACCCGGAACGTGTGAATGTGTTT
GATTATTAA

>TgCatJpOk1
GTAGCGTGCTTGTTGGCGACTACCTTTTTTTCTTGGGAGTGTCGGCG
AAATGGCACACGGTGGCATCCATCTGAGGCAGAAGCGTAACTTCTGTC
CTGTAACTGTCTCCACAGTTGCTGTGGTCTTTGTAGTCTTCATGGGTG
TACTCGTCAATTCGTTGGGTGGAGTCGCTGTCGCAGCAGACAGCGGT
GGTGTTAAGCAGACCCCTTCGGAAACCGGTTCGAGCGGTGGACAGCA
AGAAGCAGTGGGGACCACTGAAGACTATGTCAACTCTTCGGCGATGGG
CGGTGGCCAAGGCGACTCGTCAGCTGAAGATGATACAACCTCCGAAGC
GGCGGAGGGCGACGTTGACCCTTTTCCCGTGCTGGCGAATGAGGGGA
AGTCGGAGGCGCGTGGCCCGTCGCTCGAGGAAAGAATCGAAGAACAG
GGCACAAGACGACGTTACTCCTCTGTTCAAGAACCACAAGCGAAGGTG
CCTAGCAAACGAACACAGAAACGCCACAGACTCATTGGTGCTGTGGTG
TTGGCAGTATCTGTGGCAATGCTTACCGCTTTCTTTCTTCGAAGGACT
GGACGACGCTCTCCCCAAGAACCATCTGGGGATGGTGGTGGAAATGA
TGCAGGCAATAATGCTGGGAACGGTGGGAATGAAGGCAGAGGTTACG
GAGGCAGAGGTGAAGGAGGAGCCGAGGATGACAGGCGCCCGTTGCAC
CCGGAACGTGTGAATGTGTTTGATTATTAAAGATGAAAACAGGGGGTC
TATGC

>TgCatJpOk2
TGCTTGTTGGCGACTACCTTTTTTTCTTGGGAGTGTCGGCGAAATGG
CACACGGTGGCATCCATCTGAGGCAGAAGCGTAACTTCTGTCCTGTAA
CTGTCTCCACAGTTGCTGTGGTCTTTGTAGTCTTCATGGGTGTACTCG
TCAATTCGTTGGGTGGAGTCGCTGTCGCAGCAGACAGCGGTGGTGTT
AAGCAGACCCCTTCGGAAACCGGTTCGAGCGGTGGACAGCAAGAAGCA
GTGGGGACCACTGAAGACTATGTCAACTCTTCGGCGATGGGCGGTGG
CCAAGGCGACTCGTCAGCTGAAGATGATACAACCTCCGAAGCGGCGGA
GGGCGACGTTGACCCTTTTCCCGTGCTGGCGAATGAGGGGAAGTCGG
AGGCGCGTGGCCCGTCGCTCGAGGAAAGAATCGAAGAACAGGGCACA
AGACGACGTTACTCCTCTGTTCAAGAACCACAAGCGAAGGTGCCTAGC
AAACGAACACAGAAACGCCACAGACTCATTGGTGCTGTGGTGTTGGCA

GTATCTGTGGCAATGCTTACCGCTTTCTTTCTTCGAAGGACTGGACGA
CGCTCTCCCCAAGAACCATCTGGGGATGGTGGTGGAAATGATGCAGG
CAATAATGCTGGGAACGGTGGGAATGAAGGCAGAGGTTACGGAGGCA
GAGGTGAAGGAGGAGCCTAGGATGACAGGCGCCCGTTGCACCCGGAA
CGTGTGAATGTGTTTGATTATTAA

>TgCatJpOk3
CTTGTTGGCGACTACCTTTTTTTCTTGGGAGTGTCGGCGAAATGGCA
CACGGTGGCATCTATCTGAGGCAGAAGCGTAACTTCTGTCCTTTAACT
GTCTCCACAGTTGCTGTGGTCTTTGTAGTCTTCATGGGTGTACTCGTC
AATTCGTTGGGTGGAGTCGCTGTCGCAGCAGACAGCGGTGGTGTTAG
GCAGACCCCTTCGGAAACCGGTTCGAGCGGTGGACAGCAAGAAGCAG
TGGGGACCACTGAAGACTATGTCAACTCTTCGGCGATGGGCGGTGGC
CAAGGCGACTCGTTAGCTGAAGATGATACAACCTCCGATGCGGCGGAG
GGCGACGTTGACCCTTTTCCCGCGCTGGCGAATGAGGGGAAGTCGGA
GGCGCGTGGCCCGTCGCTCGAGGAAAGAATCGAAGAACAGGGCACAA
GACGACGTTACTCCTCTGTTCAAGAACCACAAGCGAAGGTGCCTAGCA
AACGAACACAGAAACGCCACAGACTCATTGGTGCTGTGGTGTTGGCAG
TATCTGTGGCAATGCTTACCGCTTTCTTTCTTCGAAGGACTGGACGAC
GCTCTCCCCAAGAACCATCTGGGGGTGGTGGTGGAAATGATGCAGGC
AATAATGCTGGGAACGGTGGGAATGAAGGCAGAGGTGAAGGAGGCGA
GGATGACAGGCGCCCGTTGCACCCGGGAAGTGTGAATGAGTTTGATT
TTTAAAGATGAAAACAGGGGGTCTATGCGCCACT

>TgCatJpOk4
GACTACCTTTTTTTCTTGGGAGTGTCGGCGAAATGGCACACGGTGGC
ATCTATCTGAGGCAGAAGCGTAACTTCTGTCCTTTAACTGTCTCCACA
GTTGCTGTGGTCTTTGTAGTCTTCATGGGTGTACTCGTCAATTCGTTG
GGTGGAGTCGCTGTCGCAGCAGACAGCGGTGGTGTTAGGCAGACCCC
TTCGGAAACCGGTTCGAGCGGTGGACAGCAAGAAGCAGTGGGGACCA
CTGAAGACTATGTCAACTCTTCGGCGATGGGCGGTGGCCAAGGCGAC
TCGTTAGCTGAAGATGATACAACCTCCGATGCGGCGGAGGGCGACGTT
GACCCTTTTCCCGCGCTGGCGAATGAGGGGAAGTCGGAGGCGCGTGG
CCCGTCGCTCGAGGAAAGAATCGAAGAACAGGGCACAAGACGACGTTA
CTCCTCTGTTCAAGAACCACAAGCGAAGGTGCCTAGCAAACGAACACA
GAAACGCCACAGACTCATTGGTGCTGTGGTGTTGGCAGTATCTGTGG

CAATGCTTACCGCTTTCTTTCTTCGAAGGACTGGACGACGCTCTCCCC
AAGAACCATCTGGGGGTGGTGGTGGAAATGATGCAGGCAATAATGCT
GGGAACGGTGGGAATGAAGGCAGAGGTGAAGGAGGCGAGGATGACAG
GCGCCCGTTGCACCCGGGAAGTGTGAATGAGTTTGATTTTTAAAGATG
AAAACAGGG

>TgCatJpTy1/k-3
ACAGCAGGAAAACAGCTTCGTGGTGCCACGTAGCGTGCTTGTTGGCG
ACTACCTTTTTTTCTTGGGAGTGTCGGCGAAATGGCACACGGTGGCAT
CTATCTGAGGCAGAAGCGTAACTTCTGTCCTTTAACTGTCTCCACAGT
TGCTGTGGTCTTTGTAGTCTTCATGGGTGTACTCGTCAATTCGTTGG
GTGGAGTCGCTGTCGCAGCAGACAGCGGTGGTGTTAGGCAGACCCCT
TCGGAAACCGGTTCGAGCGGTGGACAGCAAGAAGCAGTGGGGACCAC
TGAAGACTATGTCAACTCTTCGGCGATGGGCGGTGGCCAAGGCGACT
CGTTAGCTGAAGATGATACAACCTCCGATGCGGCGGAGGGCGACGTT
GACCCTTTTCCCGCGCTGGCGAATGAGGGGAAGTCGGAGGCGCGTGG
CCCGTCGCTCGAGGAAAGAATCGAAGAACAGGGCACAAGACGACGTTA
CTCCTCTGTTCAAGAACCACAAGCGAAGGTGCCTAGCAAACGAACACA
GAAACGCCACAGACTCATTGGTGCTGTGGTGTTGGCAGTATCTGTGG
CAATGCTTACCGCTTTCTTTCTTCGAAGGACTGGACGACGCTCTCCCC
AAGAACCATCTGGGGGTGGTGGTGGAAATGATGCAGGCAATAATGCT
GGGAACGGTGGGAATGAAGGCAGAGGTGAAGGAGGCGAGGATGACAG
GCGCCCGTTGCACCCGGGAAGTGTGAATGAGTTTGATTTTTAAAGATG
AAAACAGGGGGTCTATGCGCCACTGGGGCACTCTGTGTCTTGTAGTC
GATGC

>TgCatJpGi1/TaJ
GTGGTGCCACGTAGCGTGCTTGTTGGCGACTACCTTTTTTTCTTGGG
AGTGTCGGCGAAATGGCACACGGTGGCATCCATCTGAGGCAGAAGCG
TAACTTCTGTCCTTTAACTGTCTCCACAGTTGCTGTGGTCTTTGTAGT
TTTCATGGGTGTACTCGTCAATTCGTTGGGTGGAGTCGCTGTCGCAG
CAGACAGCGATGGTGTTAAGCAGACCCCTTCGGAAACCGGTTCGAGCG
GTGGACAGCAAGAAGCAGTGGGGACCACTGAAGACTATGTCAACTCTT
CGGCGATGGGCGGTGGCCAAGGCGACTCGTTAGCTGAAGATGATACA
ACCTCCGATGCGGCGGAGGGCGACGTTGACCCTTTTCCCGTGCTGGC
GAATGAGGGGAAGTCGGAGGCGCGTGGCCCGTCGCTCGAGGAAAGAA

TCGAAGAACAGGGCACAAGACGACGTTACTCCTCTGTTCAAGAACCAC
AAGCGAAGGTGCCTAGCAAACGAACACAGAAACGCCACAGACTCATTG
GTGCTGTGGTGTTGGCAGTATCTGTGGCAATGCTTACCGCTTTCTTT
CTTCGAAGGACTGGACGACGCTCTCCCCCAGAACCATCTGGGGATGG
TGGTGGAAATGATGCAGGCAATAATGCTGGGAACCGTGGGAATGAAG
GCAGAGGTTACGGAGGCAGAGGTGAAGGAGGAGGCGAGGATGACAGG
CGCGCGTTGCACCCGGAACGTGTGAATGTGTTTGATTATTAAAGATGA
AAACAGGGGGTCTATGCGCCACTGGGGCACTCTGT

>TgGoatJpOk1
AATGGCACACGGTGGCATCCATCTGAGGCAGAAGCGTAACTTCTGTCC
TGTAACTGTCTCCACAGTTGCTGTGGTCTTTGTAGTCTTCATGGGTGT
ACTCGTCAATTCGTTGGGTGGAGTCGCTGTCGCAGCAGACAGCGGTG
GTGTTAAGCAGACCCCTTCGGAAACCGGTTCGAGCGGTGGACAGCAA
GAAGCAGTGGGGACCACTGAAGACTATGTCAACTCTTCGGCGATGGG
CGGTGGCCAAGGCGACTCGTCAGCTGAAGATGATACAACCTCCGAAGC
GGCGGAGGGCGACGTTGACCCTTTTCCCGTGCTGGCGAATGAGGGGA
AGTCGGAGGCGCGTGGCCCGTCGCTCGAGGAAAGAATCGAAGAACAG
GGCACAAGACGACGTTACTCCTCTGTTCAAGAACCACAAGCGAAGGTG
CCTAGCAAACGAACACAGAAACGCCACAGACTCATTGGTGCTGTGGTG
TTGGCAGTATCTGTGGCAATGCTTACCGCTTTCTTTCTTCGAAGGACT
GGACGACGCTCTCCCCAAGAACCATCTGGGGATGGTGGTGGAAATGA
TGCAGGCAATAATGCTGGGAACGGTGGGAATGAAGGCAGAGGTTACG
GAGGCAGAGGTGAAGGAGGAGCCGAGGATGACAGGCGCCCGTTGCAC
CCGGAACGTGTGAATGTGTTTGATTATTAAAGATGAAAACAGGGGGGT
CTATGCGCCACTGGG

>TgGoatJpOk2
TCTTGGGAGTGTCGGCGAAATGGCACACGGTGGCATCCATCTGAGGC
AGAAGCGTAACTTCTGTCCTGTAACTGTCTCCACAGTTGCTGTGGTCT
TTGTAGTCTTCATGGGTGTACTCGTCAATTCGTTGGGTGGAGTCGCT
GTCGCAGCAGACAGCGGTGGTGTTAAGCAGACCCCTTCGGAAACCGG
TTCGAGCGGTGGACAGCAAGAAGCAGTGGGGACCACTGAAGACTATGT
CAACTCTTCGGCGATGGGCGGTGGCCAAGGCGACTCGTCAGCTGAAG
ATGATACAACCTCCGAAGCGGCGGAGGGCGACGTTGACCCTTTTCCCG
TGCTGGCGAATGAGGGGAAGTCGGAGGCGCGTGGCCCGTCGCTCGA

GGAAAGAATCGAAGAACAGGGCACAAGACGACGTTACTCCTCTGTTCA
AGAACCACAAGCGAAGGTGCCTAGCAAACGAACACAGAAACGCCACAG
ACTCATTGGTGCTGTGGTGTTGGCAGTATCTGTGGCAATGCTTACCGC
TTTCTTTCTTCGAAGGACTGGACGACGCTCTCCCCAAGAACCATCTGG
GGATGGTGGTGGAAATGATGCAGGCAATAATGCTGGGAACGGTGGGA
ATGAAGGCAGAGGTTACGGAGGCAGAGGTGAAGGAGGAGCCGAGGAT
GACAGGCGCCCGTTGCACCCGGAACGTGTGAATGTGTTTGATTATTAA
A

>TgGoatJpOk3
GAAATGGCACACGGTGGCATCCATCTGAGGCAGAAGCGTAACTTCTGT
CCTGTAACTGTCTCCACAGTTGCTGTGGTCTTTGTAGTCTTCATGGGT
GTACTCGTCAATTCGTTGGGTGGAGTCGCTGTCGCAGCAGACAGCGG
TGGTGTTAAGCAGACCCCTTCGGAAACCGGTTCGAGCGGTGGACAGC
AAGAAGCAGTGGGGACCACTGAAGACTATGTCAACTCTTCGGCGATGG
GCGGTGGCCAAGGCGACTCGTCAGCTGAAGATGATACAACCTCCGAAG
CGGCGGAGGGCGACGTTGACCCTTTTCCCGTGCTGGCGAATGAGGGG
AAGTCGGAGGCGCGTGGCCCGTCGCTCGAGGAAAGAATCGAAGAACA
GGGCACAAGACGACGTTACTCCTCTGTTCAAGAACCACAAGCGAAGGT
GCCTAGCAAACGAACACAGAAACGCCACAGACTCATTGGTGCTGTGGT
GTTGGCAGTATCTGTGGCAATGCTTACCGCTTTCTTTCTTCGAAGGAC
TGGACGACGCTCTCCCCAAGAACCATCTGGGGATGGTGGTGGAAATG
ATGCAGGCAATAATGCTGGGAACGGTGGGAATGAAGGCAGAGGTTAC
GGAGGCAGAGGTGAAGGAGGAGCCGAGGATGACAGGCGCCCGTTGCA
CCCGGAACGTGTGAATGTGTTTGATTATTAAAGATGAAAACAGGGGGT
CTATGCGCCACTGGGGCAC

>TgGoatJpOk4
GTTGGCGACTACCTTTTTTTCTTGGGAGTGTCGGCGAAATGGCACAC
GGTGGCATCCATCTGAGGCAGAAGCGTAACTTCTGTCCTGTAACTGTC
TCCACAGTTGCTGTGGTCTTTGTAGTCTTCATGGGTGTACTCGTCAAT
TCGTTGGGTGGAGTCGCTGTCGCAGCAGACAGCGGTGGTGTTAAGCA
GACCCCTTCGGAAACCGGTTCGAGCGGTGGACAGCAAGAAGCAGTGG
GGACCACTGAAGACTATGTCAACTCTTCGGCGATGGGCGGTGGCCAA
GGCGACTCGTCAGCTGAAGATGATACAACCTCCGAAGCGGCGGAGGG
CGACGTTGACCCTTTTCCCGTGCTGGCGAATGAGGGGAAGTCGGAGG

CGCGTGGCCCGTCGCTCGAGGAAAGAATCGAAGAACAGGGCACAAGA
CGACGTTACTCCTCTGTTCAAGAACCACAAGCGAAGGTGCCTAGCAAA
CGAACACAGAAACGCCACAGACTCATTGGTGCTGTGGTGTTGGCAGTA
TCTGTGGCAATGCTTACCGCTTTCTTTCTTCGAAGGACTGGACGACGC
TCTCCCCAAGAACCATCTGGGGATGGTGGTGGAAATGATGCAGGCAAT
AATGCTGGGAACGGTGGGAATGAAGGCAGAGGTTACGGAGGCAGAGG
TGAAGGAGGAGCCGAGGATGACAGGCGCCCGTTGCACCCGGAACGTG
TGAATGTGTTTGATTATTAAAGATGAAAACAGGGGGTCTATGCGC

>TgGoatJpOk5
TGTTGGGCGACTACCTTTTTTTCTTGGGAGTGTCGGCGAAATGGCAC
ACGGTGGCATCCATCTGAGGCAGAAGCGTAACTTCTGTCCTGTAACTG
TCTCCACAGTTGCTGTGGTCTTTGTAGTCTTCATGGGTGTACTCGTCA
ATTCGTTGGGTGGAGTCGCTGTCGCAGCAGACAGCGGTGGTGTTAAG
CAGACCCCTTCGGAAACCGGTTCGAGCGGTGGACAGCAAGAAGCAGT
GGGGACCACTGAAGACTATGTCAACTCTTCGGCGATGGGCGGTGGCC
AAGGCGACTCGTCAGCTGAAGATGATACAACCTCCGAAGCGGCGGAGG
GCGACGTTGACCCTTTTCCCGTGCTGGCGAATGAGGGGAAGTCGGAG
GCGCGTGGCCCGTCGCTCGAGGAAAGAATCGAAGAACAGGGCACAAG
ACGACGTTACTCCTCTGTTCAAGAACCACAAGCGAAGGTGCCTAGCAA
ACGAACACAGAAACGCCACAGACTCATTGGTGCTGTGGTGTTGGCAGT
ATCTGTGGCAATGCTTACCGCTTTCTTTCTTCGAAGGACTGGACGACG
CTCTCCCCAAGAACCATCTGGGGATGGTGGTGGAAATGATGCAGGCA
ATAATGCTGGGAACGGTGGGAATGAAGGCAGAGGTTACGGAGGCAGA
GGTGAAGGAGGAGCCGAGGATGACAGGCGCCCGTTGCACCCGGAACG
TGTGAATGTGTTTGATTATTAAAGATGAAAACAGGGGGGTCTATGCGC
CACTGGGGGC

>TgGoatJpOk6
TTACAGCAGGAAAACAGCTTCGTGGTGCCACGTAGCGTGCTTGTTGGC
GACTACCTTTTTTTCTTGGGAGTGTCGGCGAAATGGCACACGGTGGC
ATCTATCTGAGGCAGAAGCGTAACTTCTGTCCTTTAACTGTCTCCACA
GTTGCTGTGGTCTTTGTAGTCTTCATGGGTGTACTCGTCAATTCGTTG
GGTGGAGTCGCTGTCGCAGCAGACAGCGGTGGTGTTAGGCAGACCCC
TTCGGAAACCGGTTCGAGCGGTGGACAGCAAGAAGCAGTGGGGACCA
CTGAAGACTATGTCAACTCTTCGGCGATGGGCGGTGGCCAAGGCGAC

TCGTTAGCTGAAGATGATACAACCTCCGATGCGGCGGAGGGCGACGTT
GACCCTTTTCCCGCGCTGGCGAATGAGGGGAAGTCGGAGGCGCGTGG
CCCGTCGCTCGAGGAAAGAATCGAAGAACAGGGCACAAGACGACGTTA
CTCCTCTGTTCAAGAACCACAAGCGAAGGTGCCTAGCAAACGAACACA
GAAACGCCACAGACTCATTGGTGCTGTGGTGTTGGCAGTATCTGTGG
CAATGCTTACCGCTTTCTTTCTTCGAAGGACTGGACGACGCTCTCCCC
AAGAACCATCTGGGGGTGGTGGTGGAAATGATGCAGGCAATAATGCT
GGGAACGGTGGGAATGAAGGCAGAGGTGAAGGAGGCGAGGATGACAG
GCGCCCGTTGCACCCGGGAAGTGTGAATGAGTTTGATTTTTAAAGATG
AAAACCGGGGGTCTATGCTTTTCGGGGGCACTCTGTGTCCTGTATTC
TATGCAGTCCCCGCTCGTGTCGTCCGTCTTTGAGCANCCAGTGCTTCC
TCCATTACCGCTCTAAATAGTGCTTAATTACGTACCATCCCTGCCTGAA
TTACGAGTCCGTTAATGAGGTGACTCATGAACGGAAGATTCATGCGTT
TTGATACCTG

>TgGoatJpOk7
TACAGCAGGAAAACAGCTTCGTGGTgCCACGTAGCGTGCTTGTTGGCG
ACTACCTTTTTTTCTTGGGAGTGTCGGCGAAATGGCACACGGTGGCAT
CTATCTGAGGCAGAAGCGTAACTTCTGTCCTTTAACTGTCTCCACAGT
TGCTGTGGTCTTTGTAGTCTTCATGGGTGTACTCGTCAATTCGTTGG
GTGGAGTCGCTGTCGCAGCAGACAGCGGTGGTGTTAGGCAGACCCCT
TCGGAAACCGGTTCGAGCGGTGGACAGCAAGAAGCAGTGGGGACCAC
TGAAGACTATGTCAACTCTTCGGCGATGGGCGGTGGCCAAGGCGACT
CGTTAGCTGAAGATGATACAACCTCCGATGCGGCGGAGGGCGACGTT
GACCCTTTTCCCGCGCTGGCGAATGAGGGGAAGTCGGAGGCGCGTGG
CCCGTCGCTCGAGGAAAGAATCGAAGAACAGGGCACAAGACGACGTTA
CTCCTCTGTTCAAGAACCACAAGCGAAGGTGCCTAGCAAACGAACACA
GAAACGCCACAGACTCATTGGTGCTGTGGTGTTGGCAGTATCTGTGG
CAATGCTTACCGCTTTCTTTCTTCGAAGGACTGGACGACGCTCTCCCC
AAGAACCATCTGGGGGTGGTGGTGGAAATGATGCAGGCAATAATGCT
GGGAACGGTGGGAATGAAGGCAGAGGTGAAGGAGGCGAGGATGACAG
GCGCCCGTTGCACCCGGGAAGTGTGAATGAGTTTGATTTTTAAAGATG
AAAACAGGGGGTCTATGCGCCACTGGGGCACTCTGTGTCTTGTA

>TgGoatJpOk8
TACAGCAGGAAAACAGCTTCGTGGTGCCACGTAGCGTGCTTGTTGGC

GACTACCTTTTTTTCTTGGGAGTGTCGGCGAAATGGCACACGGTGGC
ATCTATCTGAGGCAGAAGCGTAACTTCTGTCCTTTAACTGTCTCCACA
GTTGCTGTGGTCTTTGTAGTCTTCATGGGTGTACTCGTCAATTCGTTG
GGTGGAGTCGCTGTCGCAGCAGACAGCGGTGGTGTTAGGCAGACCCC
TTCGGAAACCGGTTCGAGCGGTGGACAGCAAGAAGCAGTGGGGACCA
CTGAAGACTATGTCAACTCTTCGGCGATGGGCGGTGGCCAAGGCGAC
TCGTTAGCTGAAGATGATACAACCTCCGATGCGGCGGAGGGCGACGTT
GACCCTTTTCCCGCGCTGGCGAATGAGGGGAAGTCGGAGGCGCGTGG
CCCGTCGCTCGAGGAAAGAATCGAAGAACAGGGCACAAGACGACGTTA
CTCCTCTGTTCAAGAACCACAAGCGAAGGTGCCTAGCAAACGAACACA
GAAACGCCACAGACTCATTGGTGCTGTGGTGTTGGCAGTATCTGTGG
CAATGCTTACCGCTTTCTTTCTTCGAAGGACTGGACGACGCTCTCCCC
AAGAACCATCTGGGGGTGGTGGTGGAAATGATGCAGGCAATAATGCT
GGGAACGGTGGGAATGAAGGCAGAGGTGAAGGAGGCGAGGATGACAG
GCGCCCGTTGCACCCGGGAAGTGTGAATGAGTTTGATTTTTAAAGATG
AAAACAGGGGGTCTATGCGCCACTGGGGCACTCTGTGT

>TgGoatJpOk9
GCAGGAAAACAGCTTCGTGGTGCCACGTAGCGTGCTTGTTGGCGACT
ACCTTTTTTTCTTGGGAGTGTCGGCGAAATGGCACACGGTGGCATCT
ATCTGAGGCAGAAGCGTAACTTCTGTCCTTTAACTGTCTCCACAGTTG
CTGTGGTCTTTGTAGTCTTCATGGGTGTACTCGTCAATTCGTTGGGT
GGAGTCGCTGTCGCAGCAGACAGCGGTGGTGTTAGGCAGACCCCTTC
GGAAACCGGTTCGAGCGGTGGACAGCAAGAAGCAGTGGGGACCACTG
AAGACTATGTCAACTCTTCGGCGATGGGCGGTGGCCAAGGCGACTCG
TTAGCTGAAGATGATACAACCTCCGATGCGGCGGAGGGCGACGTTGAC
CCTTTTCCCGCGCTGGCGAATGAGGGGAAGTCGGAGGCGCGTGGCCC
GTCGCTCGAGGAAAGAATCGAAGAACAGGGCACAAGACGACGTTACTC
CTCTGTTCAAGAACCACAAGCGAAGGTGCCTAGCAAACGAACACAGAA
ACGCCACAGACTCATTGGTGCTGTGGTGTTGGCAGTATCTGTGGCAAT
GCTTACCGCTTTCTTTCTTCGAAGGACTGGACGACGCTCTCCCCAAGA
ACCATCTGGGGGTGGTGGTGGAAATGATGCAGGCAATAATGCTGGGA
ACGGTGGGAATGAAGGCAGAGGTGAAGGAGGCGAGGATGACAGGCGC
CCGTTGCACCCGGGAAGTGTGAATGAGTTTGATTTTTAAAGATGAAAA
CAGGGGGTCTATGCGCCACTGGGGCACTCTGTGT

>TgGoatJpOk10
CACGTAGCGTGCTTGTTGGCGACTACCNTTTTTTCTTGGGAGTGTCG
GCGAAATGGCACACGGTGGCATCTATCTGAGGCAGAAGCGTAACTTCT
GTCCTTTAACTGTCTCCACAGTTGCTGTGGTCTTTGTAGTCTTCATGG
GTGTACTCGTCAATTCGTTGGGTGGAGTCGCTGTCGCAGCAGACAGC
GGTGGTGTTAGGCAGACCCCTTCGGAAACCGGTTCGAGCGGTGGACA
GCAAGAAGCAGTGGGGACCACTGAAGACTATGTCAACTCTTCGGCGAT
GGGCGGTGGCCAAGGCGACTCGTTAGCTGAAGATGATACAACCTCCG
ATGCGGCGGAGGGCGACGTTGACCCTTTTCCCGCGCTGGCGAATGAG
GGGAAGTCGGAGGCGCGTGGCCCGTCGCTCGAGGAAAGAATCGAAGA
ACAGGGCACAAGACGACGTTACTCCTCTGTTCAAGAACCACAAGCGAA
GGTGCCTAGCAAACGAACACAGAAACGCCACAGACTCATTGGTGCTGT
GGTGTTGGCAGTATCTGTGGCAATGCTTACCGCTTTCTTTCTTCGAAG
GACTGGACGACGCTCTCCCCAAGAACCATCTGGGGGGTGGTGGTNGG
AAATGATGCAGGCAATAATGCTGGGAACGGTGGGAATGAAGGCAGAG
GTGAAGGAGGCGAGGATGACAGGCGCCCCGTTGCACCCGGGAAGTGT
GAATGAGTTTGATTTTTAAAG

>TgGoatJpOk11
GCAGGAAAACAGCTTCGTGGTGCCACGTAGCGTGCTTGTTGGCGACT
ACCTTTTTTTCTTGGGAGTGTCGGCGAAATGGCACACGGTGGCATCC
ATCTGAGGCAGAAGCGTAACTTCTGTCCTTTAACTGTCTCCACAGTTG
CTGTGGTCTTTGTAGTTTTCATGGGTGTACTCGTCAATTCGTTGGGT
GGAGTCGCTGTCGCAGCAGACAGCGATGGTGTTAAGCAGACCCCTTC
GGAAACCGGTTCGAGCGGTGGACAGCAAGAAGCAGTGGGGACCACTG
AAGACTATGTCAACTCTTCGGCGATGGGCGGTGGCCAAGGCGACTCG
TTAGCTGAaGATGATACAACCTCCGATGCGGCGGAgGGCGACGTTGAC
CCTTTTCCCGTGCTGGCGAATGAGGGGAAGTCGGAGGCGCGTGGCCC
GTCGCTCGAGGAAAGAATCGAAGAACAGGGCACAAGACGACGTTACTC
CTCTGTTCAAGAACCACAAGCGAAGGTGCCTAGCAAACGAACACAGAA
ACGCCACAGACTCATTGGTGCTGTGGTGTTGGCAGTATCTGTGGCAAT
GCTTACCGCTTTCTTTCTTCGAAGGACTGGACGACGCTCTCCCCCAGA
ACCATCTGGGGATGGTGGTGGAAATGATGCAGGCAATAATGCTGGGA
ACCGTGGGAATGAAGGCAGAGGTTACGGAGGCAGAGGTGAAGGAGGA
GGCGAGGATGACAGGCGCGCGTTGCACCCGGAACGTGTGAATGTGTT
TGATTATTAAAGATGAAAACAGGGGGTCTATGCGCCACTGGGGCACTC

TGTGTCTTG

Dense granule protein 7 (GRA7)

>GT1
ATGGCCCGACACGCAATTTTTTCCGCGCTTTGTGTTTTAGGCCTGGT
GGCGGCGGCTTTGCCCCAGTTCGCTACCGCGGCCACCGCGTCAGATG
ACGAACTGATGAGTCGAATCCGAAATTCTGACTTTTTCGATGGTCAAG
CACCCGTTGACAGTCTCAGACCGACGAACGCCGGTGTCGACTCGAAAG
GGACCGACGATCACCTCACCACCAGCATGGATAAGGCATCTGTAGAGA
GTCAGCTTCCGAGAAGAGAGCCATTGGAGACGGAGCCAGATGAACAAG
AAGAAGTTCATTTCAGGAAGCGAGGCGTCCGTTCCGACGCTGAAGTGA
CTGACGACAACATCTACGAGGAGCACACTGATCGTAAGGTGGTTCCGA
GGAAGTCGGAGGGCAAGCGAAGCTTCAAAGACTTGCTGAAGAAGCTC
GCGCTGCCGGCTGTTGGTATGGGTGCATCGTATTTTGCCGCTGATAG
ACTTGTGCCGGAACTAACAGAGGAGCAACAGAGAGGCGACGAACCCCT
AACCACCGGCCAGAATGTGGGCACTGTGTTAGGCTTCGCAGCGCTTG
CTGCTGCCGCAGCGTTCCTTGGCATGGGTCTCACGAGGACGTACCGA
CATTTTTCCCCACGCAAAAACAGATCACGGCAGCCTGCACTCGAGCAA
GAGGTGCCTGAATCAGGCGAAGATGGGGAGGATGCCCGCCATGTCCC
GAACGCGCCTGAGAGGCAGACAGACACAGAAGAGTGA

>ME49
ATGGCCCGACACGCAATTTTTTTCGCGCTTTGTGTTTTAGGCCTGGT
GGCGGCGGCTTTGCCCCAGTTCGCTACCGCGGCCACCGCGTCAGATG
ACGAACTGATGAGTCGAATCCGAAATTCTGACTTTTTCGATGGTCAAG
CACCCGTTGACAGTCTCAGACCGACGAACGCCGGTGTCGACTCGAAAG
GGACCGACGATCACCTCACCACCAGCATGGATAAGGCATCTGTAGAGA
GTCAGCTTCCGAGAAGAGAGCCATTGGAGACGGAGCCAGATGAACAAG
AAGAAGTTCATTTCAGGAAGCGAGGCGTCCGTTCCGACGCTGAAGTGA
CTGACGACAACATCTACGAGGAGCACACTGATCGTAAAGTGGTTCCGA
GGAAGTCGGAGGGCAAGCGAAGCTTCAAAGACTTGCTGAAGAAGCTC
GCGCTGCCGGCTGTTGGTATGGGTGCATCGTATTTTGCCGCTGATAG
AATTCTGCCGGAACTAACAGAGCAGCAACAGACAGGCGAAGAACCCCT
AACCACCGGCCAGAATGTGAGCACTGTGTTAGGCTTCGCAGCGCTTGC
TGCTGCCGCAGCGTTCCTTGGCATGGGTCTCACGAGGACGTACCGAC
ATTTTTCCCCACGCAAAAACAGATCACGGCAGCCTGCACTCGAGCAAG
AGGTGCCTGAATCAGGCAAAGATGGGGAGGATGCCCGCCAGTAG

>VEG
ATGGCCCGACACGCAATTTTTTTCGCGCTTTGTGTTTTAGGCCTGGT
GGCGGCGGCTTTGCCCCAGTTCGCTACCGCGGCCACCGCGTCGGATG
ACGAACTGATGAGTCGAATCCGAAATTCTGACTTTTTCGATGGTCAAG
CACCCGTTGACAGTCTCAGACCGACGAACGCCGGTGTCGACTCGAAAG
GGACCGACGATCACCTCACCACCAGCATGGATAAGGCATCTGTAGAGA
GTCAGCTTCCGAGAAGAGAGCCATTGGAGACGGAGCCAGATGAACAAG
AAGAAGTTCATTTCAGGAAGCGAGGCGTCGGTTCCGACGCTGAAGTG
ACTGACGACCACATCTACGAGGAGAACACTGATCGTAAAGTGGTTCCG
AGGAAGTCGGAGGGCAAGCGAAGCTTCAAAGACTTGCTGAAGAAGCT
CGCGCTGCCGGCTGTTGGTATGGGTGCATCGTATTTTGCCGCTGATA
GAATTCTGCCGGAACTAACAGAGCAGCAACAGACAGGCGACGAACCCC
TATCCACCGGCCAGAATGTGAGCACTGTGATAGGCTTCGCAGCGCTTG
CTGCTGCCGTAGCGTTCCTTGGCCTGGGTATCAAGAGGACGTACCGAC
ATTTTTCCCCACGCAAAAACAGATCACGGCAGCCTGCACCCGAGCACG
AGGTGCCTGAATCAGGCGAAGATAGGGAGGATGCCCGCCATGTCCCG
AACGCGCCTGAGAGGCAGAGAGACACAGAAGAGTGA

>MAS
ATGGCCCGACACGCAATTTTTTTCGCGCTTTGTGTTTTAGGCCTGGT
GGCGGCGGCTTTGCCCCAGTTCGCTACCGCGGCCACCGCGTCAGATG
ACGAACTGATGAGTCGAATCCGAAATTCTGACTTTTTCGATGGTCAAG
CACCCGTTGACAGTCTCAGACCGACGAACGCCGGTGTCGACTCGAAAG
GGACCGACGATCACCTCACCACCAGCATGGATAAGGCATCTGTAGAGA
GTCAGCTTCCGAGAAGAGAGCCATTGGAGACGGAGCCAGATGAACAAG
AAGAAGTTCATTTCAGGAAGCGAGGCGTCGGTTCCGACGCTGAAGTG
ACTGACGACCACATCTACGAGGAGCACACTGATCGTAAAGTGGTTCCG
AGGAAGTCGGAGGGCAAGCGAAGCTTCAAAGACTTGCTGAAGAAGCT
CGCGCTGCCGGCTGTTGGTATGGGTGCATCGTATTTTGCCGCTGATA
GAATTCTGCCGGAACTAACAGAGCAGCAACAGACAGGCGACGAACCCC
TATCCACCGGCCAGAATGTGAGCACTGTGATAGGCTTCGCAGCGCTTG
CTGCTGCCGTAGCGTTCCTTGGCCTGGGTATCAAGAGGACGTACCGAC
ATTTTTCCCCACGCAAAAACAGATCACGGCAGCCTGCACCCGAGCACG
AGGTGCCTGAATCAGGCGAAGATAGGGAGGATGCCCGCCAGTAG

>RUB
ATGGCCCGACACGCAATTTTTTTCGCGCTTTGTGTTTTAGGCCTGGT
GGCGGCGGCTTTGCCCCAGTTCGCTACCGCGGCCACCGCGTCAGATG
ACGAACTGATGAGTCGAACCCGAAATTCTGACTTTTTCGATGGTCAAG
CACCCGTTGACAGTCTCAGACCGACGAACGCCGGTGTCGACTCGAAAG
GGACCGACGATCACCTCACCACCAGCATGGATAAGGCATCTGTAGAGA
GTCAGCTTCCGAGAAGAGAGCCATTGGAGACGGAGCCAGATGAACAAG
AAGAAGTTCATTTCAGGAAGCGAGGCGTCCGTTCCGACGCTGAAGTGA
CTGACGACAACATCTACGAGGAGCACACTGATCGTAAAGTGGTTCCGA
GGAAGTCGGAGGGCAAGCGAAGCTTCAAAGACTTGCTGAAGAAGCTC
GCGCTGCCGGCTGTTGGTATGGGTGCATCGTATTTTGCCGCTGATAG
AATTCTGCCGGAACTAACAGAGCAGCAACAGGCAGGCGAAGAACCCCT
AACCACCAGCCAGAATGTGAACACTGTGTTAGGCTTCGCAGCGCTTGC
TGCTGCCGCAGCGTTCCTTGGCATGGGTCTCACGAGGACGTACCGAC
ATTTTTCCCCACGCAAAAACAGATCACGGCAGCCTGCACTCGAGCAAG
AGGTGCCTGAATCAGGCGAAGATGGGGAGGATGCCCGCCAGTAG

>FOU
ATGGCCCGACACGCAATTTTTTTCCGCGCTTTGTGTTTTAGGCCTGGT
GGCGGCGGCTTTGCCCCAGTTCGCTACCGCGGCCACCGCGTCAGATG
ACGAACTGATGAGTCGAATCCGAAATTCTGACTTTTTCGATGGTCAAG
CACCCGTTGACAGTCTCAGACCGACGAACGCCGGTGTCGACTCGAAAG
GGACCGACGATCACCTCACCACCAGCATGGATAAGGCATCTGTAGAGA
GTCAGCTTCCGAGAAGAGAGCCATTGGAGACGGAGCCAGATGAACAAG
AAGAAGTTCATTTCAGGAAGCGAGGCGTCCGTTCCGACGCTGAAGTGA
CTGACGACAACATCTACGAGGAGCACACTGATCGTAAGGTGGTTCCGA
GGAAGTCGGAGGGCAAGCGAAGCTTCAAAGACTTGCTGAAGAAGCTC
GCGCTGCCGGCTGTTGGTATGGGTGCATCGTATTTTGCCGCTGATAG
ACTTGTGCCGGAACTAACAGAGGAGCAACAGAGAGGCGACGAACCCCT
AACCACCGGCCAGAATGTGGGCACTGTGTTAGGCTTCGCAGCGCTTG
CTGCTGCCGCAGCGTTCCTTGGCATGGGTCTCACGAGGACGTACCGA
CATTTTTCCCCACGCAAAAACAGATCACGGCAGCCTGCACTCGAGCAA
GAGGTGCCTGAATCAGGCGAAGATGGGGAGGATGCCCGCCATGTCCC
GAACGCGCCTGAGAGGCAGACAGACACAGAAGAGTGA

>CAST

ATGGCCCGACACGCAATTTTTTTCGCGCTTTGTGTTTTAGGCCTGGT
GGCGGCGGCTTTGCCCCAGTTCGCTACCGCGGCCACCGCGTCAGATG
ACGAACTGATGAGTCGAATCCAAAATTCTGACTTTTTCGATGGTCAAG
CACCCGTTGACAGTCTCAGACCGACGAACGCCGGTGTCGACTCGAAAG
GGACCGACGATCACCTCACCACCAGCATGGATAAGGCATCTGTAGAGA
GTCAGCTTCCGAGAAGAGAGCCATTGGAGACGGAACCAGATGAACAAG
AAGAAGTTCATTTCAGGAAGCGAGGCGTCGGTTCCGACGCTGAAGTG
ACTGACGACCACATCTACGAGGAGCACACTGATCGCAAAGTGGTTCCG
AGGATGTCGGAGGGCAAGCGAAGCTTCAAAGACTTGCTGAAGAAGCT
CGCGCTGCCGGCTGTTGGTATGGGTGCATCGTATTTTGCCGCTGATA
GAATTATGCCGGAACTAACAGAGCAGCAACAGGCAGGCGACGAACCCC
TAACCACCGGCCAGAATGTGAACACTGTGTTAGGCTTCGCAGCGCTTG
CTGCTGCCGTAGCGTTCCTTGGCCTGGGTATCAAGAGGACGTACCGAC
ATTTTTCCCCACGCAAAAACAGATCACGGCAGCCTGCACCCGAGCACG
AGGTGCCTGAATCAGGCGAAGATAGGGAGGATGCCCGCCATGTCCCG
AACGCGCCTGAGAGGCAGACAGACACAGAAGAGTGA

>TgCatBr5
ATGGCCCGACACGCAATTTTTTTCGCGCTTTGTGTTTTAGGCCTGGT
GGCGGCGGCTTTGCCCCAGTTCGCTACCGCGGCCACCGCGTCAGATG
ACGAACTGATGAGTCGAATCCGAAATTCTGACTTTTTCGATGGTCAAG
CACCCGTTGACAGTCTCAGACCGACGAACGCCGGTGTCGACTCGAAAG
GGACCGACGATCACCTCACCACCAGCATGGATAAGGCATCTGTAGAGA
GTCAGCTTCCGAGAAGAGAGCCATTGGAGACGGAGCCAGATGAACAAG
AAGAAGTTCATTTCAGGAAGCGAGGCGTCGGTTCCGACGCTGAAGTG
ACTGACGACCACATCTACGAGGAGCACACTGATCGTAAAGTGGTTCCG
AGGAAGTCGGAGGGCAAGCGAAGCTTCAAAGACTTGCTGAAGAAGCT
CGCGCTGCCGGCTGTTGGTATGGGTGCATCGTATTTTGCCGCTGATA
GAATTCTGCCGGAACTAACAGAGCAGCAACAGACAGGCGACGAACCCC
TATCCACCGGCCAGAATGTGAGCACTGTGATAGGCTTCGCAGCGCTTG
CTGCTGCCGTAGCGTTCCTTGGCCTGGGTATCAAGAGGACGTACCGAC
ATTTTTCCCCACGCAAAAACAGATCACGGCAGCCTGCACCCGAGCACG
AGGTGCCTGAATCAGGCGAAGATAGGGAGGATGCCCGCCAGTAG

>p89
ATGGCCCGACACGCAATTTTTTCCGCGCTTTGTGTTTTAGGCCTGGT

GGCGGCGGCTTTGCCCCAGTTCGCTACCGCGGCCACCGCGTCAGATG
ACGAACTGATGAGTCGAATCCGAAATTCTGACTTTTTCGATGGTCAAG
CACCCGTTGACAGTCTCAGACCGACGAACGCCGGTGTCGACTCGAAAG
GGACCGACGATCACCTCACCACCAGCATGGATAAGGCATCTGTAGAGA
GTCAGCTTCCGAGAAGAGAGCCATTGGAGACGGAGCCAGATGAACAAG
AAGAAGTTCATTTCAGGAAGCGAGGCGTCGGTTCCGACGCTGAAGTG
ACTGACGACCACATCTACGAGGAGAACACTGATCGTAAAGTGGTTCCG
AGGAAGTCGGAGGGCAAGCGAAGCTTCAAAGACTTGCTGAAGAAGCT
CGCGCTGCCGGCTGTTGGTATGGGTGCATCGTATTTTGCCGCTGATA
GAATTCTGCCGGAACTAACAGAGCAGCAACAGACAGGCGACGAACCCC
TATCCACCGGCCAGAATGTGAGCACTGTGATAGGCTTCGCAGCGCTTG
CTGCTGCCGTAGCGTTCCTTGGCCTGGGTATCAAGAGGACGTACCGAC
ATTTTTCCCCACGCAAAAACAGATCACGGCAGCCTGCACCCGAGCACG
AGGTGCCTGAATCAGGCGAAGATAGGGAGGATGCCCGCCAGTAG

>VAND
ATGGCCCGACACGCAATTTTTTTCGCGCTTTGTGTTTTAGGCCTGGT
GGCGGCGGCTTTGCCCCAGTTCGCTACCGCGGCCACCGCGTCAGATG
ACGAACTGATGAGTCGAACCCGAAATTCTGACTTTTTCGATGGTCAAG
CACCCGTTGACAGTCTCAGACCGACGAACGCCGGTGTCGACTCGAAAG
GGACCGACGATCACCTCACCACCAGCATGGATAAGGCATCTGTAGAGA
GTCAGCTTCCGAGAAGAGAGCCATTGGAGACGGAGCCAGATGAACAAG
AAGAAGTTCATTTCAGGAAGCGAGGCGTCCGTTCCGACGCTGAAGTGA
CTGACGACAACATCTACGAGGAGCACACTGATCGTAAAGTGGTTCCGA
GGAAGTCGGAGGGCAAGCGAAGCTTCAAAGACTTGCTGAAGAAGCTC
GCGCTGCCGGCTGTTGGTATGGGTGCATCGTATTTTGCCGCTGATAG
AATTCTGCCGGAACTAACAGAGCAGCAACAGGCAGGCGAAGAACCCCT
AACCACCAGCCAGAATGTGAACACTGTGTTAGGCTTCGCAGCGCTTGC
TGCTGCCGCAGCGTTCCTTGGCATGGGTCTCACGAGGACGTACCGAC
ATTTTTCCCCACGCAAAAACAGATCACGGCAGCCTGCACTCGAGCAAG
AGGTGCCTGAATCAGGCGAAGATGGGGAGGATGCCCGCCATGTCCCG
AACGCGCCTGAGAGGCAGACAGACACAGAAGAGTGA

>TgCgCa1
ATGGCCCGACACGCAATTTTTTTCGCGCTTTGTGTTTTAGGCCTGGT
GGCGGCGGCTTTGCCCCAGTTCGCTACCGCGGCCACCGCGTCAGATG

ACGAACTGATGAGTCGAATCCGAAATTCTGACTTTTTCGATGGTCAAG
CACCCGTTGACAGTCTCAGACCGACGAACGCCGGTGTCGACTCGAAAG
GGACCGACGATCACCTCACCACCAGCATGGATAAGGCATCTGTAGAGA
GTCAGCTTCCGAGAAGAGAGCCATTGGAGACGGAGCCAGATGAACAAG
AAGAAGTTCATTTCAGGAAGCGAGGCGTCCGTTCCGACGCTGAAGTGA
CTGACGACAACATCTACGAGGAGCACACTGATCGTAAAGTGGTTCCGA
GGAAGTCGGAGGGCAAGCGAAGCTTCAAAGACTTGCTGAAGAAGCTC
GCGCTGCCGGCTGTTGGTATGGGTGCATCGTATTTTGCCGCTGATAG
AATTCTGCCGGAACTAACAGAGCAGCAACAGACAGGCGAAGAACCCCT
AACCACCGGCCAGAATGTGAGCACTGTGTTAGGCTTCGCAGCGCTTGC
TGCTGCCGCAGCGTTCCTTGGCATGGGTCTCACGAGGACGTACCGAC
ATTTTTCCCCACGCAAAAACAGATCACGGCAGCCTGCACTCGAGCAAG
AGGTGCCTGAATCAGGCGAAGATGGGGAGGATGCCCGCCAGTAG

>ARI
ATGGCCCGACACGCAATTTTTTTCGCGCTTTGTGTTTTAGGCCTGGT
GGCGGCGGCTTTGCCCCAGTTCGCTACCGCGGCCACCGCGTCAGATG
ACGAACTGATGAGTCGAATCCGAAATTCTGACTTTTTCGATGGTCAAG
CACCCGTTGACAGTCTCAGACCGACGAACGCCGGTGTCGACTCGAAAG
GGACCGACGATCACCTCACCACCAGCATGGATAAGGCATCTGTAGAGA
GTCAGCTTCCGAGAAGAGAGCCATTGGAGACGGAGCCAGATGAACAAG
AAGAAGTTCATTTCAGGAAGCGAGGCGTCCGTTCCGACGCTGAAGTGA
CTGACGACAACATCTACGAGGAGCACACTGATCGTAAAGTGGTTCCGA
GGAAGTCGGAGGGCAAGCGAAGCTTCAAAGACTTGCTGAAGAAGCTC
GCGCTGCCGGCTGTTGGTATGGGTGCATCGTATTTTGCCGCTGATAG
AATGCTGCCGGAACTAACAGAGCAGCAACAGACAGGCGAAGAACCCCT
AACCACCGGCCAGAATGTGAACACTGTGATAGGCTTCGCAGCGCTTGC
TGCTGCCGCAGCGTTCCTTGGCATGGGTATCACGAGGACGTACCGACA
TTTCTCCCCACGCAAAAACAGATCACGGCAGCCTGCACTCGAGCACGA
GGTGCCTGAATCAGGCGAAGATGGGGAGGATGCCCGCCAGTAG

>TgCtPRC2
ATGGCCCGACACGCAATTTTTTTCGCGCTTTGTGTTTTAGGCCTGGT
GGCGGCGGCTTTGCCCCAGTTCGCTACCGCGGCCACCGCGTCAGATG
ACGAACTGATGAGTCGAATCCGAAATTCTGACTTTTTCGATGGTCAAG
CACCCGTTGACAGTCTCAGACCGACGAACGCCGGTGTCGACTCGAAAG

GGACCGACGATCACCTCACCACCAGCATGGATAAGGCATCTGTAGAGA
GTCAGCTTCCGAGAAGAGAGCCATTGGAGACGGAGCCAGATGAANNN
NAAGAAGTTCATTTCAGGAAGCGAGGCGTCCGTTCCGACGCTGAAGT
GACTGACGACAACATCTACGAGGAGCACACTGATCGTAAAGTGGTTCC
GAGGAAGTCGGAGGGCAAGCGAAGCTTCAAAGACTTGCTGAAGAAGC
TCGCGCTGAGAACCCCTAACCACCGGCCAGAATGTGAGCACTGTGTTA
GGCTTCGCAGCGCTTGCTGCTGCCGCAGCGTTCCTTGGCAAGAACCC
CTAACCACCGGCCAGAATGTGAGCACTGTGTTAGGCTTCGCAGCGCTT
GCTGCTGCCGCAGCGTTCCTTGGCATGGGTCTCACGAGGACGTACCG
ACATTTTTCCCCACGCAAAAACAGATCACGGCAGCCTGCACTCGAGCA
AGAGGTGCCTGAATCAGGCGAAGATGGGGAGGATGCCCGCCAGTAG

>GAB2-2007-GAL-DOM2
AACGTTTTGTATTAAAAGGGATTACTGCGGCGTCTCATTTCCAAAATG
GCCCGACACGCAATTTTTTCCGCGCTTTGTGTTTTAGGCCTGGTGGC
GGCGGCTTTGCCCCAGTTCGCTACCGCGGCCACCGCGTCAGATGACG
AACTGATGAGTCGAATCCGAAATTCTGACTTTTTCGATGGTCAAGCAC
CCGTTGACAGTCTCAGACCGACGAACGCCGGTGTCGACTCGAAAGGG
ACCGACGATCACCTCACCACCAGCATGGATAAGGCATCTGTAGAGAGT
CAGCTTCCGAGAAGAGAGCCATTGGAGACGGAGCCAGATGAACAAGAA
GAAGTTCATTTCAGGAAGCGAGGCGTCCGTTCCGACGCTGAAGTGAC
TGACGACAACATCTACGAGGAGCACACTGATCGTAAGGTGGTTCCGAG
GAAGTCGGAGGGCAAGCGAAGCTTCAAAGACTTGCTGAAGAAGCTCG
CGCTGCCGGCTGTTGGTATGGGTGCATCGTATTTTGCCGCTGATAGA
CTTGTGCCGGAACTAACAGAGGAGCAACAGAGAGGCGACGAACCCCTA
ACCACCGGCCAGAATGTGGGCACTGTGTTAGGCTTCGCAGCGCTTGC
TGCTGCCGCAGCGTTCCTTGGCATGGGTCTCACGAGGACGTACCGAC
ATTTTTCCCCACGCAAAAACAGATCACGGCAGCCTGCACTCGAGCAAG
AGGTGCCTGAATCAGGCGAAGATGGGGAGGATGCCCGCCAGTAGGAT
ATGGGGGCTAATAAAAGTGAGTAGGAGCTCGAGGACAGTGTCCCGAAC
GCGCCTGAGAGGCAGACAGACACAGAAGAGTGAAGAAAAACAACATGG
TATTACGTGCGGTGAGTGTTTGCTG>TgCtCo5AACGTTTTGTATTAAAA
GGGTTTACTGCGGCGTCTCATTTCCAAAATGGCCCGACACGCAATTTT
TTTCGCGCTTTGTGTTTTAGGCCTGGTGGCGGCGGCTTTGCCCCAGT
TCGCTACCGCGGCCACCGCGTCGGATGACGAACTGATGAGTCGAATCC
GAAATTCTGACTTTTTCGATGGTCAAGCACCCGTTGACAGTCTCAGAC

CGACGAACGCCGGTGTCGACTCGAAAGGGACCGACGATCACCTCACCA
CCAGCATGGATAAGGCATCTGTAGAGAGTCAGCTTCCGAGAAGAGAGC
CATTGGAGACGGAGCCAGATGAACAAGAAGAAGTTCATTTCAGGAAGC
GAGGCGTCGGTTCCGACGCTGAAGTGACTGACGACCACATCTACGAG
GAGAACACTGATCGTAAAGTGGTTCCGAGGAAGTCGGAGGGCAAGCG
AAGCTTCAAAGACTTGCTGAAGAAGCTCGCGCTGCCGGCTGTTGGTA
TGGGTGCATCGTATTTTGCCGCTGATAGAATTCTGCCGGAACTAACAG
AGCAGCAACAGACAGGCGACGAACCCCTATCCACCGGCCAGAATGTGA
GCACTGTGATAGGCTTCGCAGCGCTTGCTGCTGCCGTAGCGTTCCTT
GGCCTGGGTATCAAGAGGACGTACCGACATTTTTCCCCACGCAAAAAC
AGATCACGGCAGCCTGCACCCGAGCACGAGGTGCCTGAATCAGGCGAA
GATAGGGAGGATGCCCGCCAGTAGGATATGGGGGCTAATAAAGTGAG
TAGGAGCTCGAGGACAGTGTCCCGAACGCGCCTGAGAGGCAGAGAGA
CACAGAAGAGTGAAGAAAAACAACATGGTATTACGTGCGGTGAGTGTT
TGCTG

>TgCatJpOk1
CGTCTCATTTCCAAAATGGCCCGACACGCAATTTTTTTCGCGCTTTGT
GTTTTAGGCCTGGTGGCGGCGGCTTTGCCCCAGTTCGCTACCGCGGC
CACCGCGTCGGATGACGAACTGATGAGTCGAATCCGAAATTCTGACTT
TTTCGATGGTCAAGCACCCGTTGACAGTCTCAGACCGACGAACGCCGG
TGTCGACTCGAAAGGGACCGACGATCACCTCACCACCAGCATGGATAA
GGCATCTGTAGAGAGTCAGCTTCCGAGAAGAGAGCCATTGGAGACGGA
GCCAGATGAACAAGAAGAAGTTCATTTCAGGAAGCGAGGCGTCGGTTC
CGACGCTGAAGTAACTGACGACCACATCTACGAGGAGAACACTGATCG
TAAAGTGGTTCCGAGGAAGTCGGAGGGCAAGCGAAGCTTCAAAGACT
TGCTGAAGAAGCTCGCGCTGCCGGCTGTTGGTATGGGTGCATCGTAT
TTTGCCGCTGATAGAATTCTGCCGGAACTAACAGAGCAGCAACAGACA
GGCGACGAACCCCTATCCACCGGCCAGAATGTGAGCACTGTGATAGGC
TTCGCAGCGCTTGCTGCTGCCGTAGCGTTCCTTGGCCTGGGTATCAA
GAGGACGTACCGACATTTTTCCCCACGCAAAAACAGATCACGGCAGCC
TGCACCCGAGCACGAGGTGCCTGAATCAGGCGAAGATAGGGAGGATG
CCCGCCAGTAGGATATGGGGGCTAATAAAGTGAGTAGGAGCTCGAGG
ACAGTGTCCCGAACGCGCCTGAGAGGCAGAGAGACACAGAAGAGTGAA
GAAAAACAACATGGTATTACGTGCGGTGAG

>TgCatJpOk2
AAATGGCCCGACACGCAATTTTTTTCGCGCTTTGTGTTTTAGGCCTGG
TGGCGGCGGCTTTGCCCCAGTTCGCTACCGCGGCCACCGCGTCGGAT
GACGAACTGATGAGTCGAATCCGAAATTCTGACTTTTTCGATGGTCAA
GCACCCGTTGACAGTCTCAGACCGACGAACGCCGGTGTCGACTCGAAA
GGGACCGACGATCACCTCACCACCAGCATGGATAAGGCATCTGTAGAG
AGTCAGCTTCCGAGAAGAGAGCCATTGGAGACGGAGCCAGATGAACAA
GAAGAAGTTCATTTCAGGAAGCGAGGCGTCGGTTCCGACGCTGAAGT
AACTGACGACCACATCTACGAGGAGAACACTGATCGTAAAGTGGTTCC
GAGGAAGTCGGAGGGCAAGCGAAGCTTCAAAGACTTGCTGAAGAAGC
TCGCGCTGCCGGCTGTTGGTATGGGTGCATCGTATTTTGCCGCTGAT
AGAATTCTGCCGGAACTAACAGAGCAGCAACAGACAGGCGACGAACCC
CTATCCACCGGCCAGAATGTGAGCACTGTGATAGGCTTCGCAGCGCTT
GCTGCTGCCGTAGCGTTCCTTGGCCTGGGTATCAAGAGGACGTACCG
ACATTTTTCCCCACGCAAAAACAGATCACGGCAGCCTGCACCCGAGCA
CGAGGTGCCTGAATCAGGCGAAGATAGGGAGGATGCCCGCCAGTAGG
ATATGGGGGCTAATAAAAGTGAGTAGGAGCTCGAGGACAGTGTCCCGA
ACGCGCCTGAGAGGCAGAGAGACACAGAAGAGTGAAGAAAAACAA

>TgCatJpOk3
TTTTGTATTAAAAGGGTTTACTGCGGCGTCTCATTTCCAAAATGGCCC
GACACGCAATTTTTTTCGCGCTTTGTGTTTTAGGCCTGGTGGCGGCG
GCTTTGCCCCAGTTCGCTACCGCGGCCACCGCGTCAGATGACGAACTG
ATGAGTCGAATCCGAAATTCTGACTTTTTCGATGGTCAAGCACCCGTT
GACAGTCTCAGACCGACGAACGCCGGTGTCGACTCGAAAGGGACCGAC
GATCACCTCACCACCAGCATGGATAAGGCATCTGTAGAGAGTCAGCTT
CCGAGAAGAGAGCCATTGGAGACGGAGCCAGATGAACAAGAAGTTCAT
TTCAGGAAGCGAGGCGTCCGTTCCGACGCTGAAGTGACTGACGACAA
CATCTACGAGGAGCACACTGATCGTAAAGTGGTTCCGAGGAAGTCGGA
GGGCAAGCGAAGCTTCAAAGACTTGCTGAAGAAGCTCGCGCTGCCGG
CTGTTGGTATGGGTGCATCGTATTTTGCCGCTGATAGAATTCTGCCGG
AACTAACAGAGCAGCAACAGACAGGCGAAGAACCCCTAACCACCGGCC
AGAATGTGAGCACTGTGTTAGGCTTCGCAGCGCTTGCTGCTGCCGCA
GCGTTCCTTGGCATGGGTCTCACGAGGACGTACCGACATTTTTCCCCA
CGCAAAAACAGATCACGGCAGCCTGCACTCGAGCAAGAGGTGCCTGAA
TCAGGCGAAGATGGGGAGGATGCCCGCCAGTAGGATATGGGGGCTAA

TAAAAGTGAGTAGGAGCTCGAGGACAGTGTCCCGAACGCGCCTGAGAG
GCAGACAGACACAGAAGAGTGAAGAAAAACAACA

>TgCatJpOk4
TTTTGTATTAAAAGGGTTTACTGCGGCGTCTCATTTCCAAAATGGCCC
GACACGCAATTTTTTTCGCGCTTTGTGTTTTAGGCCTGGTGGCGGCG
GCTTTGCCCCAGTTCGCTACCGCGGCCACCGCGTCAGATGACGAACTG
ATGAGTCGAATCCGAAATTCTGACTTTTTCGATGGTCAAGCACCCGTT
GACAGTCTCAGACCGACGAACGCCGGTGTCGACTCGAAAGGACCGAC
GATCACCTCACCACCAGCATGGATAAGGCATCTGTAGAGAGTCAGCTT
CCGAGAAGAGAGCCATTGGAGACGGAGCCAGATGAACAAGAAGTTCAT
TTCAGGAAGCGAGGCGTCCGTTCCGACGCTGAAGTGACTGACGACAA
CATCTACGAGGAGCACACTGATCGTAAAGTGGTTCCGAGGAAGTCGGA
GGGCAAGCGAAGCTTCAAAGACTTGCTGAAGAAGCTCGCGCTGCCGG
CTGTTGGTATGGGTGCATCGTATTTTGCCGCTGATAGAATTCTGCCGG
AACTAACAGAGCAGCAACAGACAGGCGAAGAACCCCTAACCACCGGCC
AGAATGTGAGCACTGTGTTAGGCTTCGCAGCGCTTGCTGCTGCCGCA
GCGTTCCTTGGCATGGGTCTCACGAGGACGTACCGACATTTTTCCCCA
CGCAAAAACAGATCACGGCAGCCTGCACTCGAGCAAGAGGTGCCTGAA
TCAGGCGAAGATGGGGAGGATGCCCGCCAGTAGGATATGGGGGCTAA
TAAAAGTGAGTAGGAGCTCGAGGACAGTGTCCCGAACGCGCCTGAGAG
GCAGACAGACACAGAAGAGTGAAGAAAAACAACA

>TgCatJpTy1/k-3
TCGAGTTTTGTTGATGATTGAAGTACCCTGTATTGGGGCTTGCTAACG
TTTTGTATTAAAAGGGTTTACTGCGGCGTCTCATTTCCAAAATGGCCC
GACACGCAATTTTTTTCGCGCTTTGTGTTTTAGGCCTGGTGGCGGCG
GCTTTGCCCCAGTTCGCTACCGCGGCCACCGCGTCAGATGACGAACTG
ATGAGTCGAATCCGAAATTCTGACTTTTTCGATGGTCAAGCACCCGTT
GACAGTCTCAGACCGACGAACGCCGGTGTCGACTCGAAAGGACCGAC
GATCACCTCACCACCAGCATGGATAAGGCATCTGTAGAGAGTCAGCTT
CCGAGAAGAGAGCCATTGGAGACGGAGCCAGATGAACnnnAAGAAGTT
CATTTCAGGAAGCGAGGCGTCCGTTCCGACGCTGAAGTGACTGACGA
CAACATCTACGAGGAGCACACTGATCGTAAAGTGGTTCCGAGGAAGTC
GGAGGGCAAGCGAAGCTTCAAAGACTTGCTGAAGAAGCTCGCGCTGC
CGGCTGTTGGTATGGGTGCATCGTATTTTGCCGCTGATAGAATTCTGC

CGGAACTAACAGAGCAGCAACAGACAGGCGAAGAACCCCTAACCACCG
GCCAGAATGTGAGCACTGTGTTAGGCTTCGCAGCGCTTGCTGCTGCC
GCAGCGTTCCTTGGCATGGGTCTCACGAGGACGTACCGACATTTTTCC
CCACGCAAAAACAGATCACGGCAGCCTGCACTCGAGCAAGAGGTGCCT
GAATCAGGCGAAGATGGGGAGGATGCCCGCCAGTAGGATATGGGGGC
TAATAAAAGTGAGTAGGAGCTCGAGGACAGTGTCCCGAACGCGCCTGA
GAGGCAGACAGACACAGAAGAGTGAAGAAAAACAACATGGTATTACGT
GCGGTGAGTGTTTGCTGTCACG

>TgCatJpGi1/TaJ
GTTTTGTATTNAAAGGGTTTACTGCGGCGTCTCATTTCCAAAATGGCC
CGACACGCAATTTTTTTCGCGCTTTGTGTTTTAGGCCTGGTGGCGGC
GGCTTTGCCCCAGTTCGCTACCGCGGCCACCGCGTCGGATGACGAAC
TGATGAGTCGAATCCGAAATTCTGACTTTTTCGATGGTCAAGCACCCG
TTGACAGTCTCAGACCGACGAACGCCGGTGTCGACTCGAAAGGGACC
GACGATCACCTCACCACCAGCATGGATAAGGCATCTGTAGAGAGTCAG
CTTCCGAGAAGAGAGCCATTGGAGACGGAGCCAGATGAACAAGAAGAA
GTTCATTTCAGGAAGCGAGGCGTCGGTTCCGACGCTGAAGTGACTGA
CGACCACATCTACGAGGAGAACACTGATCGTAAAGTGGTTCCGAGGAA
GTCGGAGGGCAAGCGAAGCTTCAAAGACTTGCTGAAGAAGCTCGCGC
TGCCGGCTGTTGGTATGGGTGCATCGTATTTTGCCGCTGATAGAATTC
TGCCGGAACTAACAGAGCAGCAACAGACAGGCGACGAACCCCTATCCA
CCGGCCAGAATGTGAGCACTGTGATAGGCTTCGCAGCGCTTGCTGCT
GCCGTAGCGTTCCTTGGCCTGGGTATCAAGAGGACGTACCGACATTTT
TCCCCACGCAAAAACAGATCACGGCAGCCTGCACCCGAGCACGAGGTG
CCTGAATCAGGCGAAGATAGGGAGGATGCCCGCCAGTAGGATATGGG
GGCTAATAAAAGTGAGTAGGAGCTCGAGGACAGTGTCCCGAACGCGCC
TGAGAGGCAGAGAGACACAGAAGAGTGAAGAAAAACAACATGGTATTA
CGTGCGGTGA

>TgGoatJpOk1
CGTCTCATTTCCAAAATGGCCCGACACGCAATTTTTTTCGCGCTTTGT
GTTTTAGGCCTGGTGGCGGCGGCTTTGCCCCAGTTTCGCTACCGCGG
CCACCGCGTCGGATGACGAACTGATGAGTCGAATCCGAAATTCTGACT
TTTTCGATGGTCCAAGCACCCGTTGACAGTCTCAGACCGACGAACGCC
GGTGTCGACTCGAAAGGGACCGACGATCACCTCACCACCAGCATGGAT

AAGGCATCTGTAGAGAGTCAGCTTCCGAGAAGAGAGCCATTGGAGACG
GAGCCAGATGAACAAGAAGAAGTTCATTTCAGGAAGCGAGGCGTCGGT
TCCGACGCTGAAGTAACTGACGACCACATCTACGAGGAGAACACTGAT
CGTAAAGTGGTTCCGAGGAAGTCGGAGGGCAAGCGAAGCTTCAAAGA
CTTGCTGAAGAAGCTCGCGCTGCCGGCTGTTGGTATGGGTGCATCGT
ATTTTGCCGCTGATAGAATTCTGCCGGAACTAACAGAGCAGCAACAGA
CAGGCGACGAACCCCTATCCACCGGCCAGAATGTGAGCACTGTGATAG
GCTTCGCAGCGCTTGCTGCTGCCGTAGCGTTCCTTGGCCTGGGTATC
AAGAGGACGTACCGACATTTTTCCCCACGCAAAAACAGATCACGGCAG
CCTGCACCCGAGCACGAGGTGCCTGAATCAGGCGAAGATAGGGAGGA
TGCCCGCCAGTAGGATATGGGGGGCTAATAAAAGTGAGTAGGAGCTCG
AGGACAGTGTCCCGAACGCGCCTGAGAGGCAGAGAGACACAGAAGAGT
GA

>TgGoatJpOk2
GGCGTCTCATTTCCAAAATGGCCCGACACGCAATTTTTTTCGCGCTTT
GTGTTTTAGGCCTGGTGGCGGCGGCTTTGCCCCAGTTCGCTACCGCG
GCCACCGCGTCGGATGACGAACTGATGAGTCGAATCCGAAATTCTGAC
TTTTTCGATGGTCCAAGCACCCGTTGACAGTCTCAGACCGACGAACGC
CGGTGTCGACTCGAAAGGGACCGACGATCACCTCACCACCAGCATGGA
TAAGGCATCTGTAGAGAGTCAGCTTCCGAGAAGAGAGCCATTGGAGAC
GGAGCCAGATGAACAAGAAGAAGTTCATTTCAGGAAGCGAGGCGTCG
GTTCCGACGCTGAAGTAACTGACGACCACATCTACGAGGAGAACACTG
ATCGTAAAGTGGTTCCGAGGAAGTCGGAGGGCAAGCGAAGCTTCAAA
GACTTGCTGAAGAAGCTCGCGCTGCCGGCTGTTGGTATGGGTGCATC
GTATTTTGCCGCTGATAGAATTCTGCCGGAACTAACAGAGCAGCAACA
GACAGGCGACGAACCCCTATCCACCGGCCAGAATGTGAGCACTGTGAT
AGGCTTCGCAGCGCTTGCTGCTGCCGTAGCGTTCCTTGGCCTGGGTA
TCAAGAGGACGTACCGACATTTTTCCCCACGCAAAAACAGATCACGGC
AGCCTGCACCCGAGCACGAGGTGCCTGAATCAGGCGAAGATAGGGAG
GATGCCCGCCAGTAGGATATGGGGGCTAATAAAAGTGAGTAGGAGCTC
GAGGACAGTGTCCCGAACGCGCCTGAGAGGCAGAGAGACACAGAAGA
GTGAAGAAAAACAACATGGTATTAC

>TgGoatJpOk3
ATGGCCCGACACGCAATTTTTTTCGCGCTTTGTGTTTTAGGCCTGGT

GGCGGCGGCTTTGCCCCAGTTCGCTACCGCGGCCACCGCGTCGGGAT
GACGAACTGATGAGTCGAATCCGAAATTCTGACTTTTTCGATGGTCAA
GCACCCGTTGACAGTCTCAGACCGACGAACGCCGGTGTCGACTCGAAA
GGGACCGACGATCACCTCACCACCAGCATGGATAAGGCATCTGTAGAG
AGTCAGCTTCCGAGAAGAGAGCCATTGGAGACGGAGCCAGATGAACAA
GAAGAAGTTCATTTCAGGAAGCGAGGCGTCGGTTCCGACGCTGAAGT
AACTGACGACCACATCTACGAGGAGAACACTGATCGTAAAGTGGTTCC
GAGGAAGTCGGAGGGCAAGCGAAGCTTCAAAGACTTGCTGAAGAAGC
TCGCGCTGCCGGCTGTTGGTATGGGTGCATCGTATTTTGCCGCTGAT
AGAATTCTGCCGGAACTAACAGAGCAGCAACAGACAGGCGACGAACCC
CTATCCACCGGCCAGAATGTGAGCACTGTGATAGGCTTCGCAGCGCTT
GCTGCTGCCGTAGCGTTCCTTGGCCTGGGTATCAAGAGGACGTACCG
ACATTTTTCCCCACGCAAAAACAGATCACGGCAGCCTGCACCCGAGCA
CGAGGTGCCTGAATCAGGCGAAGATAGGGAGGATGCCCGCCAGTAGG
ATATGGGGGCTAATAAAAGTGAGTAGGAGCTCGAGGACAGTGTCCCGA
ACGCGCCTGAGAGGCAGAGAGACACAGAAGAGTGAAGAAAAACAACAT
GG

>TgGoatJpOk4
TATTAAAAGGGTTTACTGCGGCGTCTCATTTCCAAAATGGCCCGACAC
GCAATTTTTTTCGCGCTTTGTGTTTTAGGCCTGGTGGCGGCGGCTTT
GCCCCAGTTCGCTACCGCGGCCACCGCGTCAGATGACGAACTGATGAG
TCGAATCCGAAATTCTGACTTTTTCGATGGTCAAGCACCCGTTGACAG
TCTCAGACCGACGAACGCCGGTGTCGACTCGAAAGGGACCGACGATCA
CCTCACCACCAGCATGGATAAGGCATCTGTAGAGAGTCAGCTTCCGAG
AAGAGAGCCATTGGAGACGGAGCCAGATGAACAAGAAGTTCATTTCAG
GAAGCGAGGCGTCCGTTCCGACGCTGAAGTGACTGACGACAACATCTA
CGAGGAGCACACTGATCGTAAAGTGGTTCCGAGGAAGTCGGAGGGCA
AGCGAAGCTTCAAAGACTTGCTGAAGAAGCTCGCGCTGCCGGCTGTT
GGTATGGGTGCATCGTATTTTGCCGCTGATAGAATTCTGCCGGAACTA
ACAGAGCAGCAACAGACAGGCGAAGAACCCCTAACCACCGGCCAGAAT
GTGAGCACTGTGTTAGGCTTCGCAGCGCTTGCTGCTGCCGCAGCGTT
CCTTGGCATGGGTCTCACGAGGACGTACCGACATTTTTCCCCACGCAA
AAACAGATCACGGCAGCCTGCACTCGAGCAAGAGGTGCCTGAATCAGG
CGAAGATGGGGAGGATGCCCGCCAGTAGGATATGGGGGCTAATAAAA
GTGAGTAGGAGCTCGAGGACAGTGTCCCGAACGCGCCTGAGAGGCAG

ACAGACACAGAAGAGTGAAGAAAACAACATGGTATTACGTGCGGTGAG
TGTTTGCT

>TgGoatJpOk5
GGGTTTACTGGGGCGTCTCATTTCCAAAATGGCCCGACACGCAATTTT
TTTCGCGCTTTGTGTTTTAGGCCTGGTGGCGGCGGCTTTGCCCCAGT
TCGCTACCGCGGCCACCGCGTCGGGATGACGAACTGATGAGTCGAAT
CCGAAATTCTGACTTTTTCGATGGTCAAGCACCCGTTGACAGTCTCAG
ACCGACGAACGCCGGTGTCGACTCGAAAGGGACCGACGATCACCTCAC
CACCAGCATGGATAAGGCATCTGTAGAGAGTCAGCTTCCGAGAAGAGA
GCCATTGGAGACGGAGCCAGATGAACAAGAAGAAGTTCATTTCAGGAA
GCGAGGCGTCGGTTCCGACGCTGAAGTAACTGACGACCACATCTACGA
GGAGAACACTGATCGTAAAGTGGTTCCGAGGAAGTCGGAGGGCAAGC
GAAGCTTCAAAGACTTGCTGAAGAAGCTCGCGCTGCCGGCTGTTGGT
ATGGGTGCATCGTATTTTGCCGCTGATAGAATTCTGCCGGAACTAACA
GAGCAGCAACAGACAGGCGACGAACCCCTATCCACCGGCCAGAATGTG
AGCACTGTGATAGGCTTCGCAGCGCTTGCTGCTGCCGTAGCGTTCCT
TGGCCTGGGTATCAAGAGGACGTACCGACATTTTTCCCCACGCAAAAA
CAGATCACGGCAGCCTGCACCCGAGCACGAGGTGCCTGAATCAGGCG
AAGATAGGGAGGATGCCCGCCAGTAGGATATGGGGGCTAATAAAAGTG
AGTAGGAGCTCGAGGACAGTGTCCCGAACGCGCCTGAGAGGCAGAGA
GACACAGAAGAGTGAAGA

>TgGoatJpOk6
TTTGTATTAAAAGGGTTTACTGCGGCGTCTCATTTCCAAAATGGCCCG
ACACGCAATTTTTTTCGCGCTTTGTGTTTTAGGCCTGGTGGCGGCGG
CTTTGCCCCAGTTCGCTACCGCGGCCACCGCGTCAGATGACGAACTGA
TGAGTCGAATCCGAAATTCTGACTTTTTCGATGGTCAAGCACCCGTTG
ACAGTCTCAGACCGACGAACGCCGGTGTCGACTCGAAAGGGACCGACG
ATCACCTCACCACCAGCATGGATAAGGCATCTGTAGAGAGTCAGCTTC
CGAGAAGAGAGCCATTGGAGACGGAGCCAGATGAACAAGAAGTTCATT
TCAGGAAGCGAGGCGTCCGTTCCGACGCTGAAGTGACTGACGACAACA
TCTACGAGGAGCACACTGATCGTAAAGTGGTTCCGAGGAAGTCGGAGG
GCAAGCGAAGCTTCAAAGACTTGCTGAAGAAGCTCGCGCTGCCGGCT
GTTGGTATGGGTGCATCGTATTTTGCCGCTGATAGAATTCTGCCGGAA
CTAACAGAGCAGCAACAGACAGGCGAAGAACCCCTAACCACCGGCCAG

AATGTGAGCACTGTGTTAGGCTTCGCAGCGCTTGCTGCTGCCGCAGC
GTTCCTTGGCATGGGTCTCACGAGGACGTACCGACATTTTTCCCCACG
CAAAAACAGATCACGGCAGCCTGCACTCGAGCAAGAGGTGCCTGAATC
AGGCGAAGATGGGGGAGGATGCCCGCCAGTAGGATATGGGGGCTAAT
AAAAGTGAGTAGGAGCTCGAGGACAGTGTCCCGAACGCGCCTGAGAG
GCAGACAGACACAGAAGAGTGAAGAAAAACAACATGGTATTACGTGCG
GT

>TgGoatJpOk7
CTGCGGCGTCTCATTTCCAAAATGGCCCGACACGCAATTTTTTTCGCG
CTTTGTGTTTTAGGCCTGGTGGCGGCGGCTTTGCCCCAGTTCGCTAC
CGCGGCCACCGCGTCAGATGACGAACTGATGAGTCGAATCCGAAATTC
TGACTTTTTCGATGGTCCAAGCACCCGTTGACAGTCTCAGACCGACGA
ACGCCGGTGTCGACTCGAAAGGGACCGACGATCACCTCACCACCAGCA
TGGATAAGGCATCTGTAGAGAGTCAGCTTCCGAGAAGAGAGCCATTGG
AGACGGAGCCAGATGAACAAGAAGTTCATTTCAGGAAGCGAGGCGTCC
GTTCCGACGCTGAAGTGACTGACGACAACATCTACGAGGAGCACACTG
ATCGTAAAGTGGTTCCGAGGAAGTCGGAGGGCAAGCGAAGCTTCAAA
GACTTGCTGAAGAAGCTCGCGCTGCCGGCTGTTGGTATGGGTGCATC
GTATTTTGCCGCTGATAGAATTCTGCCGGAACTAACAGAGCAGCAACA
GACAGGCGAAGAACCCCTAACCACCGGCCAGAATGTGAGCACTGTGTT
AGGCTTCGCAGCGCTTGCTGCTGCCGCAGCGTTCCTTGGCATGGGTC
TCACGAGGACGTACCGACATTTTTCCCCACGCAAAAACAGATCACGGC
AGCCTGCACTCGAGCAAGAGGTGCCTGAATCAGGCGAAGATGGGGAG
GATGCCCGCCAGTAGGATATGGGGGCTAATAAAAGTGAGTAGGAGCTC
GAGGACAGTGTCCCGAACGCGCCTGAGAGGCAGACAGACACAGAAGAG
TGAAGAAAAACAACATGGTATTACGTGC

>TgGoatJpOk8
AAATGGCCCGACACGCAATTTTTTTCGCGCTTTGTGTTTTAGGCCTGG
TGGCGGCGGCTTTGCCCCAGTTCGCTACCGCGGCCACCGCGTCAGAT
GACGAACTGATGAGTCGAATCCGAAATTCTGACTTTTTCGATGGTCAA
GCACCCGTTGACAGTCTCAGACCGACGAACGCCGGTGTCGACTCGAAA
GGGACCGACGATCACCTCACCACCAGCATGGATAAGGCATCTGTAGAG
AGTCAGCTTCCGAGAAGAGAGCCATTGGAGACGGAGCCAGATGAACAA
GAAGAAGTTCATTTCAGGAAGCGAGGCGTCCGTTCCGACGCTGAAGT

GACTGACGACAACATCTACGAGGAGCACACTGATCGTAAAGTGGTTCC
GAGGAAGTCGGAGGGCAAGCGAAGCTTCAAAGACTTGCTGAAGAAGC
TCGCGCTGCCGGCTGTTGGTATGGGTGCATCGTATTTTGCCGCTGAT
AGAATTCTGCCGGAACTAACAGAGCAGCAACAGACAGGCGAAGAACCC
CTAACCACCGGCCAGAATGTGAGCACTGTGTTAGGCTTCGCAGCGCTT
GCTGCTGCCGCAGCGTTCCTTGGCATGGGTCTCACGAGGACGTACCG
ACATTTTTCCCCACGCAAAAACAGATCACGGCAGCCTGCACTCGAGCA
AGAGGTGCCTGAATCAGGCAAAGATGGGGAGGATGCCCGCCAGTAGG
ATATGGGGGCTAATAAAAGTGAGTAGGAGCTCGAGGACAGTGTCCCGA
ACGCGCCTGAGAGGCAGACAGACACAGAAGAGTGAAGAAAAACAACAT
GGTATT

>TgGoatJpOk9
CGTCTCATTTCCAAAATGGCCCGACACGCAATTTTTTTCGCGCTTTGT
GTTTTAGGCCTGGTGGCGGCGGCTTTGCCCCAGTTCGCTACCGCGGC
CACCGCGTCAGATGACGAACTGATGAGTCGAATCCGAAATTCTGACTT
TTTCGATGGTCAAGCACCCGTTGACAGTCTCAGACCGACGAACGCCGG
TGTCGACTCGAAAAGGGACCGACGATCACCTCACCACCAGCATGGATA
AGGCATCTGTAGAGAGTCAGCTTCCGAGAAGAGAGCCATTGGAGACGG
AGCCAGATGAACAAGAAGTTCATTTCAGGAAGCGAGGCGTCCGTTCCG
ACGCTGAAGTGACTGACGACAACATCTACGAGGAGCACACTGATCGTA
AAGTGGTTCCGAGGAAGTCGGAGGGCAAGCGAAGCTTCAAAGACTTG
CTGAAGAAGCTCGCGCTGCCGGCTGTTGGTATGGGTGCATCGTATTT
TGCCGCTGATAGAATTCTGCCGGAACTAACAGAGCAGCAACAGACAGG
CGAAGAACCCCTAACCACCGGCCAGAATGTGAGCACTGTGTTAGGCTT
CGCAGCGCTTGCTGCTGCCGCAGCGTTCCTTGGCATGGGTCTCACGA
GGACGTACCGACATTTTTCCCCACGCAAAAACAGATCACGGCAGCCTG
CACTCGAGCAAGAGGTGCCTGAATCAGGCGAAGATGGGGGAGGATGC
CCGCCAGTAGGATATGGGGGCTAATAAAAGTGAGTAGGAGCTCGAGGA
CAGTGTCCCGAACGCGCCTGAGAGGCAGACAGACACAGAAGANTGAAG
AAAAACAACATGGTATTACGTGCCGGTGAGTGTTTGCTGTCACGT

>TgGoatJpOk10
TTACTGCGGCGTCTCATTTCCAAAATGGCCCGACACGCAATTTTTTTC
GCGCTTTGTGTTTTAGGCCTGGTGGCGGCGGCTTTGCCCCAGTTCGC
TACCGCGGCCACCGCGTCAGATGACGAACTGATGAGTCGAATCCGAAA

TTCTGACTTTTTCGATGGTCAAGCACCCGTTGACAGTCTCAGACCGAC
GAACGCCGGTGTCGACTCGAAAGGGACCGACGATCACCTCACCACCAG
CATGGATAAGGCATCTGTAGAGAGTCAGCTTCCGAGAAGAGAGCCATT
GGAGACGGAGCCAGATGAACAAGAAGTTCATTTCAGGAAGCGAGGCGT
CCGTTCCGACGCTGAAGTGACTGACGACAACATCTACGAGGAGCACAC
TGATCGTAAAGTGGTTCCGAGGAAGTCGGAGGGCAAGCGAAGCTTCA
AAGACTTGCTGAAGAAGCTCGCGCTGCCGGCTGTTGGTATGGGTGCA
TCGTATTTTGCCGCTGATAGAATTCTGCCGGAACTAACAGAGCAGCAA
CAGACAGGCGAAGAACCCCTAACCACCGGCCAGAATGTGAGCACTGTG
TTAGGCTTCGCAGCGCTTGCTGCTGCCGCAGCGTTCCTTGGCATGGG
TCTCACGAGGACGTACCGACATTTTTCCCCACGCAAAAACAGATCACG
GCAGCCTGCACTCGAGCAAGAGGTGCCTGAATCAGGCGAAGATGGGG
GAGGATGCCCGCCAGTAGGATATGGGGGCTAATAAAGTGAGTAGGAG
CTCGAGGACAGTGTCCCGAACGCGCCTGAGAGGCAGACAGACACAGAA
GAGTGAAGAAAAACNACATGGTAT

>TgGoatJpOk11
TTTCCAAAATGGCCCGACACGCAATTTTTTTCGCGCTTTGTGTTTTAG
GCCTGGTGGCGGCGGCTTTGCCCCAGTTCGCTACCGCGGCCACCGCG
TCGGATGACGAACTGATGAGTCGAATCCGAAATTCTGACTTTTTCGAT
GGTCAAGCACCCGTTGACAGTCTCAGACCGACGAACGCCGGTGTCGAC
TCGAAAGGGACCGACGATCACCTCACCACCAGCATGGATAAGGCATCT
GTAGAGAGTCAGCTTCCGAGAAGAGAGCCATTGGAGACGGAGCCAGAT
GAACAAGAAGAAGTTCATTTCAGGAAGCGAGGCGTCGGTTCCGACGCT
GAAGTGACTGACGACCACATCTACGAGGAGAACACTGATCGTAAAGTG
GTTCCGAGGAAGTCGGAGGGCAAGCGAAGCTTCAAAGACTTGCTGAA
GAAGCTCGCGCTGCCGGCTGTTGGTATGGGTGCATCGTATTTTGCCG
CTGATAGAATTCTGCCGGAACTAACAGAGCAGCAACAGACAGGCGACG
AACCCCTATCCACCGGCCAGAATGTGAGCACTGTGATAGGCTTCGCAG
CGCTTGCTGCTGCCGTAGCGTTCCTTGGCCTGGGTATCAAGAGGACG
TACCGACATTTTTCCCCACGCAAAAACAGATCACGGCAGCCTGCACCC
GAGCACGAGGTGCCTGAATCAGGCGAAGATAGGGAGGATGCCCGCCA
GTAGGATATGGGGGCTAATAAAGTGAGTAGGAGCTCGAGGACAGTGT
CCCGAACGCGCCTGAGAGGCAGAGAGACACAGAAGAGTGA

Surface antigen gene 1 (SAG1)

>GT1
ATGTCGGTTTCGCTGCACCACTTCATTATTTCTTCTGGTTTTTTGACG
AGTATGTTTCCGAAGGCAGTGAGACGCGCCGTCACGGCAGGGGTGTT
TGCCGCGCCCACACTGATGTCGTTCTTGCGATGTGGCGTTATGGCAT
CGGATCCCCCTCTTGTTGCCAATCAAGTTGTCACCTGCCCAGATAAAA
AATCGACAGCCGCGGTCATTCTCACACCGACGGAGAACCACTTCACTC
TCAAGTGCCCTAAAACAGCGCTCACAGAGCCTCCCACTCTTGCGTACT
CACCCAACAGGCAAATCTGCCCAGCGGGTACTACAAGTAGCTGTACATC
AAAGGCTGTAACATTGAGCTCCTTGATTCCTGAAGCAGAAGATAGCTG
GTGGACGGGGGATTCTGCTAGTCTCGACACGGCAGGCATCAAACTCAC
AGTTCCAATCGAGAAGTTCCCCGTGACAACGCAGACGTTTGTGGTCGG
TTGCATCAAGGGAGACGACGCACAGAGTTGTATGGTCACGGTGACAGT
ACAAGCCAGAGCCTCATCGGTCGTCAATAATGTCGCAAGGTGCTCCTA
CGGTGCAGACAGCACTCTTGGTCCTGTCAAGTTGTCTGCGGAAGGAC
CCACTACAATGACCCTCGTGTGCGGGAAAGATGGAGTCAAAGTTCCTC
AAGACAACAATCAGTACTGTTCCGGGACGACGCTGACTGGTTGCAACG
AGAAATCGTTCAAAGATATTTTGCCAAAATTAACTGAGAACCCGTGGCA
GGGTAACGCTTCGAGTGATAAGGGTGCCACGCTAACGATCAAGAAGGA
AGCATTTCCAGCCGAGTCAAAAAGCGTCATTATTGGATGCACAGGGGG
ATCGCCTGAGAAGCATCACTGTACCGTGAAACTGGAGTTTGCCGGGGC
TGCAGGGTCAGCAAAATCGGCTGCGGGAACAGCCAGTCACGTTTCCAT
TTTTGCCATGGTGATCGGACTTATTGGCTCTATCGCAGCTTGTGTCGC
GTGA

>ME49
ATGTCGGTTTCGCTGCACCACTTCATTATTTCTTCTGGTTTTTTGGCG
AGTATGTTTCCGAAGGCAGTGAGACGCGCCGTCACGGCAGGGGTGTT
TGCCGCGCCCACACTGATGTCGTTCTTGCGATGTGGCGCTATGGCAT
CGGATCCCCCTCTTGTTGCCAATCAAGTTGTCACCTGCCCAGATAAAA
AATCGACAGCCGCGGTCATTCTCACACCGACGGAGAACCACTTCACTC
TCAAGTGCCCTAAAACAGCGCTCACAGAGCCTCCCACTCTTGCGTACT
CACCCAACAGGCAAATCTGCCCAGCGGGTACTACAAGTAGCTGTACATC
AAAGGCTGTAACATTGAGCTCCTTGATTCCTGAAGCAGAAGATAGCTG
GTGGACGGGGGATTCTGCTAGTCTCGACACGGCAGGCATCAAACTCAC

AGTTCCAATCGAGAAGTTCCCCGTGACAACGCAGACGTTTGTGGTCGG
TTGCATCAAGGGAGACGACGCACAGAGTTGTATGGTCACAGTGACAGT
ACAAGCCAGAGCCTCATCGGTCGTCAATAATGTCGCAAGGTGCTCCTA
CGGTGCAAACAGCACTCTTGGTCCTGTCAAGTTGTCTGCGGAAGGAC
CCACTACAATGACCCTCGTGTGCGGGAAAGATGGAGTCAAAGTTCCTC
AAGACAACAATCAGTACTGTTCCGGGACGACGCTGACTGGTTGCAACG
AGAAATCGTTCAAAGATATTTTGCCAAAATTAAGTGAGAACCCGTGGC
AGGGTAACGCTTCGAGTGATAATGGTGCCACGCTAACGATCAACAAGG
AAGCATTTCCAGCCGAGTCAAAAAGCGTCATTATTGGATGCACAGGGG
GATCGCCTGAGAAGCATCACTGTACCGTGCAACTGGAGTTTGCCGGG
GCTGCAGGGTCAGCAAAATCGTCTGCGGGAACAGCCAGTCACGTTTCC
ATTTTCGCCATGGTGACCGGACTTATTGGCTCTATCGCAGCTTGTGTC
GCGTGA

>VEG
ATGTCGGTTTCGCTGCACCACTTCATTATTTCTTCTGGTTTTTTGGCG
AGTATGTTTCCGAAGGCAGTGAGACGCGCCGTCACGGCAGGGGTGTT
TGCCGCGCCCACACTGATGTCGTTCTTGCGATGTGGCGCTATGGCAT
CGGATCCCCCTCTTGTTGCCAATCAAGTTGTCACCTGCCCAGATAAAA
AATCGACAGCCGCGGTCATTCTCACACCGACGGAGAACCACTTCACTC
TCAAGTGCCCTAAAACAGCGCTCACAGAGCCTCCCACTCTTGCGTACT
CACCCAACAGGCAAATCTGCCCAGCGGGTACTACAAGTAGCTGTACATC
AAAGGCTGTAACATTGAGCTCCTTGATTCCTGAAGCAGAAGATAGCTG
GTGGACGGGGGATTCTGCTAGTCTCGACACGGCAGGCATCAAACTCAC
AGTTCCAATCGAGAAGTTCCCCGTGACAACGCAGACGTTTGTGGTCGG
TTGCATCAAGGGAGACGACGCACAGAGTTGTATGGTCACAGTGACAGT
ACAAGCCAGAGCCTCATCGGTCGTCAATAATGTCGCAAGGTGCTCCTA
CGGTGCAAACAGCACTCTTGGTCCTGTCAAGTTGTCTGCGGAAGGAC
CCACTACAATGACCCTCGTGTGCGGGAAAGATGGAGTCAAAGTTCCTC
AAGACAACAATCAGTACTGTTCCGGGACGACGCTGACTGGTTGCAACG
AGAAATCGTTCAAAGATATTTTGCCAAAATTAAGTGAGAACCCGTGGC
AGGGTAACGCTTCGAGTGATAATGGTGCCACGCTAACGATCAACAAGG
AAGCATTTCCAGCCGAGTCAAAAAGCGTCATTATTGGATGCACAGGGG
GATCGCCTGAGAAGCATCACTGTACCGTGCAACTGGAGTTTGCCGGG
GCTGCAGGGTCAGCAAAATCGTCTGCGGGAACAGCCAGTCACGTTTCC
ATTTTCGCCATGGTGACCGGACTTATTGGCTCTATCGCAGCTTGTGTC

GCGTGA

>MAS
ATGTCGGTTTCGCTGCACCACTTCATTATTTCTTCTGGTTTTTTGACG
AGTATGTTTCCGAAGGCAGTGAGACGCGCCGTCACGGCAGGGGTGTT
TGCCGCGCCCACACTGATGTCGTTCTTGCGATGTGGCGCTATGGCAT
CGGATCCCCCTCTTGTTGCCAATCAAGTTGTCACCTGCCCAGATAAAA
AATCGACAGCCGCGGTCATTCTCACACCGACGGAGAACCACTTCACTC
TCAAGTGCCCTAAAACAGCGCTCACAGAGCCTCCCACTCTTGCGTACT
CACCCAACAGGCAAATCTGCCCAGCGGGTACTACAAGTAGCTGTACATC
AAAGGCTGTAACATTGAGCTCCTTGATTCCTGAAGCAGAAGATAGCTG
GTGGACGGGGGATTCTGCTAGTCTCGACACGGCAGGCATCAAACTCAC
AGTTCCAATCGAGAAGTTCCCCGTGACAACGCAGACGTTTGTGGTCGG
TTGCATCAAGGGAGACGACGCACAGAGTTGTATGGTCACAGTGACAGT
ACAAGCCAGAGCCTCATCGGTCGTCAATAATGTCGCAAGGTGCTCCTA
CGGTGCAAACAGCACTCTTGGTCCTGTCAAGTTGTCTGCGGAAGGAC
CCACTACAATGACCCTCGTGTGCGGGAAAGATGGAGTCAAAGTTCCTC
AAGACAACAATCAGTACTGTTCCGGGACGACGCTGACTGGTTGCAACG
AGAAATCGTTCAAAGATATTTTGCCAAAATTAACTGAGAACCCGTGGCA
GGGTAACGCTTCGAGTGATAAGGGTGCCACGCTAACGATCAAGAAGGA
AGCATTTCCAGCCGAGTCAAAAAGCGTCATTATTGGATGCACAGGGGG
ATCGCCTGAGAAGCATCACTGTACCGTGAAACTGGAGTTTGCCGGGGC
TGCAGGGTCAGCAAAATCGGCTGCGGGAACAGCCAGTCACGTTTCCAT
TTTTGCCATGGTGACCGGACTTATTGGCTCTATCGCAGCTTGTGTCGC
GTGA

>RUB
ATGTCGGTTTCGCTGCACCACTTCATTATTTCTTCTGGTTTTTTGACG
AGTATGTTTCCGAAGGCAGTGAGACGCGCCGTCACGGCAGGGGTGTT
TGCCGCGCCCACACTGATGTCGTTCTTGCGATGTGGCGTTATGGCAT
CGGATCCCCCTCTTGTTGCCAATCAAGTTGTCACCTGCCCAGATAAAA
AATCGACAGCCGCGGTCATTCTCACACCGACGGAGAACCACTTCACTC
TCAAGTGCCCTAAAACAGCGCTCACAGAGCCTCCCACTCTTGCGTACT
CACCCAACAGGCAAATCTGCCCAGCGGGTACTACAAGTAGCTGTACATC
AAAGGCTGTAACATTGAGCTCCTTGATTCCTGAAGCAGAAGATAGCTG
GTGGACGGGGGATTCTGCTAGTCTCGACACGGCAGGCATCAAACTCAC

AGTTCCAATCGAGAAGTTCCCCGTGACAACGCAGACGTTTGTGGTCGG
TTGCATCAAGGGAGATGACGCACAGAGTTGTATGGTCACAGTGACAGT
ACAAGCCAGAGCCTCATCGGTCGTCAATAATGTCGCAAGGTGCTCCTA
CGGTGCAGACAGCACTCTTGGTCCTGTCAAGTTGTCTGCGGAAGGAC
CCACTACAATGACCCTCGTGTGCGGGAAAGATGGAGTCAAAGTTCCTC
AAGACAACAATCAGTACTGTTCCGGGACGACGCTGACTGGTTGCAACG
AGAAATCGTTCAAAGATATTTTGCCAAAATTAACTGAGAACCCGTGGCA
GGGTAACGCTTCGAGTGATAAGGGTGCCACGCTAACGATCAAGAAGGA
AGCATTTCCAGCCGAGTCAAAAAGCGTCATTATTGGATGCACAGGGGG
ATCGCCTGAGAAGCATCACTGTACCGTGAAACTGGAGTTTGCCGGGGC
TGCAGGGTCAGCAAAATCGGCTGCGGGAACAGCCAGTCACGTTTCCAT
TCTTGCCATGGTGATCGGACTTATTGGCTCTATCGCAGCTTGTGTCGC
GTGA

>FOU
ATGTCGGTTTCGCTGCACCACTTCATTATTTCTTCTGGTTTTTTGACG
AGTATGTTTCCGAAGGCAGTGAGACGCGCCGTCACGGCAGGGGTGTT
TGCCGCGCCCACACTGATGTCGTTCTTGCGATGTGGCGTTATGGCAT
CGGATCCCCCTCTTGTTGCCAATCAAGTTGTCACCTGCCCAGATAAAA
AATCGACAGCCGCGGTCATTCTCACACCGACGGAGAACCACTTCACTC
TCAAGTGCCCTAAAACAGCGCTCACAGAGCCTCCCACTCTTGCGTACT
CACCCAACAGGCAAATCTGCCCAGCGGGTACTACAAGTAGCTGTACATC
AAAGGCTGTAACATTGAGCTCCTTGATTCCTGAAGCAGAAGATAGCTG
GTGGACGGGGGATTCTGCTAGTCTCGACACGGCAGGCATCAAACTCAC
AGTTCCAATCGAGAAGTTCCCCGTGACAACGCAGACGTTTGTGGTCGG
TTGCATCAAGGGAGACGACGCACAGAGTTGTATGGTCACAGTGACAGT
ACAAGCCAGAGCCTCATCGGTCGTCAATAATGTCGCAAGGTGCTCCTA
CGGTGCAGACAGCACTCTTGGTCCTGTCAAGTTGTCTGCGGAAGGAC
CCACTACAATGACCCTCGTGTGCGGGAAAGATGGAGTCAAAGTTCCTC
AAGACAACAATCAGTACTGTTCCGGGACGACGCTGACTGGTTGCAACG
AGAAATCGTTCAAAGATATTTTGCCAAAATTAACTGAGAACCCGTGGCA
GGGTAACGCTTCGAGTGATAAGGGTGCCACGCTAACGATCAAGAAGGA
AGCATTTCCAGCCGAGTCAAAAAGCGTCATTATTGGATGCACAGGGGG
ATCGCCTGAGAAGCATCACTGTACCGTGAAACTGGAGTTTGCCGGGGC
TGCAGGGTCAGCAAAATCGGCTGCGGGAACAGCCAGTCACGTTTCCAT
TTTTGCCATGGTGATCGGACTTATTGGCTCTATCGCAGCTTGTGTCGC

GTGA

>CAST
ATGTCGGTTTCGCTGCACCACTTCATTATTTCTTCTGGTTTTTGACG
AGTATGTTTCCGAAGGCAGTGAGACGCGCCGTCACGGCAGGGGTGTT
TGCCGCGCCCACACTGATGTCGTTCTTGCGATGTGGCGTTATGGCAT
CGGATCCCCCTCTTGTTGCCAATCAAGTTGTCACCTGCCCAGATAAAA
AATCGACAGCCGCGGTCATTCTCACACCGACGGAGAACCACTTCACTC
TCAAGTGCCCTAAAACAGCGCTCACAGAGCCTCCCACTCTTGCGTACT
CACCCAACAGGCAAATCTGCCCAGCGGGTACTACAAGTAGCTGTACATC
AAAGGCTGTAACATTGAGCTCCTTGATTCCTGAAGCAGAAGATAGCTG
GTGGACGGGGGATTCTGCTAGTCTCGACACGGCAGGCATCAAACTCAC
AGTTCCAATCGAGAAGTTCCCCGTGACAACGCAGACGTTTGTGGTCGG
TTGCATCAAGGGAGACGACGCACAGAGTTGTATGGTCACAGTGACAGT
ACAAGCCAGAGCCTCATCGGTCGTCAATAATGTCGCAAGGTGCTCCTA
CGGTGCAGACAGCACTCTTGGTCCTGTCAAGTTGTCTGCGGAAGGAC
CCACTACAATGACCCTCGTGTGCGGGAAAGATGGAGTCAAAGTTCCTC
AAGACAACAATCAGTACTGTTCCGGGACGACGCTGACTGGTTGCAACG
AGAAATCGTTCAAAGATATTTTGCCAAAATTAACTGAGAACCCGTGGCA
GGGTAACGCTTCGAGTGATAAGGGTGCCACGCTAACGATCAAGAAGGA
AGCATTTCCAGCCGAGTCAAAAAGCGTCATTATTGGATGCACAGGGGG
ATCGCCTGAGAAGCATCACTGTACCGTGAAACTGGAGTTTGCCGGGGC
TGCAGGGTCAGCAAAATCGGCTGCGGGAACAGCCAGTCACGTTTCCAT
TTTTGCCATGGTGATCGGACTTATTGGCTCTATCGCAGCTTGTGTCGC
GTGA

>TgCatBr5
TGTCGGTTTCGCTGCACCACTTCATTATTTCTTCTGGTTTTTGACGA
GTATGTTTCCGAAGGCAGTGAGACGCGCCGTCACGGCAGGGGTGTTT
GCCGCGCCCACACTGATGTCGTTCTTGCGATGTGGCGTTATGGCATC
GGATCCCCCTCTTGTTGCCAATCAAGTTGTCACCTGCCCAGATAAAAA
ATCGACAGCCGCGGTCATTCTCACACCGACGGAGAACCACTTCACTCT
CAAGTGCCCTAAAACAGCGCTCACAGAGCCTCCCACTCTTGCGTACTC
ACCCAACAGGCAAATCTGCCCAGCGGGTACTACAAGTAGCTGTACATCA
AAGGCTGTAACATTGAGCTCCTTGATTCCTGAAGCAGAAGATAGCTGG
TGGACGGGGGATTCTGCTAGTCTCGACACGGCAGGCATCAAACTCACA

GTTCCAATCGAGAAGTTCCCCGTGACAACGCAGACGTTTGTGGTCGGT
TGCATCAAGGGAGACGACGCACAGAGTTGTATGGTCACAGTGACAGTA
CAAGCCAGAGCCTCATCGGTCGTCAATAATGTCGCAAGGTGCTCCTAC
GGTGCAGACAGCACTCTTGGTCCTGTCAAGTTGTCTGCGGAAGGACC
CACTACAATGACCCTCGTGTGCGGGAAAGATGGAGTCAAAGTTCCTCA
AGACAACAATCAGTACTGTTCCGGGACGACGCTGACTGGTTGCAACGA
GAAATCGTTCAAAGATATTTTGCCAAAATTAACTGAGAACCCGTGGCA
GGGTAACGCTTCGAGTGATAAGGGTGCCACGCTAACGATCAAGAAGGA
AGCATTTCCAGCCGAGTCAAAAAGCGTCATTATTGGATGCACAGGGGG
ATCGCCTGAGAAGCATCACTGTACCGTGAAACTGGAGTTTGCCGGGGC
TGCAGGGTCAGCAAAATCGGCTGCGGGAACAGCCAGTCACGTTTCCAT
TTTTGCCATGGTGATCGGACTTATTGGCTCTATCGCAGCTTGTGTCGC
GTGA

>p89
ATGTCGGTTTCGCTGCACCACTTCATTATTTCTTCTGGTTTTTTGACG
AGTATGTTTCCGAAGGCAGTGAGACGCGCCGTCACGGCAGGGGTGTT
TGCCGCGCCCACACTGATGTCGTTCTTGCGATGTGGCGTTATGGCAT
CGGATCCCCCTCTTGTTGCCAATCAAGTTGTCACCTGCCCAGATAAAA
AATCGACAGCCGCGGTCATTCTCACACCGACGGAGAACCACTTCACTC
TCAAGTGCCCTAAAACAGCGCTCACAGAGCCTCCCACTCTTGCGTACT
CACCCAACAGGCAAATCTGCCCAGCGGGTACTACAAGTAGCTGTACATC
AAAGGCTGTAACATTGAGCTCCTTGATTCCTGAAGCAGAAGATAGCTG
GTGGACGGGGGATTCTGCTAGTCTCGACACGGCAGGCATCAAACTCAC
AGTTCCAATCGAGAAGTTCCCCGTGACAACGCAGACGTTTGTGGTCGG
TTGCATCAAGGGAGACGACGCACAGAGTTGTATGGTCACAGTGACAGT
ACAAGCCAGAGCCTCATCGGTCGTCAATAATGTCGCAAGGTGCTCCTA
CGGTGCAGACAGCACTCTTGGTCCTGTCAAGTTGTCTGCGGAAGGAC
CCACTACAATGACCCTCGTGTGCGGGAAAGATGGAGTCAAAGTTCCTC
AAGACAACAATCAGTACTGTTCCGGGACGACGCTGACTGGTTGCAACG
AGAAATCGTTCAAAGATATTTTGCCAAAATTAACTGAGAACCCGTGGCA
GGGTAACGCTTCGAGTGATAAGGGTGCCACGCTAACGATCAAGAAGGA
AGCATTTCCAGCCGAGTCAAAAAGCGTCATTATTGGATGCACAGGGGG
ATCGCCTGAGAAGCATCACTGTACCGTGAAACTGGAGTTTGCCGGGGC
TGCAGGGTCAGCAAAATCGGCTGCGGGAACAGCCAGTCACGTTTCCAT
TTTTGCCATGGTGATCGGACTTATTGGCTCTATCGCAGCTTGTGTCGC

GTGA

>VAND
ATGTCGGTTTCGCTGCACCACTTCATTATTTCTTCTGGTTTTTTGACG
AGTATGTTTCCGAAGGCAGTGAGACGCGCCGTCACGGCAGGGGTGTT
TGCCGCGCCCACACTGATGTCGTTCTTGCGATGTGGCGTTATGGCAT
CGGATCCCCCTCTTGTTGCCAATCAAGTTGTCACCTGCCCAGATAAAA
AATCGACAGCCGCGGTCATTCTCACACCGACGGAGAACCACTTCACTC
TCAAGTGCCCTAAAACAGCGCTCACAGAGCCTCCCACTCTTGCGTACT
CACCCAACAGGCAAATCTGCCCAGCGGGTACTACAAGTAGCTGTACATC
AAAGGCTGTAACATTGAGCTCCTTGATTCCTGAAGCAGAAGATAGCTG
GTGGACGGGGGATTCTGCTAGTCTCGACACGGCAGGCATCAAACTCAC
AGTTCCAATCGAGAAGTTCCCCGTGACAACGCAGACGTTTGTGGTCGG
TTGCATCAAGGGAGACGACGCACAGAGTTGTATGGTCACAGTGACAGT
ACAAGCCAGAGCCTCATCGGTCGTCAATAATGTCGCAAGGTGCTCCTA
CGGTGCAGACAGCACTCTTGGTCCTGTCAAGTTGTCTGCGGAAGGAC
CCACTACAATGACCCTCGTGTGCGGGAAAGATGGAGTCAAAGTTCCTC
AAGACAACAATCAGTACTGTTCCGGGACGACGCTGACTGGTTGCAACG
AGAAATCGTTCAAAGATATTTTGCCAAAATTAACTGAGAACCCGTGGCA
GGGTAACGCTTCGAGTGATAAGGGTGCCACGCTAACGATCAACAAGGA
AGCATTTCCAGCCGAGTCAAAAAGCGTCATTATTGGATGCACAGGGGG
ATCGCCTGAGAAGCATCACTGTACCGTGAAACTGGAGTTTGCCGGGGC
TGCAGGGTCAGCAAAATCGGCTGCGGGAACAGCCAGTCACGTTTCCAT
TTTTGCCATGGTGATCGGACTTATTGGCTCTATCGCAGCTTGTGTCGC
GTGA

>TgCgCa1
ATGTCGGTTTCGCTGCACCACTTCATTATTTCTTCTGGTTTTTTGACG
AGTATGTTTCCGAAGGCAGTGAGACGCGCCGTCACGGCAGGGGTGTT
TGCCGCGCCCACACTGATGTCGTTCTTGCGATGTGGCGTTATGGCAT
CGGATCCCCCTCTTGTTGCCAATCAAGTTGTCACCTGCCCAGATAAAA
AATCGACAGCCGCGGTCATTCTCACACCGACGGAGAACCACTTCACTC
TCAAGTGCCCTAAAACAGCGCTCACAGAGCCTCCCACTCTTGCGTACT
CACCCAACAGGCAAATCTGCCCAGCGGGTACTACAAGTAGCTGTACATC
AAAGGCTGTAACATTGAGCTCCTTGATTCCTGAAGCAGAAGATAGCTG
GTGGACGGGGGATTCTGCTAGTCTCGACACGGCAGGCATCAAACTCAC

AGTTCCAATCGAGAAGTTCCCCGTGACAACGCAGACGTTTGTGGTCGG
TTGCATCAAGGGAGACGACGCACAGAGTTGTATGGTCACAGTGACAGT
ACAAGCCAGAGCCTCATCGGTCGTCAATAATGTCGCAAGGTGCTCCTA
CGGTGCAGACAGCACTCTTGGTCCTGTCAAGTTGTCTGCGGAAGGAC
CCACTACAATGACCCTCGTGTGCGGGAAAGATGGAGTCAAAGTTCCTC
AAGACAACAATCAGTACTGTTCCGGGACGACGCTGACTGGTTGCAACG
AGAAATCGTTCAAAGATATTTTGCCAAAATTAACTGAGAACCCGTGGCA
GGGTAACGCTTCGAGTGATAAGGGTGCCACGCTAACGATCAAGAAGGA
AGCATTTCCAGCCGAGTCAAAAAGCGTCATTATTGGATGCACAGGGGG
ATCGCCTGAGAAGCATCACTGTACCGTGAAACTGGAGTTTGCCGGGGC
TGCAGGGTCAGCAAAATCGGCTGCGGGAACAGCCAGTCACGTTTCCG
TTTTTGCCATGGTGATCGGACTTATTGGCTCTATCGCAGCTTGTGTCG
CGTGA

>ARI
ATGTCGGTTTCGCTGCACCACTTCATTATTTCTTCTGGTTTTTTGGCG
AGTATGTTTCCGAAGGCAGTGAGACGCGCCGTCACGGCAGGGGTGTT
TGCCGCGCCCACACTGATGTCGTTCTTGCGATGTGGCGCTATGGCAT
CGGATCCCCCTCTTGTTGCCAATCAAGTTGTCACCTGCCCAGATAAAA
AATCGACAGCCGCGGTCATTCTCACACCGACGGAGAACCACTTCACTC
TCAAGTGCCCTAAAACAGCGCTCACAGAGCCTCCCACTCTTGCGTACT
CACCCAACAGGCAAATCTGCCCAGCGGGTACTACAAGTAGCTGTACATC
AAAGGCTGTAACATTGAGCTCCTTGATTCCTGAAGCAGAAGATAGCTG
GTGGACGGGGGATTCTGCTAGTCTCGACACGGCAGGCATCAAACTCAC
AGTTCCAATCGAGAAGTTCCCCGTGACAACGCAGACGTTTGTGGTCGG
TTGCATCAAGGGAGACGACGCACAGAGTTGTATGGTCACAGTGACAGT
ACAAGCCAGAGCCTCATCGGTCGTCAATAATGTCGCAAGGTGCTCCTA
CGGTGCAAACAGCACTCTTGGTCCTGTCAAGTTGTCTGCGGAAGGAC
CCACTACAATGACCCTCGTGTGCGGGAAAGATGGAGTCAAAGTTCCTC
AAGACAACAATCAGTACTGTTCCGGGACGACGCTGACTGGTTGCAACG
AGAAATCGTTCAAAGATATTTTGCCAAAATTAAGTGAGAACCCGTGGC
AGGGTAACGCTTCGAGTGATAATGGTGCCACGCTAACGATCAACAAGG
AAGCATTTCCAGCCGAGTCAAAAAGCGTCATTATTGGATGCACAGGGG
GATCGCCTGAGAAGCATCACTGTACCGTGCAACTGGAGTTTGCCGGG
GCTGCAGGGTCAGCAAAATCGTCTGCGGGAACAGCCAGTCACGTTTCC
ATTTTCGCCATGGTGACCGGACTTATTGGCTCTATCGCAGCTTGTGTC

GCGTGA

>TgCtPRC2
TGTCGGTTTCGCTGCACCACTTCATTATTTCTTCTGGTTTTTGGCGA
GTATGTTTCCGAAGGCAGTGAGACGCGCCGTCACGGCAGGGGTGTTT
GCCGCGCCCACACTGATGTCGTTCTTGCGATGTGGCGCTATGGCATC
GGATCCCCCTCTTGTTGCCAATCAAGTTGTCACCTGCCCAGATAAAAA
ATCGACAGCCGCGGTCATTCTCACACCGACGGAGAACCACTTCACTCT
CAAGTGCCCTAAAACAGCGCTCACAGAGCCTCCCACTCTTGCGTACTC
ACCCAACAGGCAAATCTGCCCAGCGGGTACTACAAGTAGCTGTACATCA
AAGGCTGTAACATTGAGCTCCTTGATTCCTGAAGCAGAAGATAGCTGG
TGGACGGGGGATTCTGCTAGTCTCGACACGGCAGGCATCAAACTCACA
GTTCCAATCGAGAAGTTCCCCGTGACAACGCAGACGTTTGTGGTCGGT
TGCATCAAGGGAGACGACGCACAGAGTTGTATGGTCACAGTGACAGTA
CAAGCCAGAGCCTCATCGGTCGTCAATAATGTCGCAAGGTGCTCCTAC
GGTGCAAACAGCACTCTTGGTCCTGTCAAGTTGTCTGCGGAAGGACC
CACTACAATGACCCTCGTGTGCGGGAAAGATGGAGTCAAAGTTCCTCA
AGACAACAATCAGTACTGTTCCGGGACGACGCTGACTGGTTGCAACGA
GAAATCGTTCAAAGATATTTTGCCAAAATTAAGTGAGAACCCGTGGCA
GGGTAACGCTTCGAGTGATAATGGTGCCACGCTAACGATCAACAAGGA
AGCATTTCCAGCCGAGTCAAAAAGCGTCATTATTGGATGCACAGGGGG
ATCGCCTGAGAAGCATCACTGTACCGTGCAACTGGAGTTTGCCGGGG
CTGCAGGGTCAGCAAAATCGTCTGCGGGAACAGCCAGTCACGTTTCCA
TTTTCGCCATGGTGACCGGACTTATTGGTTCTATCGCAGCTTGTGTCG
CGTGA

>GAB2-2007-GAL-DOM2
TGTCGGTTTCGCTGCACCACTTCATTATTTCTTCTGGTTTTTGACGA
GTATGTTTCCGAAGGCAGTGAGACGCGCCGTCACGGCAGGGGTGTTT
GCCGCGCCCACACTGATGTCGTTCTTGCGATGTGGCGTTATGGCATC
GGATCCCCCTCTTGTTGCCAATCAAGTTGTCACCTGCCCAGATAAAAA
ATCGACAGCCGCGGTCATTCTCACACCGACGGAGAACCACTTCACTCT
CAAGTGCCCTAAAACAGCGCTCACAGAGCCTCCCACTCTTGCGTACTC
ACCCAACAGGCAAATCTGCCCAGCGGGTACTACAAGTAGCTGTACATCA
AAGGCTGTAACATTGAGCTCCTTGATTCCTGAAGCAGAAGATAGCTGG
TGGACGGGGGATTCTGAAGTGCCCTAAAACAGCGCTCACAGAGCCTCC

CACTCTTGCGTACTCACCCAACAGGCAAATCTGCCCAGCGGGTACTAC
CTAGTCTCGACACGGCAGGCATCAAACTCACAGTTCCAATCGAGAAGT
TCCCCGTGACAACGCAGACGTTTGTGGTCGGTTGCATCAAGGGAGAC
GACGCACAGAGTTGTATGGTCACAGTGACAGTACAAGCCAGAGCCTCA
TCGGTCGTCAATAATGTCGCAAGGTGCTCCTACGGTGCAGACAGCACT
CTTGGTCCTGTCAAGTTGTCTGCGGAAGGACCCACTACAATGACCCTC
GTGTGCGGGAAAGATGGAGTCAAAGTTCCTCAAGACAACAATCAGTAC
TGTTCCGGGACGACGCTGACTGGTTGCAACGAGAAATCGTTCAAAGAT
ATTTTGCCAAAATTAACTGAGAACCCGTGGCAGGGTAACGCTTCGAGT
GATAAGGGTGCCACGCTAACGATCAAGAAGGAAGCATTTCCAGCCGAG
TCAAAAAGCGTCATTATTGGATGCACAGGGGGATCGCCTGAGAAGCAT
CACTGTACCGTGAAACTGGAGTTTGCCGGGGCTGCAGGGTCAGCAAA
ATCGGCTGCGGGAACAGCCAGTCACGTTTCCATTTTTGCCATGGTGAT
CGGACTTATTGGCTCTATCGCAGCTTGTGTCGCGTGA

>TgCtCo5
TGTCGGTTTCGCTGCACCACTTCATTATTTCTTCTGGTTTTTTGACGA
GTATGTTTCCGAAGGCAGTGAGACGCGCCGTCACGGCAGGGGTGTTT
GCCGCGCCCACACTGATGTCGTTCTTGCGATGTGGCGTTATGGCATC
GGATCCCCCTCTTGTTGCCAATCAAGTTGTCACCTGCCCAGATAAAAA
ATCGACAGCCGCGGTCATTCTCACACCGACGGAGAACCACTTCACTCT
CAAGTGCCCTAAAACAGCGCTCACAGAGCCTCCCACTCTTGCGTACTC
ACCCAACAGGCAAATCTGCCCAGCGGGTACTACAAGTAGCTGTACATCA
AAGGCTGTAACATTGAGCTCCTTGATTCCTGAAGCAGAAGATAGCTGG
TGGACGGGGGATTCTGCTAGTCTCGACACGGCAGGCATCAAACTCACA
GTTCCAATCGAGAAGTTCCCCGTGACAACGCAGACGTTTGTGGTCGGT
TGCATCAAGGGAGACGACGCACAGAGTTGTATGGTCACAGTGACAGTA
CAAGCCAGAGCCTCATCGGTCGTCAATAATGTCGCAAGGTGCTCCTAC
GGTGCAGACAGCACTCTTGGTCCTGTCAAGTTGTCTGCGGAAGGACC
CACTACAATGACCCTCGTGTGCGGGAAAGATGGAGTCAAAGTTCCTCA
AGACAACAATCAGTACTGTTCCGGGACGACGCTGACTGGTTGCAACGA
GAAATCGTTCAAAGATATTTTGCCAAAATTAACTGAGAACCCGTGGCA
GGGTAACGCTTCGAGTGATAAGGGTGCCACGCTAACGATCAAGAAGGA
AGCATTTCCAGCCGAGTCAAAAAGCGTCATTATTGGATGCACAGGGGG
ATCGCCTGAGAAGCATCACTGTACCGTGAAACTGGAGTTTGCCGGGGC
TGCAGGGTCAGCAAAATCGGCTGCGGGAACAGCCAGTCACGTTTCCAT

TTTTGCCATGGTGATCGGACTTATTGGCTCTATCGCAGCTTGTGTCGC
GTGA

>TgCatJpOk1
TGTCGGTTTCGCTGCACCACTTCATTATTTCTTCTGGTTTTTGACGA
GTATGTTTCCGAAGGCAGTGAGACGCGCCGTCACGGCAGGGGTGTTT
GCCGCGCCCACACTGATGTCGTTCTTGCGATGTGGCGTTATGGCATC
GGATCCCCCTCTTGTTGCCAATCAAGTTGTCACCTGCCCAGATAAAAA
ATCGACAGCCGCGGTCATTCTCACACCGACGGAGAACCACTTCACTCT
CAAGTGCCCTAAAACAGCGCTCACAGAGCCTCCCACTCTTGCGTACTC
ACCCAACAGGCAAATCTGCCCAGCGGGTACTACAAGTAGCTGTACATCA
AAGGCTGTAACATTGAGCTCCTTGATTCCTGAAGCAGAAGATAGCTGG
TGGACGGGGGATTCTGCTAGTCTCGACACGGCAGGCATCAAACTCACA
GTTCCAATCGAGAAGTTCCCCGTGACAACGCAGACGTTTGTGGTCGGT
TGCATCAAGGGAGACGACGCACAGAGTTGTATGGTCACAGTGACAGTA
CAAGCCAGAGCCTCATCGGTCGTCAATAATGTCGCAAGGTGCTCCTAC
GGTGCAGACAGCACTCTTGGTCCTGTCAAGTTGTCTGCGGAAGGACC
CACTACAATGACCCTCGTGTGCGGGAAAGATGGAGTCAAAGTTCCTCA
AGACAACAATCAGTACTGTTCCGGGACGACGCTGACTGGTTGCAACGA
GAAATCGTTCAAAGATATTTTGCCAAAATTAACTGAGAACCCGTGGCA
GGGTAACGCTTCGAGTGATAAGGGTGCCACGCTAACGATCAAGAAGGA
AGCATTTCCAGCCGAGTCAAAAAGCGTCATTATTGGATGCACAGGGGG
ATCGCCTGAGAAGCATCACTGTACCGTGAAACTGGAGTTTGCCGGGGC
TGCAGGGTCAGCAAAATCGGCTGCGGGAACAGCCAGTCACGTTTCCAT
TTTTGCCATGGTGATCGGACTTATTGGCTCTATCGCAGCTTGTGTCGC
GTGA

>TgCatJpOk2
TGTCGGTTTCGCTGCACCACTTCATTATTTCTTCTGGTTTTTGACGA
GTATGTTTCCGAAGGCAGTGAGACGCGCCGTCACGGCAGGGGTGTTT
GCCGCGCCCACACTGATGTCGTTCTTGCGATGTGGCGTTATGGCATC
GGATCCCCCTCTTGTTGCCAATCAAGTTGTCACCTGCCCAGATAAAAA
ATCGACAGCCGCGGTCATTCTCACACCGACGGAGAACCACTTCACTCT
CAAGTGCCCTAAAACAGCGCTCACAGAGCCTCCCACTCTTGCGTACTC
ACCCAACAGGCAAATCTGCCCAGCGGGTACTACAAGTAGCTGTACATCA
AAGGCTGTAACATTGAGCTCCTTGATTCCTGAAGCAGAAGATAGCTGG

TGGACGGGGGATTCTGCTAGTCTCGACACGGCAGGCATCAAACTCACA
GTTCCAATCGAGAAGTTCCCCGTGACAACGCAGACGTTTGTGGTCGGT
TGCATCAAGGGAGACGACGCACAGAGTTGTATGGTCACAGTGACAGTA
CAAGCCAGAGCCTCATCGGTCGTCAATAATGTCGCAAGGTGCTCCTAC
GGTGCAGACAGCACTCTTGGTCCTGTCAAGTTGTCTGCGGAAGGACC
CACTACAATGACCCTCGTGTGCGGGAAAGATGGAGTCAAAGTTCCTCA
AGACAACAATCAGTACTGTTCCGGGACGACGCTGACTGGTTGCAACGA
GAAATCGTTCAAAGATATTTGCCAAAATTAACTGAGACCCCGTGGCA
GGGTAACGCTTCGAGTGGATAAGGGTGCCACGCTAACGATCAAGAAGG
AAGCATTTCCAGCCGAGTCAAAAAGCGTCATTATTGGATGCACAGGGG
GATCGCCTGAGAAGCATCACTGTACCGTGAAACTGGAGTTTGCCGGG
GCTGCAGGGTCAGCAAAATCGGCTGCGGGAACAGCCAGTCACGTTTC
CATTTTTGCCATGGTGATCGGACTTATTGGCTCTATCGCAGCTTGTGT
CGCGTGA

>TgCatJpOk3
TGTCGGTTTCGCTGCACCACTTCATTATTTCTTCTGGTTTTTTGGCGA
GTATGTTTCCGAAGGCAGTGAGACGCGCCGTCACGGCAGGGGTGTTT
GCCGCGCCCACACTGATGTCGTTCTTGCGATGTGGCGCTATGGCATC
GGATCCCCCTCTTGTTGCCAATCAAGTTGTCACCTGCCCAGATAAAAA
ATCGACAGCCGCGGTCATTCTCACACCGACGGAGAACCACTTCACTCT
CAAGTGCCCTAAAACAGCGCTCACAGAGCCTCCCACTCTTGCGTACTC
ACCCAACAGGCAAATCTGCCCAGCGGGTACTACAAGTAGCTGTACATCA
AAGGCTGTAACATTGAGCTCCTTGATTCCTGAAGCAGAAGATAGCTGG
TGGACGGGGGATTCTGCTAGTCTCGACACGGCAGGCATCAAACTCACA
GTTCCAATCGAGAAGTTCCCCGTGACAACGCAGACGTTTGTGGTCGGT
TGCATCAAGGGAGACGACGCACAGAGTTGTATGGTCACAGTGACAGTA
CAAGCCAGAGCCTCATCGGTCGTCAATAATGTCGCAAGGTGCTCCTAC
GGTGCAAACAGCACTCTTGGTCCTGTCAAGTTGTCTGCGGAAGGACC
CACTACAATGACCCTCGTGTGCGGGAAAGATGGAGTCAAAGTTCCTCA
AGACAACAATCAGTACTGTTCCGGGACGACGCTGACTGGTTGCAACGA
GAAATCGTTCAAAGATATTTGCCAAAATTAAGTGAGAACCCGTGGCA
GGGTAACGCTTCGAGTGATAATGGTGCCACGCTAACGATCAACAAGGA
AGCATTTCCAGCCGAGTCAAAAAGCGTCATTATTGGATGCACAGGGGG
ATCGCCTGAGAAGCATCACTGTACCGTGCAACTGGAGTTTGCCGGGG
CTGCAGGGTCAGCAAAATCGTCTGCGGGAACAGCCAGTCACGTTTCCA

TTTTCGCCATGGTGACCGGACTTATTGGCTCTATCGCAGCTTGTGTCG
CGTGA

>TgCatJpOk4
TGTCGGTTTCGCTGCACCACTTCATTATTTCTTCTGGTTTTTGGCGA
GTATGTTTCCGAAGGCAGTGAGACGCGCCGTCACGGCAGGGGTGTTT
GCCGCGCCCACACTGATGTCGTTCTTGCGATGTGGCGCTATGGCATC
GGATCCCCCTCTTGTTGCCAATCAAGTTGTCACCTGCCCAGATAAAAA
ATCGACAGCCGCGGTCATTCTCACACCGACGGAGAACCACTTCACTCT
CAAGTGCCCTAAAACAGCGCTCACAGAGCCTCCCACTCTTGCGTACTC
ACCCAACAGGCAAATCTGCCCAGCGGGTACTACAAGTAGCTGTACATCA
AAGGCTGTAACATTGAGCTCCTTGATTCCTGAAGCAGAAGATAGCTGG
TGGACGGGGGATTCTGCTAGTCTCGACACGGCAGGCATCAAACTCACA
GTTCCAATCGAGAAGTTCCCCGTGACAACGCAGACGTTTGTGGTCGGT
TGCATCAAGGGAGACGACGCACAGAGTTGTATGGTCACAGTGACAGTA
CAAGCCAGAGCCTCATCGGTCGTCAATAATGTCGCAAGGTGCTCCTAC
GGTGCAAACAGCACTCTTGGTCCTGTCAAGTTGTCTGCGGAAGGACC
CACTACAATGACCCTCGTGTGCGGGAAAGATGGAGTCAAAGTTCCTCA
AGACAACAATCAGTACTGTTCCGGGACGACGCTGACTGGTTGCAACGA
GAAATCGTTCAAAGATATTTTGCCAAAATTAAGTGAGACCCCGTGGCA
GGGTAACGCTTCGAGTGATAATGGTGCCACGCTAACGATCAACAAGGA
AGCATTTCCAGCCGAGTCAAAAAGCGTCATTATTGGATGCACAGGGGG
ATCGCCTGAGAAGCATCACTGTACCGTGCAACTGGAGTTTGCCGGGG
CTGCAGGGTCAGCAAAATCGTCTGCGGGAACAGCCAGTCACGTTTCCA
TTTTCGCCATGGTGACCGGACTTATTGGCTCTATCGCAGCTTGTGTCG
CGTGA

>TgCatJpTy1/k-3
GGTTGTATGTCGGTTTCGCTGCACCACTTCATTATTTCTTCTGGTTTT
TTGGCGAGTATGTTTCCGAAGGCAGTGAGACGCGCCGTCACGGCAGG
GGTGTTTGCCGCGCCCACACTGATGTCGTTCTTGCGATGTGGCGCTA
TGGCATCGGATCCCCCTCTTGTTGCCAATCAAGTTGTCACCTGCCCAG
ATAAAAAATCGACAGCCGCGGTCATTCTCACACCGACGGAGAACCACT
TCACTCTCAAGTGCCCTAAAACAGCGCTCACAGAGCCTCCCACTCTTG
CGTACTCACCCAACAGGCAAATCTGCCCAGCGGGTACTACAAGTAGCT
GTACATCAAAGGCTGTAACATTGAGCTCCTTGATTCCTGAAGCAGAAG

ATAGCTGGTGGACGGGGGATTCTGCTAGTCTCGACACGGCAGGCATC
AAACTCACAGTTCCAATCGAGAAGTTCCCCGTGACAACGCAGACGTTT
GTGGTCGGTTGCATCAAGGGAGACGACGCACAGAGTTGTATGGTCACA
GTGACAGTACAAGCCAGAGCCTCATCGGTCGTCAATAATGTCGCAAGG
TGCTCCTACGGTGCAAACAGCACTCTTGGTCCTGTCAAGTTGTCTGCG
GAAGGACCCACTACAATGACCCTCGTGTGCGGGAAAGATGGAGTCAAA
GTTCCTCAAGACAACAATCAGTACTGTTCCGGGACGACGCTGACTGGT
TGCAACGAGAAATCGTTCAAAGATATTTTGCCAAAATTAAGTGAGAACC
CGTGGCAGGGTAACGCTTCGAGTGATAATGGTGCCACGCTAACGATCA
ACAAGGAAGCATTTCCAGCCGAGTCAAAAAGCGTCATTATTGGATGCA
CAGGGGGATCGCCTGAGAAGCATCACTGTACCGTGCAACTGGAGTTT
GCCGGGGCTGCAGGGTCAGCAAAATCGTCTGCGGGAACAGCCAGTCA
CGTTTCCATTTTCGCCATGGTGACCGGACTTATTGGCTCTATCGCAGC
TTGTGTCGCGTGAGTGATCACCGTTGTGCTCACTTCTCAAATCGACAA
AGGAAACAC

>TgCatJpGi1/TaJ
TGTCGGTTTCGCTGCACCACTTCATTATTTCTTCTGGTTTTTTGGCGA
GTATGTTTCCGAAGGCAGTGAGACGCGCCGTCACGGCAGGGGTGTTT
GCCGCGCCCACACTGATGTCGTTCTTGCGATGTGGCGCTATGGCATC
GGATCCCCCTCTTGTTGCCAATCAAGTTGTCACCTGCCCAGATAAAAA
ATCGACAGCCGCGGTCATTCTCACACCGACGGAGAACCACTTCACTCT
CAAGTGCCCTAAAACAGCGCTCACAGAGCCTCCCACTCTTGCGTACTC
ACCCAACAGGCAAATCTGCCCAGCGGGTACTACAAGTAGCTGTACATCA
AAGGCTGTAACATTGAGCTCCTTGATTCCTGAAGCAGAAGATAGCTGG
TGGACGGGGGATTCTGCTAGTCTCGACACGGCAGGCATCAAACTCACA
GTTCCAATCGAGAAGTTCCCCGTGACAACGCAGACGTTTGTGGTCGGT
TGCATCAAGGGAGACGACGCACAGAGTTGTATGGTCACAGTGACAGTA
CAAGCCAGAGCCTCATCGGTCGTCAATAATGTCGCAAGGTGCTCCTAC
GGTGCAAACAGCACTCTTGGTCCTGTCAAGTTGTCTGCGGAAGGACC
CACTACAATGACCCTCGTGTGCGGGAAAGATGGAGTCAAAGTTCCTCA
AGACAACAATCAGTACTGTTCCGGGACGACGCTGACTGGTTGCAACGA
GAAATCGTTCAAAGATATTTTGCCAAAATTAAGTGAGAACCCGTGGCA
GGGTAACGCTTCGAGTGATAATGGTGCCACGCTAACGATCAACAAGGA
AGCATTTCCAGCCGAGTCAAAAAGCGTCATTATTGGATGCACAGGGGG
ATCGCCTGAGAAGCATCACTGTACCGTGCAACTGGAGTTTGCCGGGG

CTGCAGGGTCAGCAAAATCGTCTGCGGGAACAGCCAGTCACGTTTCCA
TTTTCGCCATGGTGACCGGACTTATTGGCTCTATCGCAGCTTGTGTCG
CGTGA

>TgGoatJpOk1
TGTCGGTTTCGCTGCACCACTTCATTATTTCTTCTGGTTTTTTGACGA
GTATGTTTCCGAAGGCAGTGAGACGCGCCGTCACGGCAGGGGTGTTT
GCCGCGCCCACACTGATGTCGTTCTTGCGATGTGGCGTTATGGCATC
GGATCCCCCTCTTGTTGCCAATCAAGTTGTCACCTGCCCAGATAAAAA
ATCGACAGCCGCGGTCATTCTCACACCGACGGAGAACCACTTCACTCT
CAAGTGCCCTAAAACAGCGCTCACAGAGCCTCCCACTCTTGCGTACTC
ACCCAACAGGCAAATCTGCCCAGCGGGTACTACAAGTAGCTGTACATCA
AAGGCTGTAACATTGAGCTCCTTGATTCCTGAAGCAGAAGATAGCTGG
TGGACGGGGGATTCTGCTAGTCTCGACACGGCAGGCATCAAACTCACA
GTTCCAATCGAGAAGTTCCCCGTGACAACGCAGACGTTTGTGGTCGGT
TGCATCAAGGGAGACGACGCACAGAGTTGTATGGTCACAGTGACAGTA
CAAGCCAGAGCCTCATCGGTCGTCAATAATGTCGCAAGGTGCTCCTAC
GGTGCAGACAGCACTCTTGGTCCTGTCAAGTTGTCTGCGGAAGGACC
CACTACAATGACCCTTCGTGTGCGGGAAAGATGGAGTCAAAGTTCCTC
AAGACAACAATCAGTACTGTTCCGGGACGACGCTGACTGGTTGCAACG
AGAAATCGTTCAAAGATATTTTGCCAAAATTAACTGAGAACCCGTGGCA
GGGTAACGCTTCGAGTGATAAGGGTGCCACGCTAACGATCAAGAAGGA
AGCATTTCCAGCCGAGTCAAAAAGCGTCATTATTGGATGCACAGGGGG
ATCGCCTGAGAAGCATCACTGTACCGTGAAACTGGAGTTTGCCGGGGC
TGCAGGGTCAGCAAAATCGGCTGCGGGAACAGCCAGTCACGTTTCCAT
TTTTGCCATGGTGATCGGACTTATTGGCTCTATCGCAGCTTGTGTCGC
GTGA

>TgGoatJpOk2
CGTGTAAACACACGGTTGTATGTCGGTTTCGCTGCACCACTTCATTAT
TTCTTCTGGTTTTTTGACGAGTATGTTTCCGAAGGCAGTGAGACGCGC
CGTCACGGCAGGGGTGTTTGCCGCGCCCACACTGATGTCGTTCTTGC
GATGTGGCGTTATGGCATCGGATCCCCCTCTTGTTGCCAATCAAGTTG
TCACCTGCCCAGATAAAAAATCGACAGCCGCGGTCATTCTCACACCGA
CGGAGAACCACTTCACTCTCAAGTGCCCTAAAACAGCGCTCACAGAGC
CTCCCACTCTTGCGTACTCACCCAACAGGCAAATCTGCCCAGCGGGTA

CTACAAGTAGCTGTACATCAAAGGCTGTAACATTGAGCTCCTTGATTCC
TGAAGCAGAAGATAGCTGGTGGACGGGGGATTCTGCTAGTCTCGACAC
GGCAGGCATCAAACTCACAGTTCCAATCGAGAAGTTCCCCGTGACAAC
GCAGACGTTTGTGGTCGGTTGCATCAAGGGAGACGACGCACAGAGTT
GTATGGTCACAGTGACAGTACAAGCCAGAGCCTCATCGGTCGTCAATA
ATGTCGCAAGGTGCTCCTACGGTGCAGACAGCACTCTTGGTCCTGTCA
AGTTGTCTGCGGAAGGACCCACTACAATGACCCTCGTGTGCGGGAAAG
ATGGAGTCAAAGTTCCTCAAGACAACAATCAGTACTGTTCCGGGACGA
CGCTGACTGGTTGCAACGAGAAATCGTTCAAAGATATTTTGCCAAAAT
TAACTGAGAACCCGTGGCAGGGTAACGCTTCGAGTGATAAGGGTGCCA
CGCTAACGATCAAGAAGGAAGCATTTCCAGCCGAGTCAAAAAGCGTCA
TTATTGGATGCACAGGGGGATCGCCTGAGAAGCATCACTGTACCGTGA
AACTGGAGTTTGCCGGGGCTGCAGGGTCAGCAAAATCGGCTGCGGGA
ACAGCCAGTCACGTTTCCATTTTTGCCATGGTGATCGGACTTATTGGC
TCTATCGCAGCTTGTGTCGCGTGAGTGATCACCGTTGTGCTCACTTCT
CAAATC

>TgGoatJpOk3
TTCACATGTGTCATTGTCGTGTAAACACACGGTTGTATGTCGGTTTCG
CTGCACCACTTCATTATTTCTTCTGGTTTTTGGCGAGTATGTTTCCG
AAGGCAGTGAGACGCGCCGTCACGGCAGGGGTGTTTGCCGCGCCCAC
ACTGATGTCGTTCTTGCGATGTGGCGCTATGGCATCGGATCCCCCTCT
TGTTGCCAATCAAGTTGTCACCTGCCCAGATAAAAAATCGACAGCCGC
GGTCATTCTCACACCGACGGAGAACCACTTCACTCTCAAGTGCCCTAA
AACAGCGCTCACAGAGCCTCCCACTCTTGCGTACTCACCCAACAGGCA
AATCTGCCCAGCGGGTACTACAAGTAGCTGTACATCAAAGGCTGTAAC
ATTGAGCTCCTTGATTCCTGAAGCAGAAGATAGCTGGTGGACGGGGG
ATTCTGCTAGTCTCGACACGGCAGGCATCAAACTCACAGTTCCAATCG
AGAAGTTCCCCGTGACAACGCAGACGTTTGTGGTCGGTTGCATCAAGG
GAGACGACGCACAGAGTTGTATGGTCACAGTGACAGTACAAGCCAGAG
CCTCATCGGTCGTCAATAATGTCGCAAGGTGCTCCTACGGTGCAGACA
GCACTCTTGGTCCTGTCAAGTTGTCTGCGGAAGGACCCACTACAATGA
CCCTCGTGTGCGGGAAAGATGGAGTCAAAGTTCCTCAAGACAACAATC
AGTACTGTTCCGGGACGACGCTGACTGGTTGCAACGAGAAATCGTTCA
AAGATATTTTGCCAAAATTAACTGAGAACCCGTGGCAGGGTAACGCTT
CGAGTGATAAGGGTGCCACGCTAACGATCAAGAAGGAAGCATTTCCAG

CCGAGTCAAAAAGCGTCATTATTGGATGCACAGGGGGATCGCCTGAGA
AGCATCACTGTACCGTGAAACTGGAGTTTGCCGGGGCTGCAGGGTCA
GCAAAATCGGCTGCGGGAACAGCCAGTCACGTTTCCATTTTTGCCATG
GTGATCGGACTTATTGGCTCTATCGCAGCTTGTGTCGCGTGAGTGATC
ACCGTTGTGCTCACTTCTCAAATCNACAAAGGGAAACACA

>TgGoatJpOk4
TGTCGGTTTCGCTGCACCACTTCATTATTTCTTCTGGTTTTTGACGA
GTATGTTTCCGAAGGCAGTGAGACGCGCCGTCACGGCAGGGGTGTTT
GCCGCGCCCACACTGATGTCGTTCTTGCGATGTGGCGTTATGGCATC
GGATCCCCCTCTTGTTGCCAATCAAGTTGTCACCTGCCCAGATAAAAA
ATCGACAGCCGCGGTCANTCTCACACCGACGGAGAACCACTTCACTCT
CAAGTGCCCTAAAACAGCGCTCACAGAGCCTCCCATTCTTGCGTACTC
ACCCAACAGGCAAATCTGCCCAGCGGGTACTACAAGTAGCTGTACATCA
AAGGCTGTAACATTGAGCTCCTTGATTCCTGAAGCAGAAGATAGCTGG
TGGACGGGGGATTCTGCTAGTCTCGACACGGCAGGCATCAAACTCACA
GTTCCAATCGAGAAGTTCCCCGTGACAACGCAGACGTTTGTGGTCGGT
TGCATCAAGGGAGACGACGCACAGAGTTGTATGGTCACAGTGACAGTA
CAAGCCAGAGCCTCATCGGTCGTCAATAATGTCGCAAGGTGCTCCTAC
GGTGCAGACAGCACTCTTGGTCCTGTCAAGTTGTCTGCGGAAGGACC
CACTACAATGACCCTCGTGTGCGGGAAAGATGGAGTCAAAGTTCCTCA
AGACAACAATCAGTACTGTTCCGGGACGACGCTGACTGGTTGCAACGA
GAAATCGTTCAAAGATATTTTGCCAAAATTAACTGAGAACCCGTGGCA
GGGTAACGCTTCGAGTGATAAGGGTGCCACGCTAACGATCAAGAAGGA
AGCATTTCCAGCCGAGTCAAAAAGCGTCATTATTGGATGCACAGGGGG
ATCGCCTGAGAAGCATCACTGTACCGTGAAACTGGAGTTTGCCGGGGC
TGCAGGGTCAGCAAAATCGGCTGCGGGAACAGCCAGTCACGTTTCCAT
TTTTGCCATGGTGATCGGACTTATTGGCTCTATCGCAGCTTGTGTCGC
GTGA

>TgGoatJpOk5
TGTCGGTTTCGCTGCACCACTTCATTATTTCTTCTGGTTTTTGACGA
GTATGTTTCCGAAGGCAGTGAGACGCGCCGTCACGGCAGGGGTGTTT
GCCGCGCCCACACTGATGTCGTTCTTGCGATGTGGCGTTATGGCATC
GGATCCCCCTCTTGTTGCCAATCAAGTTGTCACCTGCCCAGATAAAAA
ATCGACAGCCGCGGTCATTCTCACACCGACGGAGAACCACTTCACTCT

CAAGTGCCCTAAAACAGCGCTCACAGAGCCTCCCATTCTTGCGTACTC
ACCCAACAGGCAAATCTGCCCAGCGGGTACTACAAGTAGCTGTACATCA
AAGGCTGTAACATTGAGCTCCTTGATTCCTGAAGCAGAAGATAGCTGG
TGGACGGGGGATTCTGCTAGTCTCGACACGGCAGGCATCAAACTCACA
GTTCCAATCGAGAAGTTCCCCGTGACAACGCAGACGTTTGTGGTCGGT
TGCATCAAGGGAGACGACGCACAGAGTTGTATGGTCACAGTGACAGTA
CAAGCCAGAGCCTCATCGGTCGTCAATAATGTCGCAAGGTGCTCCTAC
GGTGCAGACAGCACTCTTGGTCCTGTCAAGTTGTCTGCGGAAGGACC
CACTACAATGACCCTTCGTGTGGCGGGAAAGATGGAGTCAAAGTTCCT
CAAGACAACAATCAGTACTGTTCCGGGACGACGCTGACTGGTTGCAAC
GAGAAATCGTTCAAAGATATTTTGCCAAAATTAACTGAGAACCCGTGG
CAGGGTAACGCTTCGAGTGATAAGGGTGCCACGCTAACGATCAAGAAG
GAAGCATTTCCAGCCGAGTCAAAAAGCGTCATTATTGGATGCACAGGG
GGATCGCCTGAGAAGCATCACTGTACCGTGAAACTGGAGTTTGCCGG
GGCTGCAGGGTCAGCAAAATCGGCTGCGGGAACAGCCAGTCACGTTT
CCATTTTTGCCATGGTGATCGGACTTATTGGCTCTANCGCAGCTTGTG
TCGCGTGA

>TgGoatJpOk6
TTCACATGTGTCATTGTCGTGTAAACACACGGTTGTATGTCGGTTTCG
CTGCACCACTTCATTATTTCTTCTGGTTTTTTGGCGAGTATGTTTCCG
AAGGCAGTGAGACGCGCCGTCACGGCAGGGGTGTTTGCCGCGCCCAC
ACTGATGTCGTTCTTGCGATGTGGCGCTATGGCATCGGATCCCCCTCT
TGTTGCCAATCAAGTTGTCACCTGCCCAGATAAAAAATCGACAGCCGC
GGTCATTCTCACACCGACGGAGAACCACTTCACTCTCAAGTGCCCTAA
AACAGCGCTCACAGAGCCTCCCACTCTTGCGTACTCACCCAACAGGCA
AATCTGCCCAGCGGGTACTACAAGTAGCTGTACATCAAAGGCTGTAAC
ATTGAGCTCCTTGATTCCTGAAGCAGAAGATAGCTGGTGGACGGGGG
ATTCTGCTAGTCTCGACACGGCAGGCATCAAACTCACAGTTCCAATCG
AGAAGTTCCCCGTGACAACGCAGACGTTTGTGGTCGGTTGCATCAAGG
GAGACGACGCACAGAGTTGTATGGTCACAGTGACAGTACAAGCCAGAG
CCTCATCGGTCGTCAATAATGTCGCAAGGTGCTCCTACGGTGCAAACA
GCACTCTTGGTCCTGTCAAGTTGTCTGCGGAAGGACCCACTACAATGA
CCCTCGTGTGCGGGAAAGATGGAGTCAAAGTTCCTCAAGACAACAATC
AGTACTGTTCCGGGACGACGCTGACTGGTTGCAACGAGAAATCGTTCA
AAGATATTTTGCCAAAATTAAGTGAGAACCCGTGGCAGGGTAACGCTT

CGAGTGATAATGGTGCCACGCTAACGATCAACAAGGAAGCATTTCCAG
CCGAGTCAAAAAGCGTCATTATTGGATGCACAGGGGGATCGCCTGAGA
AGCATCACTGTACCGTGCAACTGGAGTTTGCCGGGGCTGCAGGGTCA
GCAAAATCGTCTGCGGGAACAGCCAGTCACGTTTCCATTTTCGCCATG
GTGACCGGACTTATTGGCTCTATCGCAGCTTGTGTCGCGTGAGTGATC
ACCGTTGTGCTCACTTCTCAAATCGACA

>TgGoatJpOk7
ACATGTGtCATTGTCGTGTAAACACACGGTTGTATGTCGGTTTCGCTG
CACCACTTCATTATTTCTTCTGGTTTTTTGGCGAGTATGTTTCCGAAG
GCAGTGAGACGCGCCGTCACGGCAGGGGTGTTTGCCGCGCCCACACT
GATGTCGTTCTTGCGATGTGGCGCTATGGCATCGGATCCCCCTCTTG
TTGCCAATCAAGTTGTCACCTGCCCAGATAAAAAATCGACAGCCGCGG
TCATTCTCACACCGACGGAGAACCACTTCACTCTCAAGTGCCCTAAAAC
AGCGCTCACAGAGCCTCCCACTCTTGCGTACTCACCCAACAGGCAAAT
CTGCCCAGCGGGTACTACAAGTAGCTGTACATCAAAGGCTGTAACATT
GAGCTCCTTGATTCCTGAAGCAGAAGATAGCTGGTGGACGGGGGATT
CTGCTAGTCTCGACACGGCAGGCATCAAACTCACAGTTCCAATCGAGA
AGTTCCCCGTGACAACGCAGACGTTTGTGGTCGGTTGCATCAAGGGA
GACGACGCACAGAGTTGTATGGTCACAGTGACAGTACAAGCCAGAGCC
TCATCGGTCGTCAATAATGTCGCAAGGTGCTCCTACGGTGCAAACAGC
ACTCTTGGTCCTGTCAAGTTGTCTGCGGAAGGACCCACTACAATGACC
CTCGTGTGCGGGAAAGATGGAGTCAAAGTTCCTCAAGACAACAATCAG
TACTGTTCCGGGACGACGCTGACTGGTTGCAACGAGAAATCGTTCAAA
GATATTTTGCCAAAATTAAGTGAGAACCCGTGGCAGGGTAACGCTTCG
AGTGATAATGGTGCCACGCTAACGATCAACAAGGAAGCATTTCCAGCC
GAGTCAAAAAGCGTCATTATTGGATGCACAGGGGGATCGCCTGAGAAG
CATCACTGTACCGTGCAACTGGAGTTTGCCGGGGCTGCAGGGTCAGC
AAAATCGTCTGCGGGAACAGCCAGTCACGTTTCCATTTTCGCCATGGT
GACCGGACTTATTGGCTCTATCGCAGCTTGTGTCGCGTGAGTGATCAC
CGTTGTGCTCACTTCTCAAATCGACAAAGGAAACAC

>TgGoatJpOk8
GTGTAAACACACGGTTGTATGTCGGTTTCGCTGCACCACTTCATTATT
TCTTCTGGTTTTTTGGCGAGTATGTTTCCGAAGGCAGTGAGACGCGC
CGTCACGGCAGGGGTGTTTGCCGCGCCCACACTGATGTCGTTCTTGC

GATGTGGCGCTATGGCATCGGATCCCCCTCTTGTTGCCAATCAAGTTG
TCACCTGCCCAGATAAAAAATCGACAGCCGCGGTCATTCTCACACCGA
CGGAGAACCACTTCACTCTCAAGTGCCCTAAAACAGCGCTCACAGAGC
CTCCCACTCTTGCGTACTCACCCAACAGGCAAATCTGCCCAGCGGGTA
CTACAAGTAGCTGTACATCAAAGGCTGTAACATTGAGCTCCTTGATTCC
TGAAGCAGAAGATAGCTGGTGGACGGGGGATTCTGCTAGTCTCGACAC
GGCAGGCATCAAACTCACAGTTCCAATCGAGAAGTTCCCCGTGACAAC
GCAGACGTTTGTGGTCGGTTGCATCAAGGGAGACGACGCACAGAGTT
GTATGGTCACAGTGACAGTACAAGCCAGAGCCTCATCGGTCGTCAATA
ATGTCGCAAGGTGCTCCTACGGTGCAAACAGCACTCTTGGTCCTGTCA
AGTTGTCTGCGGAAGGACCCACTACAATGACCCTCGTGTGCGGGAAAG
ATGGAGTCAAAGTTCCTCAAGACAACAATCAGTACTGTTCCGGGACGA
CGCTGACTGGTTGCAACGAGAAATCGTTCAAAGATATTTTGCCAAAAT
TAAGTGAGAACCCGTGGCAGGGTAACGCTTCGAGTGATAATGGTGCCA
CGCTAACGATCAACAAGGAAGCATTTCCAGCCGAGTCAAAAAGCGTCA
TTATTGGATGCACAGGGGGATCGCCTGAGAAGCATCACTGTACCGTGC
AACTGGAGTTTGCCGGGGCTGCAGGGTCAGCAAAATCGTCTGCGGGA
ACAGCCAGTCACGTTTCCATTTTCGCCATGGTGACCGGACTTATTGGC
TCTATCGCAGCTTGTGTCGCGTGAGTGATCACCGTTGTGCTCACTTCT
CAAAT

>TgGoatJpOk9
TTCACATGTGTCATTGTCGTGTAAACACACGGTTGTATGTCGGTTTCG
CTGCACCACTTCATTATTTCTTCTGGTTTTTTGGCGAGTATGTTTCCG
AAGGCAGTGAGACGCGCCGTCACGGCAGGGGTGTTTGCCGCGCCCAC
ACTGATGTCGTTCTTGCGATGTGGCGCTATGGCATCGGATCCCCCTCT
TGTTGCCAATCAAGTTGTCACCTGCCCAGATAAAAAATCGACAGCCGC
GGTCATTCTCACACCGACGGAGAACCACTTCACTCTCAAGTGCCCTAA
AACAGCGCTCACAGAGCCTCCCACTCTTGCGTACTCACCCAACAGGCA
AATCTGCCCAGCGGGTACTACAAGTAGCTGTACATCAAAGGCTGTAAC
ATTGAGCTCCTTGATTCCTGAAGCAGAAGATAGCTGGTGGACGGGGG
ATTCTGCTAGTCTCGACACGGCAGGCATCAAACTCACAGTTCCAATCG
AGAAGTTCCCCGTGACAACGCAGACGTTTGTGGTCGGTTGCATCAAGG
GAGACGACGCACAGAGTTGTATGGTCACAGTGACAGTACAAGCCAGAG
CCTCATCGGTCGTCAATAATGTCGCAAGGTGCTCCTACGGTGCAAACA
GCACTCTTGGTCCTGTCAAGTTGTCTGCGGAAGGACCCACTACAATGA

CCCTCGTGTGCGGGAAAGATGGAGTCAAAGTTCCTCAAGACAACAATC
AGTACTGTTCCGGGACGACGCTGACTGGTTGCAACGAGAAATCGTTCA
AAGATATTTTGCCAAAATTAAGTGAGAACCCGTGGCAGGGTAACGCTT
CGAGTGATAATGGTGCCACGCTAACGATCAACAAGGAAGCATTTCCAG
CCGAGTCAAAAAGCGTCATTATTGGATGCACAGGGGGATCGCCTGAGA
AGCATCACTGTACCGTGCAACTGGAGTTTGCCGGGGCTGCAGGGTCA
GCAAAATCGTCTGCGGGAACAGCCAGTCACGTTTCCATTTTCGCCATG
GTGACCGGACTTATTGGCTCTATCGCAGCTTGTGTCGCGTGAGTGATC
ACCGTTGTGCTCACTTCTCAAATCGACAAAGGAAACAC

>TgGoatJpOk10
TCGTGTAAACACACGGTTGTATGTCGGTTTCGCTGCACCACTTCATTA
TTTCTTCTGGTTTTTTGGCGAGTATGTTTCCGAAGGCAGTGAGACGC
GCCGTCACGGCAGGGGTGTTTGCCGCGCCCACACTGATGTCGTTCTT
GCGATGTGGCGCTATGGCATCGGATCCCCCTCTTGTTGCCAATCAAGT
TGTCACCTGCCCAGATAAAAAATCGACAGCCGCGGTCATTCTCACACC
GACGGAGAACCACTTCACTCTCAAGTGCCCTAAAACAGCGCTCACAGA
GCCTCCCACTCTTGCGTACTCACCCAACAGGCAAATCTGCCCAGCGGG
TACTACAAGTAGCTGTACATCAAAGGCTGTAACATTGAGCTCCTTGATT
CCTGAAGCAGAAGATAGCTGGTGGACGGGGGATTCTGCTAGTCTCGA
CACGGCAGGCATCAAACTCACAGTTCCAATCGAGAAGTTCCCCGTGAC
AACGCAGACGTTTGTGGTCGGTTGCATCAAGGGAGACGACGCACAGA
GTTGTATGGTCACAGTGACAGTACAAGCCAGAGCCTCATCGGTCGTCA
ATAATGTCGCAAGGTGCTCCTACGGTGCAAACAGCACTCTTGGTCCTG
TCAAGTTGTCTGCGGAAGGACCCACTACAATGACCCTCGTGTGCGGGA
AAGATGGAGTCAAAGTTCCTCAAGACAACAATCAGTACTGTTCCGGGA
CGACGCTGACTGGTTGCAACGAGAAATCGTTCAAAGATATTTTGCCAA
AATTAAGTGAGAACCCGTGGCAGGGTAACGCTTCGAGTGATAATGGTG
CCACGCTAACGATCAACAAGGAAGCATTTCCAGCCGAGTCAAAAAGCG
TCATTATTGGATGCACAGGGGGATCGCCTGAGAAGCATCACTGTACCG
TGCAACTGGAGTTTGCCGGGGCTGCAGGGTCAGCAAAATCGTCTGCG
GGAACAGCCAGTCACGTTTCCATTTTCGCCATGGTGACCGGACTTATT
GGCTCTATCGCAGCTTGTGTCGCGTGAGTGATCACCGTTGTGCTCACT
TCTCAAA

>TgGoatJpOk11

GTCGTGTAAACACACGGTTGTATGTCGGTTTCGCTGCACCACTTCATT
ATTTCTTCTGGTTTTTTGGCGAGTATGTTTCCGAAGGCAGTGAGACGC
GCCGTCACGGCAGGGGTGTTTGCCGCGCCCACACTGATGTCGTTCTT
GCGATGTGGCGCTATGGCATCGGATCCCCCTCTTGTTGCCAATCAAGT
TGTCACCTGCCCAGATAAAAAATCGACAGCCGCGGTCATTCTCACACC
GACGGAGAACCACTTCACTCTCAAGTGCCCTAAAACAGCGCTCACAGA
GCCTCCCACTCTTGCGTACTCACCCAACAGGCAAATCTGCCCAGCGGG
TACTACAAGTAGCTGTACATCAAAGGCTGTAACATTGAGCTCCTTGATT
CCTGAAGCAGAAGATAGCTGGTGGACGGGGGATTCTGCTAGTCTCGA
CACGGCAGGCATCAAACTCACAGTTCCAATCGAGAAGTTCCCCGTGAC
AACGCAGACGTTTGTGGTCGGTTGCATCAAGGGAGACGACGCACAGA
GTTGTATGGTCACAGTGACAGTACAAGCCAGAGCCTCATCGGTCGTCA
ATAATGTCGCAAGGTGCTCCTACGGTGCAAACAGCACTCTTGGTCCTG
TCAAGTTGTCTGCGGAAGGACCCACTACAATGACCCTCGTGTGCGGGA
AAGATGGAGTCAAAGTTCCTCAAGACAACAATCAGTACTGTTCCGGGA
CGACGCTGACTGGTTGCAACGAGAAATCGTTCAAAGATATTTTGCCAA
AATTAAGTGAGAACCCGTGGCAGGGTAACGCTTCGAGTGATAATGGTG
CCACGCTAACGATCAACAAGGAAGCATTTCCAGCCGAGTCAAAAAGCG
TCATTATTGGATGCACAGGGGGATCGCCTGAGAAGCATCACTGTACCG
TGCAACTGGAGTTTGCCGGGGCTGCAGGGTCAGCAAAATCGTCTGCG
GGAACAGCCAGTCACGTTTCCATTTTCGCCATGGTGACCGGACTTATT
GGCTCTATCGCAGCTTGTGTCGCGTGAGTGATCACCGTTGTGCTCACT
TCTCAAATCGACAAA

Hypothetical protein intron 2 (HP2)

>GT1
CCCTTCCTGTTTGTTTCTGCAGGTACGACCAGAAAGGCGTAGTGTACT
CCAATAAAATCCAGAAAGGCAGGGCAACGTGTCTGCACGCCACGAACC
ACGCAGCTTCCTCAACCGCCTGCTGAGCACGCAACCCTGGACCCCCTT
CAGGCGATAGCGGAATGAGCAATTGTGCACCTGGGGCAGAAACAGTTC
TTACAGGCAAGGGAACCGGACAAAGCAGGCAAGAGACTGAGGTGAGAA
AACAGTGACACAAGAACTCAGCGGATCTCGTATTCATTGAACCTAGAAG
CTGCTCGTTGACCCACGTTTTCCGACCGAAGGCACCGGCCCTCTGCTC
ATCTAGCGACAACTGTTGAAGGCTATGGTGTACAATCGTTCGCAACCA
TAAAAAACAGTTCGGACGGGCTTGAGCCTATCCCCCTCGAGGTAGAAA
GGCGACACTCTCTTCCAACTGACGAATGGATCCAAAA

>ME49
GACAGAAACACGCAGAGAATATGTCACCGATTACACACCCGGAAATGC
GAAAAAATGAGCAGAACACGATGAGCACAAGAAGAGCAAACGAAACTAA
CCGGAAACAGGAAACAGTTGACTCTCCCCTTCCGCGCTAGGCACGCAC
CCCGTGAGTTAACAAAGATGGAAAGATTGATGAAACAGAAAGAATGAT
GTGGCGTGAGCCCGCAAAAGTGACTATAACACGAACCGTAAAGACTCG
GCAAAAAGTGAAGGCAAACGAGGCACACCACAACCAGCGAAGCTGCAG
GAATAATACAGTCAGTTCCCTCGATCGCAGCACTCGAATACAGCTGCC
GAAAAGCATACTGCCCTTCCTGTTTGTTTCTGCAGGTACGACCAGAAA
GGCGTAGTGTACTCCAATAAAATCCAGAAAGGCAGGGCAACGTGTCTG
CACGCCACGAACCACGCAGCTTCCTCAACCGCCTGCTGAGCACGCAAC
CCTGGACCCCCTTCAGGCGATAGCGGAATGAGCAATTGTGCACCTGG
GGCAGAAACAGTTCTTACAGGCAAGGGAACCGGACAAAGCAGGCAAGA
GACTGAGGTGAGAAAACAGTGACACAAGAACTCAGCGGATCTCGTATT
CATTGAACCTAGAAGCTGCTCGTTGACCCACGTTTTCCGACCGAAGGC
ACCGGCCCTCTGCTCATCTAGCGACAACTGTTGAAGGCTATGGTGTAC
AATCGTTCGCAACCATAAAAAACAGTTCGGACGGGCTTGAGCCTATCC
CCCTCGAGGTAGAAAGGCGACACTCTCTTCCAACTGACGAATGGATCC
AAAACGCAGCAAGCATGGGAACAAAGATTA

>VEG
CCCTTCCTGTTTGTTTCTGCAGGTACGACCAGAAAGGCGTAGTGTACT

CCAATAAAGTCCAGAAAGGCAGGGCAACGTGTCTGCACGCCACGAACC
ACGCAGCTTCCTCAACCGCCTGCTGAGCACGCAACCCTGGACCCCCTT
CAGGCGATAGCGGAATGAGCAATTGTGCACCTGGGGTATAAACAGTTC
TTACAGGCAAGGGAACCGGACAAAGCAGGCAAGAGACTGAGGTGAGAA
AACAGTGACACAAGAACTCAGCGGATCTCATATTCATTGAACCTAGAAG
CTGCTCGTTGACCCACGTTTTCCGACCGAAGGCACCGGCCCTCTGCTC
ATCTAGCGACAACTGTTGAAGGCTATGGTGTACAATCGTTCGCAACCA
TAAAAAACAGTTCGGACGGGCTTGAGCCTATCCCCCTCGAGGTAGAAA
GGCGACACTCTCTTCCAACTGACGAATGGATCCAAAA

>MAS
CCCTTCCTGTTTGTTTCTGCAGGTACGACCAGAAAGGCGTAGTGTACT
CCAACAAAATCCAGAAAGGCGGGGCAACGTGTCTGCACGCTACGAACC
ACGCAGCTTCCTCAACCGCCTGCTGAGCACGCAACCCTGGACCCCCTT
CAGGCGATAGCGGAATGAGCAATTGTGCACCTGGGGCATAAACAGTTC
TTACAGGCAAGGGAACCGGACAAAGCAGGCAAGAGACTGAGGTGAGAA
AACAGTGACACAAGAACTCAGCGGATCTCATATTCATTGAACCTAGAAG
CTGCTCGTTGACCCACGTTTTCCGACCGAAGGCACCGGCCCTCTGCTC
ATCTAGCGACAACTGTTGAAGGCTATGGTGTACAATCGTTCGCAACCA
TAAAAAACAGTTCGGACGGGCTTGAGCCTATCCCCCTCCAGGTAGAAA
GGCGACACTCTCTTCCAACTGACGAATGGATCCAAAG

>RUB
CCCTTCCTGTTTGTTTCTGCAGGTACGACCAGAAAGGCGTAGTGTACT
CCAATAAAATCCAGAAAGGCAGGGCAACGTGTCTGCACGCCACGAACC
ACGCAGCTTCCTCAACCGCCTGCTGAGCACGCAACCCTGGACCCCCTT
CAGGCGATAGCGGAATGAGCAATTGTGCACCTGGGGTATAAACAGTTC
TTACAGGCAAGGGAACCGGACAAAGCAGGCAAGAGACTGAGGTGAGAA
AACAGTGACACAAGAACTCAGCGGATCTCATATTCATTGAACCTAGAAG
CTGCTCGTTGACCCACGTTTTCCGACCGAAGGCACCGGCCCTCTGCTC
ATCTAGCGACAACTGTTGAAGGCTATGGTGTACAATCGTTCGCAACCA
TAAAAAACAGTTCGGACGGGCTTGAGCCTATCCCCCTCGAGGTAGAAG
GGCGACACTCTCTTCCAACTGACGAATGGATCCAAAA

>FOU
CCCTTCCTGTTTGTTTCTGCAGGTACGACCAGAAAGGCGTAGTGTACT

CCAATAAAATCCAGAAAGGCAGGGCAACGTGTCTGCACGCCACGAACC
ACGCAGCTTCCTCAACCGCCTGCTGAGCACGCAACCCTGGACCCCCTT
CAGGCGATAGCGGAATGAGCAATTGTGCACCTGGGGTATAAACAGTTC
TTACAGGCAAGGGAACCGGACAAAGCAGGCAAGAGACTGAGGTGAGAA
AACAGTGACACAAGAACTCAGCGGATCTCATATTCATTGAACCTAGAAG
CTGCTCGTTGACCCACGTTTTCCGACCGAAGGCACCGGCCCTCTGCTC
ATCTAGCGACAACTGTTGAAGGCTATGGTGTACAATCGTTCGCAACCA
TAAAAAACAGTTCGGACGGGCTTGAGCCTATCCCCCTCGAGGTAGAAA
GGCGACACTCTCTTCCAACTGACGAATGGATCCAAAA

>CAST
CCCTTCCTGTTTGTTTCTGCAGGTACGACCAGAAAGGCGTAGTGTACT
CCAATAAAATCCAGAAAGGCAGGGCAACGTGTCTGCACGCCACGAACC
ACGCAGCTTCCTCAACCGCCTGCTGAGCACGCAACCCTGGACCCCCTT
CAGGCGATAGCGGAATGAGCAATTGTGCACCTGGGGTATAAACAGTTC
TTACAGGCAAGGGAACCGGACAAAGCAGGCAAGAGACTGAGGTGAGAA
AACAGTGACACAAGAACTCAGCGGATCTCATATTCATTGAACCTAGAAG
CTGCTCGTTGACCCACGTTTTCCGACCGAAGGCACCGGCCCTCTGCTC
ATCTAGCGACAACTGTTGAAGGCTATGGTGTACAATCGTTCGCAACCA
TAAAAAACAGTTCGGACGGGCTTGAGCCTATCCCCCTCGAGGTAGAAA
GGCGACACTCTCTTCCAACTGACGAATGGATCCAAAA

>TgCatBr5
CCCTTCCTGTTTGTTTCTGCAGGTACGACCAGAAAGGCGTAGTGTACT
CCAATAAAGTCCAGAAAGGCAGGGCAACGTGTCTGCACGCCACGAACC
ACGCAGCTTCCTCAACCGCCTGCTGAGCACGCAACCCTGGACCCCCTT
CAGGCGATAGCGGAATGAGCAATTGTGCACCTGGGGTATAAACAGTTC
TTACAGGCAAGGGAACCGGACAAAGCAGGCAAGAGACTGAGGTGAGAA
AACAGTGACACAAGAACTCAGCGGATCTCATATTCATTGAACCTAGAAG
CTGCTCGTTGACCCACGTTTTCCGACCGAAGGCACCGGCCCTCTGCTC
ATCTAGCGACAACTGTTGAAGGCTATGGTGTACAATCGTTCGCAACCA
TAAAAAACAGTTCGGACGGGCTTGAGCCTATCCCCCTCGAGGTAGAAA
GGCGACACTCTCTTCCAACTGACGAATGGATCCAAAA

>p89
CCCTTCCTGTTTGTTTCTGCAGGTACGACCAGAAAGGCGTAGTGTACT

CCAATAAAGTCCAGAAAGGCAGGGCAACGTGTCTGCACGCCACGAACC
ACGCAGCTTCCTCAACCGCCTGCTGAGCACGCAACCCTGGACCCCCTT
CAGGCGATAGCGGAATGAGCAATTGTGCACCTGGGGTATAAACAGTTC
TTACAGGCAAGGGAACCGGACAAAGCAGGCAAGAGACTGAGGTGAGAA
AACAGTGACACAAGAACTCAGCGGATCTCATATTCATTGAACCTAGAAG
CTGCTCGTTGACCCACGTTTTCCGACCGAAGGCACCGGCCCTCTGCTC
ATCTAGCGACAACTGTTGAAGGCTATGGTGTACAATCGTTCGCAACCA
TAAAAAACAGTTCGGACGGGCTTGAGCCTATCCCCCTCGAGGTAGAAA
GGCGACACTCTCTTCCAACTGACGAATGGATCCAAAA

>VAND
CCCTTCCTGTTTGTTTCTGCAGGTACGACCAGAAAGGCGTAGTGTACT
CCAATAAAGTCCAGAAAGGCAGGGCAACGTGTCTGCACGCCACGAACC
ACGCAGCTTCCTCAACCGCCTGCTGAGCACGCAACCCTGGACCCCCTT
CAGGCGATAGCGGAATGAGCAATTGTGCACCTGGGGTATAAACAGTTC
TTACAGGCAAGGGAACCGGACAAAGCAGGCAAGAGACTGAGGTGAGAA
AACAGTGACACAAGAACTCAGCGGATCTCATATTCATTGAACCTAGAAG
CTGCTCGTTGACCCACGTTTTCCGACCGAAGGCACCGGCCCTCTGCTC
ATCTAGCGACAACTGTTGAAGGCTATGGTGTACAATCGTTCGCAACCA
TAAAAAACAGTTCGGACGGGCTTGAGCCTATCCCCCTCGAGGTAGAAA
GGCGACACTCTCTTCCAACTGACGAATGGATCCAAAA

>TgCgCa1
CCCTTCCTGTTTGTTTCTGCAGGTACGACCAGAAAGGCGTAGTGTACT
CCAATAAAATCCAGAAAGGCAGGGCAACGTGTCTGCACGCCACGAACC
ACGCAGCTTCCTCAACCGCCTGCTGAGCACGCAACCCTGGACCCCCTT
CAGGCGATAGCGGAATGAGCAATTGTGCACCTGGGGTATAAACAGTTC
TTACAGGCAAGGGAACCGGACAAAGCAGGCAAGAGACTGAGGTGAGAA
AACAGTGACACAAGAACTCAGCGGATCTCATATTCATTGAACCTAGAAG
CTGCTCGTTGACCCACGTTTTCCGACCGAAGGCACCGGCCCTCTGCTC
ATCTAGCGACAACTGTTTAAGGCTATGGTGTACAATCGTTCGCAACCAT
AAAAAACAGTTCGGACGGGCTTGAGCCTATCCCCCTCGAGGTAGAAAG
GCGACACTCTCTTCCAACTGACGAATGGATCCAAAA

>ARI
CCCTTCCTGTTTGTTTCTGCAGGTACGACCAGAAAGGCGTAGTGTACT

CCAATAAAATCCAGAAAGGCAGGGCAACGTGTCTGCACGCCACGAACC
ACGCAGCTTCCTCAACCGCCTGCTGAGCACGCAACCCTGGACCCCCTT
CAGGCGATAGCGGAATGAGCAATTGTGCACCTGGGGCAGAAACAGTTC
TTACAGGCAAGGGAACCGGACAAAGCAGGCAAGAGACTGAGGTGAGAA
AACAGTGACACAAGAACTCAGCGGATCTCGTATTCATTGAACCTAGAAG
CTGCTCGTTGACCCACGTTTTCCGACCGAAGGCACCGGCCCTCTGCTC
ATCTAGCGACAACTGTTGAAGGCTATGGTGTACAATCGTTCGCAACCA
TAAAAAACAGTTCGGACGGGCTTGAGCCTATCCCCCTCGAGGTAGAAA
GGCGACACTCTCTTCCAACTGACGAATGGATCCAAAA

>TgCtPRC2
CCCTTCCTGTTTGTTTCTGCAGGTACGACCAGAAAGGCGTAGTGTACT
CCAATAAAATCCAGAAAGGCAGGGCAACGTGTCTGCACGCCACGAACC
ACGCAGCTTCCTCAACCGCCTGCTGAGCACGCAACCCTGGACCCCCTT
CAGGCGATAGCGGAATGAGCAATTGTGCACCTGGGGCAGAAACAGTTC
TTACAGGCAAGGGAACCGGACAAAGCAGGCAAGAGACTGAGGTGAGAA
AACAGTGACACAAGAACTCAGCGGATCTCGTATTCATTGAACCTAGAAG
CTGCTCGTTGACCCACGTTTTCCGACCGAAGGCACCGGCCCTCTGCTC
ATCTAGCGACAACTGTTGAAGGCTATGGTGTACAATCGTTCGCAACCA
TAAAAAACAGTTCGGACGGGCTTGAGCCTATCCCCCTCGAGGTAGAAA
GGCGACACTCTCTTCCAACTGACGAATGGATCCAAAA

>GAB2-2007-GAL-DOM2
CCCTTCCTGTTTGTTTCTGCAGGTACGACCAGAAAGGCGTAGTGTACT
CCAATAAAATCCAGAAAGGCAGGGCAACGTGTCTGCACGCCACGAACC
ACGCAGCTTCCTCAACCGCCTGCTGAGCACGCAACCCTGGACCCCCTT
CAGGCGATAGCGGAATGAGCAATTGTGCACCTGGGGTATAAACAGTTC
TTACAGGCAAGGGAACCGGACAAAGCAGGCAAGAGACTGAGGTGAGAA
AACAGTGACACAAGAACTCAGCGGATCTCATATTCATTGAACCTAGAAG
CTGCTCGTTGACCCACGTTTTCCGACCGAAGGCACCGGCCCTCTGCTC
ATCTAGCGACAACTGTTGAAGGCTATGGTGTACAATCGTTCGCAACCA
TAAAAAACAGTTCGGACGGGCTTGAGCCTATCCCCCTCGAGGTAGAAA
GGCGACACTCTCTTCCAACTGACGAATGGATCCAAAA

>TgCtCo5
CCCTTCCTGTTTGTTTCTGCAGGTACGACCAGAAAGGCGTAGTGTACT

CCAATAAAATCCAGAAAGGCAGGGCAACGTGTCTGCACGCCACGAACC
ACGCAGCTTCCTCAACCGCCTGCTGAGCACGCAACCCTGGACCCCCTT
CAGGCGATAGCGGAATGAGCAATTGTGCACCTGGGGTATAAACAGTTC
TTACAGGCAAGGGAACCGGACAAAGCAGGCAAGAGACTGAGGTGAGAA
AACAGTGACACAAGAACTCAGCGGATCTCATATTCATTGAACCTAGAAG
CTGCTCGTTGACCCACGTTTTCCGACCGAAGGCACCGGCCCTCTGCTC
ATCTAGCGACAACTGTTGAAGGCTATGGTGTACAATCGTTCGCAACCA
TAAAAAACAGTTCGGACGGGCTTGAGCCTATCCCCCTCGAGGTAGAAA
GGCGACACTCTCTTCCAACTGACGAATGGATCCAAAA

>TgCatJpOk1
AAAGATTGATGAAACAGAAAGAATGATGTGGCGTGAGCCCGCAAAAGT
GACTATAACACGAACCGTAAAGACTCGGCAAAAAGTGAAGGCAAACGA
GGCACACCACAACCAGCGAAGCTGCAGGAATAATACAGTCAGTTCCCT
CGATCGCAGCACTCGAATACAGCTGCCGAAAAGCATACTGCCCTTCCT
GTTTGTTTCTGCAGGTACGACCAGAAAGGCGTAGTGTACTCCAATAAA
ATCCAGAAAGGCAGGGCAACGTGTCTGCACGCCACGAACCACGCAGCT
TCCTCAACCGCCTGCTGAGCACGCAACCCTGGACCCCCTTCAGGCGAT
AGCGGAATGAGCAATTGTGCACCTGGGGCAGAAACAGTTCTTACAGGC
AAGGGAACCGGACAAAGCAGGCAAGAGACTGAGGTGAGAAAACAGTGA
CACAAGAACTCAGCGGATCTCGTATTCATTGAACCTAGAAGCTGCTCG
TTGACCCACGTTTTCCGACCGAAGGCACCGGCCCTCTGCTCATCTAGC
GACAACTGTTGAAGGCTATGGTGTACAATCGTTCGCAACCATAAAAAAC
AGTTCGGACGGGCTTGAGCCTATCCCCCTCGAGGTAGAAAGGCGACAC
TCTCTTCCAACTGACGAATGGATCCAAAACGCA

>TgCatJpOk2
TGAGCACAAGAAGAGCAAACGAAACTAACCGGAAACAGGAAACAGTTG
ACTCTCCCCTTCCGCGCTAGGCACGCACCCCGTGAGTTAACAAAGATG
GAAAGATTGATGAAACAGAAAGAATGATGTGGCGTGAGCCCGCAAAAG
TGACTATAACACGAACCGTAAAGACTCGGCAAAAAGTGAAGGCAAACG
AGGCACACCACAACCAGCGAAGCTGCAGGAATAATACAGTCAGTTCCC
TCGATCGCAGCACTCGAATACAGCTGCCGAAAAGCATACTGCCCTTCC
TGTTTGTTTCTGCAGGTACGACCAGAAAGGCGTAGTGTACTCCAATAA
AATCCAGAAAGGCAGGGCAACGTGTCTGCACGCCACGAACCACGCAGC
TTCCTCAACCGCCTGCTGAGCACGCAACCCTGGACCCCCTTCAGGCGA

TAGCGGAATGAGCAATTGTGCACCTGGGGCAGAAACAGTTCTTACAGG
CAAGGGAACCGGACAAAGCAGGCAAGAGACTGAGGTGAGAAAACAGTG
ACACAAGAACTCAGCGGATCTCGTATTCATTGAACCTAGAAGCTGCTC
GTTGACCCACGTTTTCCGACCGAAGGCACCGGCCCTCTGCTCATCTAG
CGACAACTGTTGAAGGCTATGGTGTACAATCGTTCGCAACCATAAAAA
ACAGTTCGGACGGGCTTGAGCCTATCCCCCTCGAGGTAGAAAGGCGAC
ACTCTCTTCCAACTGACGAATGGATCCAAAACGCAGCAAGCATG

>TgCatJpOk3
CAGAAACACGCAGAGAATATGTCACCGATTACACACCCGGAAATGCGAA
AAAATGAGCAGAACACGATGAGCACAAGAAGAGCAAACGAAACTAACCG
GAAACAGGAAACAGTTGACTCTCCCCTTCCGCGCTAGGCACGCACCCC
GTGAGTTAACAAAGATGGAAAGATTGATGAAACAGAAAGAATGATGTG
GCGTGAGCCCGCAAAAGTGACTATAACACGAACCGTAAAGACTCGGCA
AAAAGTGAAGGCAAACGAGGCACACCACAACCAGCGAAGCTGCAGGAA
TAATACAGTCAGTTCCCTCGATCGCAGCACTCGAATACAGCTGCCGAAA
AGCATACTGCCCTTCCTGTTTGTTTCTGCAGGTACGACCAGAAAGGCG
TAGTGTACTCCAATAAAATCCAGAAAGGCAGGGCAACGTGTCTGCACG
CCACGAACCACGCAGCTTCCTCAACCGCCTGCTGAGCACGCAACCCTG
GACCCCCTTCAGGCGATAGCGGAATGAGCAATTGTGCACCTGGGGCA
GAAACAGTTCTTACAGGCAAGGGAACCGGACAAAGCAGGCAAGAGACT
GAGGTGAGAAAACAGTGACACAAGAACTCAGCGGATCTCGTATTCATT
GAACCTAGAAGCTGCTCGTTGACCCACGTTTTCCGACCGAAGGCACCG
GCCCTCTGCTCATCTAGCGACAACTGTTGAAGGCTATGGTGTACAATC
GTTCGCAACCATAAAAAACAGTTCGGACGGGCTTGAGCCTATCCCCCT
CGAGGTAGAAAGGCGACACTCTCTTCCAACTGACGAATGGATCCAAAA
CGCAGCAAGCATGGGAACAAAGATTACGGACACTCAGCGTTCGAGTCT
TACCGCCGCCCGACGTTGGGCCATGATTTCTGCCTCTGTTGAGTCGC
TCGTCTCC

>TgCatJpOk4
TGAGCAGAACACGATGAGCACAAGAAGAGCAAACGAAACTAACCGGAA
ACAGGAAACAGTTGACTCTCCCCTTCCGCGCTAGGCACGCACCCCGTG
AGTTAACAAAGATGGAAAGATTGATGAAACAGAAAGAATGATGTGGCG
TGAGCCCGCAAAAGTGACTATAACACGAACCGTAAAGACTCGGCAAAA
AGTGAAGGCAAACGAGGCACACCACAACCAGCGAAGCTGCAGGAATAA

TACAGTCAGTTCCCTCGATCGCAGCACTCGAATACAGCTGCCGAAAAG
CATACTGCCCTTCCTGTTTGTTTCTGCAGGTACGACCAGAAAGGCGTA
GTGTACTCCAATAAAATCCAGAAAGGCAGGGCAACGTGTCTGCACGCC
ACGAACCACGCAGCTTCCTCAACCGCCTGCTGAGCACGCAACCCTGGA
CCCCCTTCAGGCGATAGCGGAATGAGCAATTGTGCACCTGGGGCAGA
AACAGTTCTTACAGGCAAGGGAACCGGACAAAGCAGGCAAGAGACTGA
GGTGAGAAAACAGTGACACAAGAACTCAGCGGATCTCGTATTCATTGA
ACCTAGAAGCTGCTCGTTGACCCACGTTTTCCGACCGAAGGCACCGGC
CCTCTGCTCATCTAGCGACAACTGTTGAAGGCTATGGTGTACAATCGT
TCGCAACCATAAAAAACAGTTCGGACGGGCTTGAGCCTATCCCCCTCG
AGGTAGAAAGGCGACACTCTCTTCCAACTGACGAATGGATCCAAAACG
C

>TgCatJpTy1/k-3
GACAGAAACACGCAGAGAATATGTCACCGATTACACACCCGGAAATGC
GAAAAAATGAGCAGAACACGATGAGCACAAGAAGAGCAAACGAAACTAA
CCGGAAACAGGAAACAGTTGACTCTCCCCTTCCGCGCTAGGCACGCAC
CCCGTGAGTTAACAAAGATGGAAAGATTGATGAAACAGAAAGAATGAT
GTGGCGTGAGCCCGCAAAAGTGACTATAACACGAACCGTAAAGACTCG
GCAAAAAGTGAAGGCAAACGAGGCACACCACAACCAGCGAAGCTGCAG
GAATAATACAGTCAGTTCCCTCGATCGCAGCACTCGAATACAGCTGCC
GAAAAGCATACTGCCCTTCCTGTTTGTTTCTGCAGGTACGACCAGAAA
GGCGTAGTGTACTCCAATAAAATCCAGAAAGGCAGGGCAACGTGTCTG
CACGCCACGAACCACGCAGCTTCCTCAACCGCCTGCTGAGCACGCAAC
CCTGGACCCCCTTCAGGCGATAGCGGAATGAGCAATTGTGCACCTGG
GGCAGAAACAGTTCTTACAGGCAAGGGAACCGGACAAAGCAGGCAAGA
GACTGAGGTGAGAAAACAGTGACACAAGAACTCAGCGGATCTCGTATT
CATTGAACCTAGAAGCTGCTCGTTGACCCACGTTTTCCGACCGAAGGC
ACCGGCCCTCTGCTCATCTAGCGACAACTGTTGAAGGCTATGGTGTAC
AATCGTTCGCAACCATAAAAAACAGTTCGGACGGGCTTGAGCCTATCC
CCCCTCGAGGTAGAAAGGCGACACTCTCTTCCAACTGACGAATGGATC
CAAAACGCAGCAAGCATGGGAACAAAGATTA

>TgCatJpGi1/TaJ
TACACACCCGGAAATGCGAAAAAATGAGCAGAACACGATGAGCACAAG
AAGAGCAAACGAAACTAACCGGAAACAGGAAACAGTTGACTCTCCCCT

TNTGCGCCAGGCACGCACCCCGTGAGTTAACAAAGATGGAAAGATTGA
TGAAACAGAAAGAATGATGTGGCGTGAGCCCGCAAAAGTGACTATAAC
ACGAACCGTAAAGACTCGGCAAAAAGTGAAGGCAAACGAGGCACACCA
CAACCAGCGAAGCTGTAGGAATAATACAGTCAGTTCCCTCGATCGCGG
CACTCGAATACAGCTGCCGAAAAGCATACTGCCCTTCCTGTTTGTTTC
TGCAGGTACGACCAGAAAGGCGTAGTGTACTCCAATAAAGTCCAGAAA
GGCAGGGCAACGTGTCTGCACGCCACGAACCACGCAGCTTCCTCAACC
GCCTGCTGAGCACGCAACCCTGGACCCCCTTCAGGCGATAGCGGAAT
GAGCAATTGTGCACCTGGGGTATAAACAGTTCTTACAGGCAAGGGAAC
CGGACAAAGCAGGCAAGAGACTGAGGTGAGAAAACAGTGACACAAGAA
CTCAGCGGATCTCATATTCATTGAACCTAGAAGCTGCTCGTTGACCCA
CGTTTTCCGACCGAAGGCACCGGCCCTCTGCTCATCTAGCGACAACTG
TTGAAGGCTATGGTGTACAATCGTTCGCAACCATAAAAAACAGTTCGG
ACGGGCTTGAGCCTATCCCCCTCGAGGTAGAAAGGCGACACTCTCTTC
CAACTGACGAATGGATCCAAAACGCAGCAAGCATGGGACCAAAAGATT
A

>TgGoatJpOk1
TGACAGAAACACGCAGAGAATATGTCACCGATTACACACCCGGAAATGC
GAAAAAATGAGCAGAACACGATGAGCACAAGAAGAGCAAACGAAACTAA
CCGGAAACAGGAAACAGTTGACTCTCCCCTTCCGCGCTAGGCACGCAC
CCCGTGAGTTAACAAAGATGGAAAGATTGATGAAACAGAAAGAATGAT
GTGGCGTGAGCCCGCAAAAGTGACTATAACACGAACCGTAAAGACTCG
GCAAAAAGTGAAGGCAAACGAGGCACACCACAACCAGCGAAGCTGCAG
GAATAATACAGTCAGTTCCCTCGATCGCAGCACTCGAATACAGCTGCC
GAAAAGCATACTGCcCTTCCTGTTTGTTTCTGCAGGTACGACCAGAAA
GGCGTAGTGtactCcaatAAAATCCAGAAAGGCAGGGCAACGTGTCTGCAC
GCCACgAACCACGCAGCTTCCTCAACCGCCTGCTGAGCACGCAACCCT
GGACCCCCTTCAGGCGATAGCGGAATGAGCAATTGTGCACCTGGGGC
AGAAACAGTTCTTACAGGCAAGGGAACCGGACAAAGCAGGCAAGAGAC
TGAGGTGAGAAAACAGTGACACAAGAACTCAGCGGATCTCGTATTCAT
TGAACCTAGAAGCTGCTCGTTGACCCACGTTTTCCGACCGAAGGCACC
GGCCCTCTGCTCATCTAGCGACAACTGTTGAAGGCTATGGTGTAcaATC
GTTCGCAACCATAAAAAACAGTTCGGAcGGGCTTGAGCCTATCCCCCT
CGAGGTAgAAAGGCGACACTCTCTTCcaaCTGACGAATGGATCCAAACG

>TgGoatJpOk2
AGAAACACGCAGAGAATATGTCACCGATTACACACCCGGAAATGCGAAA
AAATGAGCAGAACACGATGAGCACAAGAAGAGCAAACGAAACTAACCG
GAAACAGGAAACAGTTGACTCTCCCCTTCCGCGCTAGGCACGCACCCC
GTGAGTTAACAAAGATGGAAAGATTGATGAAACAGAAAGAATGATGTG
GCGTGAGCCCGCAAAAGTGACTATAACACGAACCGTAAAGACTCGGCA
AAAAGTGAAGGCAAACGAGGCACACCACAACCAGCGAAGCTGCAGGAA
TAATACAGTCAGTTCCCTCGATCGCAGCACTcgAAcgCAGCTGCCGAAAA
GCATACTGCCCTTCCTGTTTGTTTCTGCAGGTACGACCAGAAAGGCGT
AGTGTACTCCAATAAAATCCAGAAAGGCAGGGCAACGTGTCTGCACGC
CACGAACCACGCAGCTTCCTCAACCGCCTGCTGAGCACGCAACCCTGG
ACCCCCTTCAGGCGATAGCGGAATGAGCAATTGTGCACCTGGGGCAGA
AACAGTTCTTACAGGCAAGGGAACCGGACAAAGCAGGCAAGAGACTGA
GGTGAGAAAACAGTGACACAAGAACTCAGCGGATCTCGTATTCATTGA
ACCTAGAAGCTGCTCGTTGACCCACGTTTTCCGACCGAAGGCACCGGC
CCTCTGCTCATCTAGCGACAACTGTTGAAGGCTATGGTGTACAATCGT
TCGCAACCATAAAAAACAGTTCGGACGGGCTTGAGCCTATCCCCCTCG
AGGTAGAAAGGCGACACTCTCTTCCAACTGACGAATGGATCCAAAACG
CAGCAAGCATG

>TgGoatJpOk3
GAAACTAACCGGAAACAGGAAACAGTTGACTCTCCCCTTCCGCGCTAG
GCACGCACCCCGTGAGTTAACAAAGATGGAAAGATTGATGAAACAGAA
AGAATGATGTGGCGTGAGCCCGCAAAAGTGACTATAACACGAACCGTA
AAGACTCGGCAAAAAGTGAAGGCAAACGAGGCACACCACAACCAGCGA
AGCTGCAGGAATAATACAGTCAGTTCCCTCGATCGCAGCACTCGAATAC
AGCTGCCGAAAAGCATACTGCCCTTCCTGTTTGTTTCTGCAGGTACGA
CCAGAAAGGCGTAGTGTACTCCAATAAAATCCAGAAAGGCAGGGCAAC
GTGTCTGCACGCCACGAACCACGCAGCTTCCTCAACCGCCTGCTGAGC
ACGCAACCCTGGACCCCCTTCAGGCGATAGCGGAATGAGCAATTGTGC
ACCTGGGGCAGAAACAGTTCTTACAGGCAAGGGAACCGGACAAAGCAG
GCAAGAGACTGAGGTGAGAAAACAGTGACACAAGAACTCAGCGGATCT
CGTATTCATTGAACCTAGAAGCTGCTCGTTGACCCACGTTTTCCGACC
GAAGGCACCGGCCCTCTGCTCATCTAGCGACAACTGTTGAAGGCTATG
GTGTACAATCGTTCGCAACCATAAAAAACAGTTCGGACGGGCTTGAGC
CTATCCCCCTCGAGGTAGAAAGGCGACACTCTCTTCCAACTGACGAAT

GGATCCAAAACGCAGCAAGCA

>TgGoatJpOk4
AATGTTCCACCCTGAACCTGGCATGGAGTCCGGGAACGGGGAAGGAA
GGACGACGAAAACCGAGCCGGGCAAGTCAGGGTGGTAAGAGATTCTAT
TTTTAACATTTCAACGAGCTGTTGAGCTGTGGTATCTAAAGCGCTAGG
GGATCTTAAGATTAATACAACAATTGANTAATGGTCTCTGGGAACGAG
TAGGGATTGGNTGGAATTAGGTGCATTAATGACAGAAACAGGCAGAGA
ATATGTCACCGATTACACACCCGGAAATGCGAAAAAATGAGCAGAACAC
GATGAGCACAAGAAGAGCAAACGAAACTAACCGGAAACAGGAAACAGT
TGACTCTCCCCTTCCGCGCTAGGCACGCACCCCGTGAGTTAACAAAGA
TGGAAAGATTGATGAAACAGAAAGAATGATGTGGCGTGAGCCCGCAAA
AGTGACTATAACACGAACCGTAAAGACTCGGCAAAAAGTGAAGGCAAAC
GAGGCACACCACAACCAGCGAAGCTGCAGGAATAATACAGTCAGTTCC
CTCGATCGCAGCACTCGAATACAGCTGCCGAAAAGCATACTGCCCTTC
CTGTTTGTTTCTGCAGGTACGACCAGAAAGGCGTAGTGTACTCCAATA
AAATCCAGAAAGGCAGGGCAACGTGTCTGCACGCCACGAACCACGCAG
CTTCCTCAACCGCCTGCTGAGCACGCAACCCTGGACCCCCTTCAGGCG
ATAGCGGAATGAGCAATTGTGCACCTGGGGCAGAAACAGTTCTTACAG
GCAAGGGAACCGGACAAAGCAGGCAAGAGACTGAGGTGAGAAAACAGT
GACACAAGAACTCAGCGGATCTCGTATTCATTGAACCTAGAAGCTGCT
CGTTGACCCACGTTTTCCGACCGAAGGCACCGGCCCTCTGCTCATCTA
GCGACAACTGTTGAAGGCTATGGTGTACAATCGTTCGCAACCATAAAA
AACAGTTCGGACGGGCTTGAGCCTATCCCCCTCGAGGTAGAAAGGCGA
CACTCTCTTCCAACTGACGAATGGATCCAAAACGCAG

>TgGoatJpOk5
TGACAGAAACACGCAGAGAATATGTCACCGATTACACACCCGGAAATGC
GAAAAAATGAGCAGAACACGATGAGCACAAGAAGAGCAAACGAAACTAA
CCGGAAACAGGAAACAGTTGACTCTCCCCTTCCGCGCTAGGCACGCAC
CCCGTGAGTTAACAAAGATGGAAAGATTGATGAAACAGAAAGAATGAT
GTGGCGTGAGCCCGCAAAAGTGACTATAACACGAACCGTAAAGACTCG
GCAAAAAGTGAAGGCAAACGAGGCACACCACAACCAGCGAAGCTGCAG
GAATAATACAGTCAGTTCCCTCGATCGCAGCACTCGAATACAGCTGCC
GAAAAGCATACTGCCCTTCCTGTTTGTTTCTGCAGGTACGACCAGAAA
GGCGTAGTGTACTCCAATAAAATCCAGAAAGGCAGGGCAACGTGTCTG

CACGCCACGAACCACGCAGCTTCCTCAACCGCCTGCTGAGCACGCAAC
CCTGGACCCCCTTCAGGCGATAGCGGAATGAGCAATTGTGCACCTGG
GGCAGAAACAGTTCTTACAGGCAAGGGAACCGGACAAAGCAGGCAAGA
GACTGAGGTGAGAAAACAGTGACACAAGAACTCAGCGGATCTCGTATT
CATTGAACCTAGAAGCTGCTCGTTGACCCACGTTTTCCGACCGAAGGC
ACCGGCCCTCTGCTCATCTAGCGACAACTGTTGAAGGCTATGGTGTAC
AATCGTTCGCAACCATAAAAAACAGTTCGGACGGGCTTGAGCCTATCC
CCCTCGAGGTAGAAAGGCGACACTCTCTTCCAACTGACGAATGGATCC
AAAACGCAGCAAGCATGGGAAC

>TgGoatJpOk6
CGGAAATGCGAAAAAATGAGCAGAACACGATGAGCACAAGAAGAGCAA
ACGAAACTAACCGGAAACAGGAAACAGTTGACTCTCCCCTTCCGCGCT
AGGCACGCACCCCGTGAGTTAACAAAGATGGAAAGATTGATGAAACAG
AAAGAATGATGTGGCGTGAGCCCGCAAAAGTGACTATAACACGAACCG
TAAAGACTCGGCAAAAAGTGAAGGCAAACGAGGCACACCACAACCAGC
GAAGCTGCAGGAATAATACAGTCAGTTCCCTCGATCGCAGCACTCGAA
TACAGCTGCCGAAAAGCATACTGCCCTTCCTGTTTGTTTCTGCAGGTA
CGACCAGAAAGGCGTAGTGTACTCCAATAAAATCCAGAAAGGCAGGGC
AACGTGTCTGCACGCCACGAACCACGCAGCTTCCTCAACCGCCTGCTG
AGCACGCAACCCTGGACCCCCTTCAGGCGATAGCGGAATGAGCAATTG
TGCACCTGGGGCAGAAACAGTTCTTACAGGCAAGGGAACCGGACAAAG
CAGGCAAGAGACTGAGGTGAGAAAACAGTGACACAAGAACTCAGCGGA
TCTCGTATTCATTGAACCTAGAAGCTGCTCGTTGACCCACGTTTTCCG
ACCGAAGGCACCGGCCCTCTGCTCATCTAGCGACAACTGTTGAAGGCT
ATGGTGTACAATCGTTCGCAACCATAAAAAACAGTTCGGACGGGCTTG
AGCCTATCCCCCTCGAGGTAGAAAGGCGACACTCTCTTCCAACTGACG
AATGGATCCAAAACGCAGCAAGCATGGGNNCAAAAGATTAA

>TgGoatJpOk7
AGAGAATATGTCACCGATTACACACCCGGAAATGCGAAAAAATGAGCAG
AACACGATGAGCACAAGAAGAGCAAAcGAAACTAACCGGAAACAGGAAA
CAGTTGACTCTCCCCTTCCGCGCTAGGCACGCACCCCGTGAGTTAACA
AAGATGGAAAGATTGATGAAACAGAAAGAATGATGTGGCGTGAGCCCG
CAAAAGTGACTATAACACGAACCGTAAAGACTCGGCAAAAAGTGAAGG
CAAACGAGGCACACCACAACCAGCGAAGCTGCAGGAATAATACAGTCA

GTTCCCTCGATCGCAGCACTCGAATACAGCTGCCGAAAAGCATACTGC
CCTTCCTGTTTGTTTCTGCAGGTACGACCAGAAAGGCGTAgTGTACTC
CAATAAAATCCAGAAAGGCAGGGCAACGTGTCTGCACGCCACGAACCA
CGCAGCTTCCTCAACCGCCTGCTGAGCACGCAACCCTGGACCCCCTTC
AGGCGATAGCGGAATGAGCAATTGTGCACCTGGGGCAGAAACAGTTCT
TACAGGCAAGGGAACCGGACAAAGCAGGCAAGAGACTGAGGTGAGAAA
ACAGTGACACAAGAACTCAGCGGATCTCGTATTCATTGAACCTAGAAGC
TGCTCGTTGACCCACGTTTTCCGACCGAAGGCACCGGCCCTCTGCTCA
TCTAGCGACAACTGTTGAAGGCTATGGTGTACAATCGTTCGCAACCAT
AAAAAACAGTTCGGACGGGCTTGAGCCTATCCCCCTCGAGGTAGAAAG
GCGACACTCTCTTCCAACTGACGAATGGATCCAAAACGCAGCAAGCAT
G

>TgGoatJpOk8
CAGAGAATATGTCACCGATTACACACCCGGAAATGNGAAAAAATGAGC
AGAACACGATGAGCACAAGAAGAGCAAACGAAACTAACCGGAAACAGG
AAACAGTTGACTCTCCCCTTCCGCGCTAGGCACGCACCCCGTGAGTTA
ACAAAGATGGAAAGATTGATGAAACAGAAAGAATGATGTGGCGTGAGC
CCGCAAAAGTGACTATAACACGAACCGTAAAGACTCGGCAAAAAGTGAA
GGCAAACGAGGCACACCACAACCAGCGAAGCTGCAGGAATAATACAGT
CAGTTCCCTCGATCGCAGCACTCGAATACAGCTGCCGAAAAGCATACT
GCCCTTCCTGTTTGTTTCTGCAGGTACGACCAGAAAGGCGTAgTGTAC
TCCAATAAAATCCAgAAAGGCAGGGCAACGTGTCTGCACGCCACGAAC
CACGCAGCTTCCTCAACCGCCTGCTGAGCACGCAACCCTGGACCCCCT
TCAGGCGATAGCGGAATGAGCAATTGTGCACCTGGGGCAGAAACAGTT
CTTACAGGCAAGGGAACCGGACAAAGCAGGCAAGAGACTGAGGTGAGA
AAACAGTGACACAAGAACTCAGCGGATCTCGTATTCATTGAACCTAGAA
GCTGCTCGTTGACCCACGTTTTCCGACCGAAGGCACCGGCCCTCTGC
TCATCTAGCGACAACTGTTGAAGGCTATGGTGTACAATCGTTCGCAAC
CATAAAAAACAGTTCGGACGGGCTTGAGCCTATCCCCCTCGAGGTAGA
AAGGCGACACTCTCTTCCAACTGACGAATGGATCCAAAACGCAGCAAG
CATGGG

>TgGoatJpOk9
AGAAACACGCAGAGAATATGTCACCGATTACACACCCGGAAATGCGAAA
AAATGAGCAGAACACGATGAGCACAAGAAGAGCAAACGAAACTAACCG

GAAACAGGAAACAGTTGACTCTCCCCTTCCGCGCTAGGCACGCACCCC
GTGAGTTAACAAAGATGGAAAGATTGATGAAACAGAAAGAATGATGTG
GCGTGAGCCCGCAAAAGTGACTATAACACGAACCGTAAAGACTCGGCA
AAAAGTGAAGGCAAACGAGGCACACCACAACCAGCGAAGCTGCAGGAA
TAATACAGTCAGTTCCCTCGATCGCAGCACTCGAATACAGCTGCCGAAA
AGCATACTGCCCTTCCTGTTTGTTTCTGCAGGTACGACCAGAAAGGCG
TAGTGTACTCCAATAAAATCCAGAAAGGCAGGGCAACGTGTCTGCACG
CCACGAACCACGCAGCTTCCTCAACCGCCTGCTGAGCACGCAACCCTG
GACCCCCTTCAGGCGATAGCGGAATGAGCAATTGTGCACCTGGGGCA
GAAACAGTTCTTACAGGCAAGGGAACCGGACAAAGCAGGCAAGAGACT
GAGGTGAGAAAACAGTGACACAAGAACTCAGCGGATCTCGTATTCATT
GAACCTAGAAGCTGCTCGTTGACCCACGTTTTCCGACCGAAGGCACCG
GCCCTCTGCTCATCTAGCGACAACTGTTGAAGGCTATGGTGTACAATC
GTTCGCAACCATAAAAAACAGTTCGGACGGGCTTGAGCCTATCCCCCT
CGAGGTAGAAAGGCGACACTCTCTTCCAACTGACGAATGGATCCAAAA
CGCAGCAAGCATG

>TgGoatJpOk10
TACTGCCCTTCCTGTTTGTTTCTGCAGGTACGACCAGAAAGGCGTAGT
GTACTCCAATAAAATCCAGAAAGGCAGGGCAACGTGTCTGCACGCCAC
GAACCACGCAGCTTCCTCAACCGCCTGCTGAGCACGCAACCCTGGACC
CCCTTCAGGCGATAGCGGAATGAGCAATTGTGCACCTGGGGCAGAAAC
AGTTCTTACAGGCAAGGGAACCGGACAAAGCAGGCAAGAGACTGAGGT
GAGAAAACAGTGACACAAGAACTCAGCGGATCTCGTATTCATTGAACC
TAGAAGCTGCTCGTTGACCCACGTTTTCCGACCGAAGGCACCGGCCCT
CTGCTCATCTAGCGACAACTGTTGAAGGCTATGGTGTACAATCGTTCG
CAACCATAAAAAACAGTTCGGACGGGCTTGAGCCTATCCCCCTCGAGG
TAGAAAGGCGACACTCTCTTCCAACTGACGAATGGATCCAAAACGCAG
CAAG

>TgGoatJpOk11
CACGCAGAGAATATGTCACCGATTACACACCCGGAAATGCGAAAAAATG
AGCAGAACACGATGAGCACAAGAAGAGCAAACGAAACTAACCGGAAACA
GGAAACAGTTGACTCTCCCCTTCTGCGCCAGGCACGCACCCCGTGAGT
TAACAAAGATGGAAAGATTGATGAAACAGAAAGAATGATGTGGCGTGA
GCCCGCAAAAGTGACTATAACACGAACCGTAAAGACTCGGCAAAAAGT

GAAGGCAAACGAGGCACACCACAACCAGCGAAGCTGTAGGAATAATAC
AGTCAGTTCCCTCGATCGCGGCACTCGAATACAGCTGCCGAAAAGCAT
ACTGCCCTTCCTGTTTGTTTCTGCAGGTACGACCAGAAAGGCGTAGTG
TACtCcaataAAGTCCAGAAAGGCAGGGCAACGTGTCTGCACGCCACGAA
CCACGCAGCTTCCTCAACCGCCTGCTGAGCACGCAACCCTGGACCCCC
TTCAGGCGATAGCGGAATGAGCAATTGTGCACCTGGGGTATAAACAGT
TCTTACAGGCAAGGGAACCGGACAAAGCAGGCAAGAGACTGAGGTGAG
AAAACAGTGACACAAGAACTCAGCGGATCTCATATTCATTGAACCTAGA
AGCTGCTCGTTGACCCACGTTTTCCGACCGAAGGCACCGGCCCTCTG
CTCATCTAGCGACAACTGTTGAAGGCTATGGTGTACAATCGTTCGCAA
CCATAAAAAACAGTTCGGACGGGCTTGAGCCTATCCCCCTCGAGGTAG
AAAGGCGACACTCTCTTCCAACTGACGAATGGATCCAAAACGCAGCAA
GCATGGGAACAAAGATTA

Uracil phosphoribosyl transferase intron 1 (UPRT1)

>GT1
TGCGGCCCCCTTTCCTCCGCTTGCTGTTTAAATGCTCGTCCTCGTTTT
CCTTCCTGCCGCATCCCCGTATATTTTAAGGAGAGGGAAACAGGCGTG
AGTTGGACGGAATGAAAGTTCTCGGCCTGTACGCCGGTTGTCGCGGT
CGTTTGCAGATTGCTTTTTTCTTCGAATCGGTGCTGTAACCCTCGCGA
AGAACGACGCTGCAAACGACTTCTCGAACTCTCAGTCGTGTACTTTAC
GTGCTTCCTTTCAGGGACCTCCCCCTGCGTTACTCATTTGTATTCACA
GCTACGAAGTGTCTTGCAAGGTGGATTCCTGCCAGGCTCCATGTCTCA
CTCGGTGCGTTTTCGGAAAAGTTCATTGTGAACGTTCCCCTTGCGTG
TCATGACTTTATCAG

>ME49
CCCGATATTCGACAAACGACCAGGAAGAAAGCATTCTCCAGGACATCAT
CACGAGGTAATCCTTCAACCGAAGTTTGCTTTCCGTGACTCTGCCTGT
TGGTTATACTGCGTGGCCTTCCCGTCCTGCGGCCCCCTTTCCTCCGC
TTGCTGTTTAAATGCTCGTCCTCGTTTTCCTTCCTGCCGCATCCCCGT
ATATTTTAAGGAGAGGGAAACAGGCGTGAGTTGGACGGCATGAAAGTT
CTCGGCCTGTATGCCGGTTGTGGCGGTCGTTTGCAGATTGCTTTTTT
CTTCGAATCGGTGCTGTAACCCTCGCGAAGAACGACGCTGCAAACGAC
TTCTCGAACTCTCAGTCGTGTACTTTACGTGCTTCCTTTCAGGGACCT
CCCCCCGCGTTACTCATTTGTATTCACAGCTACGAAGTGTCTTGCAAG
GTGGATTTCTGCCAGGCTCCATGTCTCACTCGTTGCGTTTTCGGAAAA
GTTCATTGTGAACGTTCCCCTTGCGTGTCATGACTTTATCAGGTTTCC
CAATGTGGTGCTCATGAAGCAGACGGCTC

>VEG
TGCGGCCCCCTTTCCTCCGCTTGCTGTTTAAATGCTCGTCCTCGTTTT
CCTTCCTGCCGCATCCCCGTATATTTTAAGGAGAGGGAAACAGGCGTG
AGTTGGACGGCATGAAAGTTCTCGGCCTGTATGCCGGTTGTGGCGGT
CGTTTGCAGATTGCTTTTTTCTTCGAATCGGTGCTGTAACCCTCGCGA
AGAACGACGCTGCAAACGACTTCTCGAACTCTCAGTCGTGTACTTTAC
GTGCTTCCTTTCAGGGACCTCCCCCCGCGTTACTCATTTGTATTCACA
GCTACGAAGTGTCTTGCAAGGTGGATTTCTGCCAGGCTCCATGTCTCA
CTCGTTGCGTTTTCGGAAAAGTTCATTGTGAACGTTCCCCTTGCGTGT

CATGACTTTATCAG

>MAS
TGCGGCCCCCTTTCCTCCGCTTGCTGTTTAAATGCTCGTCCTCGTTTT
CCTTCCTGCCGCATCCCCGTATATTTTAAGGAGAGGGAAACAGGCGTG
AGTTGGACGGAATGAAAGTTCTCGGCCTGTACGCCGGTTGTCGCGGT
CGTTTGCAGATTGCTTTTTTCTTCGAATCGGTGCTGTAACCCTCGAAG
AAGAACGACGCTGCAAACGACTTGTCGAACTCTCAGTCGTGTACTTTA
CGTGCTTCCTTTCAGGGACCTCCCTCCGCGTTACTCATTTGTATTCAC
AGCTACGAAGTGTCTTGCAAGGTGGATTCGTGCCAGGCTCCATGTCTC
ACTCGGTGCGTTTTCGGAAAAGTTCATTGTGAACGTTCCCCTTGCGT
GTCATGACTTTATCAG

>RUB
TGCCGCTCCCTTTCCTCCGCTTGCTGTTTAAATGCTCGTCCTCGTTTT
CCTTCCTGCCGCATCCCCGTATATTTTAAGGAGAGGGAAACAGGCGTG
AGTTGGACGGAATGAAAGTTCTCGGCCTGTACGCCGGTTGTCGCGGT
CGTCTGCAGATTGCTTTTTTCTTCGAATCGGTGCTGTAACCCTCGCGA
AGAACGACGCTGCAAACGACTTCTCGAACTCTCAGTCGTGTACTTTAC
GTGCTTCCTTTCAGGGACCTCCCCCCGCGTTACTCATTTGTATTCACA
GCTACGAAGTGTCTTGCAAGGTGGATTCGTGACAGGCTCCATGTCTCA
CTCGGTGCATTTTCGGAAAAGTTCATTGTGAACGTTGCCCTTGCGTGT
CATGACTTTATCAG

>FOU
TGCGGCCCCCTTTCCTCCGCTTGCTGTTTAAATGCTCGTCCTCGTTTT
CCTTCCTGCCGCATCCCCGTATATTTTAAGGAGAGGGAAACAGGCGTG
AGTTGGACGGAATGAAAGTTCTCGGCCTGTACGCCGGTTGTCGCGGT
CGTTTGCAGATTGCTTTTTTCTTCGAATCGGTGCTGTAACCCTCGCGA
AGAACGACGCTGCAAACGACTTCTCGAACTCTCAGTCGTGTACTTTAC
GTGCTTCCTTTCAGGGACCTCCCCCCGCGTTACTCATTTGTATTCACA
GCTACGAAGTGTCTTGCAAGGTGGATTCGTGACAGGCTCCATGTCTCA
CTCGGTGCATTTTCGGAAAAGTTCATTGTGAACGTTGCCCTTGCGTGT
CATGACTTTATCAG

>CAST

TGCGGCCCCCTTTCCTCCGCTTGCTGTTTAAATGCTCGTCCTCGTTTT
CCTTCCTGCCGCATCCCCGTATATTTTAAGGAGAGGGAAACAGGCGTG
AGTTGGACGGCATGACGGTTCTCGGCCTGTATGCCGGTTGTCGCGGT
CGTTTGCAGATTGCTTTTTTCTTCGAATCGGTGCTGTAACCCTCGCGA
AGAACGACGCTGCAAACGACTTCTCGAACTCTCAGTCGTGTACTTTAC
GTGCTTCCTTTCAGGGACCTCCCCCCGCGTTACTCATTTGTATTCACA
GCTACGAAGTGTCTTGCAAGGTGGATTTCTGCCAGGCTCCATGTCTCA
CTCGTTGCGTTTTCGGAAAAGTTCATTGTGAACGTTCCCCTTGCGTGT
CATGACTTTATCAG

>TgCatBr5
TGCGGCCCCCTTTCCTCCGCTTGCTGTTTAAATGCTCGTCCTCGTTTT
CCTTCCTGCCGCATCCCCGTATATTTTAAGGAGAGGGAAACAGGCGTG
AGTTGGACGGAATGAAAGTTCTCGGCCTGTACGCCGGTTGTCGCGGT
CGTTTGCAGATTGCTTTTTTCTTCGAATCGGTGCTGTAACCCTCGAAG
AAGAACGACGCTGCAAACGACTTGTCGAACTCTCAGTCGTGTACTTTA
CGTGCTTCCTTTCAGGGACCTCCCTCCGCGTTACTCATTTGTATTCAC
AGCTACGAAGTGTCTTGCAAGGTGGATTCGTGCCAGGCTCCATGTCTC
ACTCGGTGCGTTTTCGGAAAAGTTCATTGTGAACGTTCCCCTTGCGT
GTCATGACTTTATCAG

>p89
TGCCGCTCCCTTTCCTCCGCTTGCTGTTTAAATGCTCGTCCTCGTTTT
CCTTCCTGCCGCATCCCCGTATATTTTAAGGAGAGGGAAACAGGCATG
AGTTGGACGAAATGAAAGTTCTCGGCCTGTACGCCGGCTGTCGCGGT
CGTTTGCAGATTGCTTTTTTCTTCGAATCGGTGCTGTAACCCTCGCGA
AGAACGACGCTGCAAACAACTTCTCGAACTCTCAGTCGTGTACTTTAC
GTGCTTCCTTTCAGGGACCTCCCCCCGCGTTACTCATTTGTATTCACA
GCTACGAAGTGTCTTGCAAGGTGGATTCGTGCCAGGCTCCATGTCTCA
CTCGGTGCGTTTTCGGAAAAGTTCATTGTGAACGTTCCCCTTGCGTG
TCATGACTTTATCAG

>VAND
TGCCGCCCCCTTTCCTCCGCTTGCTGTTTAAATGCTCGTCCTCGTTTT
CCTTCCTGCCGCATCCCCGTATATTTTAAGGAGAGGGAAACAGGCGTG
AGTTGGACGGAATGAAAGTTCTCGGCCTGTACGCCGGTTGTCGCGGT

CGTTTGCAGATTGCTTTTTTCTTCGAATCGGTGCTGTAACCCTCGAAG
AAGAACGACGCTGCAAACGACTTCTCGAACTCTCAGTCGTGTACTTTA
CGTGCTTCCTTTCAGGGACCTCCCCCCGCGTTACTCATTTGTATTCAC
AGCTACGAAGTGTCTTGCAAGGTGGATTTCTGCCAGGCTCCATGTCTC
ACTCGGTGCGTTTTCGGAAAAGTTCATTGTGAACGTTCCCCTTGCGT
GTCATGACTTTATCAG

>TgCgCa1
TGCGGCCCCCTTTCCTCCGCTTGCTGTTTAAATGCTCGTCCTCGTTTT
GCTTCCTGCCGCATCCCCGTATATTTTAAGGAGAGGGAAACAGGCGTG
AGTTGGACGGAATGAAAGTTCTCGGCCTGTACGCCCGTTGTCGCGGT
CGTTTGCAGATTGCTTTTTTCTTCGAATCGGTGCTGTAACCCTCGCGA
AGAACGACGCTGCAAACGACTTCTCGAACTCTCAGTCGTGTACTTTAC
GTGCTTCCTTTCAGGGACCTCCCCCCGCGTTACTCATTTGTATTCACA
GCTACGAAGTGTCTTGCAAGGTGGATTTCTGCCAGGCTCCATGTCTCA
CTCGGTGCGTTTTCGGAAAAGTTCATTGTGAACGTTCCCCTTGCGTG
TCATGACTTTATCAG

>ARI
TGCGGCCCCCTTTCCTCCGCTTGCTGTTTAAATGCTCGTCCTCGTTTT
CCTTCCTGCCGCATCCCCGTATATTTTAAGGAGAGGGAAACAGGCGTG
AGTTGGACGGCATGAAAGTTCTCGGCCTGTATGCCGGTTGTGGCGGT
CGTTTGCAGATTGCTTTTTTCTTCGAATCGGTGCTGTAACCCTCGCGA
AGAACGACGCTGCAAACGACTTCTCGAACTCTCAGTCGTGTACTTTAC
GTGCTTCCTTTCAGGGACCTCCCCCCGCGTTACTCATTTGTATTCACA
GCTACGAAGTGTCTTGCAAGGTGGATTTCTGCCAGGCTCCATGTCTCA
CTCGTTGCGTTTTCGGAAAAGTTCATTGTGAACGTTCCCCTTGCGTGT
CATGACTTTATCAG

>TgCtPRC2
TGCCGCCCCCTTTTCTCCGCTTGCTGTTTAAATGCTCGTCCTCGTTTT
CCTTCCTGCCGCATCCCCGTATATTTTAAGGAGAGGGAAACAGGCGTG
AGTTGGACGGAATGAAAGTTCTCGGCCTGTACGCCGGTTGTCGCGGT
CGTTTGCAGATTGCTTTTTTCTTCGAATCGGTGCTGTAACCCTCGCGA
AGAACGACGCTGCAAACGACTTCTCGAACTCTCAGTCGTGTACTTTAC
GTGCTTCCTTTCAGGGACCTCCCCCCGCGTTACTCATTTGTATTCACA

GCTACGAAGTGTCTTGCAAGGTGGATTCGTGCCAGGCTCCATGTCTCA
CTCGGTGCGTTTTCGGAAAAGTTCATTGTGAACGTTCCCCTTGCGTG
TCATGACTTTATCAG

>GAB2-2007-GAL-DOM2
TGCGGCCCCCTTTCCTCCGCTTGCTGTTTAAATGCTCGTCCTCGTTTT
CCTTCCTGCCGCATCCCCGTATATTTTAAGGAGAGGGAAACAGGCGTG
AGTTGGACGGAATGAAAGTTCTCGGCCTGTACGCCGGTTGTCGCGGT
CGTTTGCAGATTGCTTTTTTCTTCGAATCGGTGCTGTAACCCTCGCGA
AGAACGACGCTGCAAACGACTTCTCGAACTCTCAGTCGTGTACTTTAC
GTGCTTCCTTTCAGGGACCTCCCCCCGCGTTACTCATTTGTATTCACA
GCTACGAAGTGTCTTGCAAGGTGGATTCGTGACAGGCTCCATGTCTCA
CTCGGTGCATTTTCGGAAAAGTTCATTGTGAACGTTGCCCTTGCGTGT
CATGACTTTATCAG

>TgCtCo5
TGCCGCTCCCTTTCCTCCGCTTGCTGTTTAAATGCTCGTCCTCGTTTT
CCTTCCTGCCGCATCCCCGTATATTTTAAGGAGAGGGAAACAGGCATG
AGTTGGACGAAATGAAAGTTCTCGGCCTGTACGCCGGCTGTCGCGGT
CGTTTGCAGATTGCTTTTTTCTTCGAATCGGTGCTGTAACCCTCGCGA
AGAACGACGCTGCAAACAACTTCTCGAACTCTCAGTCGTGTACTTTAC
GTGCTTCCTTTCAGGGACCTCCCCCCGCGTTACTCATTTGTATTCACA
GCTACGAAGTGTCTTGCAAGGTGGATTCGTGACAGGCTCCATGTCTCA
CTCGGTGCATTTTCGGAAAAGTTCATTGTGAACGTTCCCCTTGCGTGT
CATGACTTTATCAG

>TgCatJpOk1
GACCAGGAAGAAAGCATTCTCCAGGACATCATCACGAGGTAATCCTTC
AACCGAAGTTTGCTTTCCGTGACTCTGCCTGTTGGTTATACTGCGTGG
CCTTCCCGTCCTGCGGCCCCCTTTCCTCCGCTTGCTGTTTAAATGCTC
GTCCTCGTTTTCCTTCCTGCCGCATCCCCGTATATTTTAAGGAGAGGG
AAACAGGCGTGAGTTGGACGGCATGAAAGTTCTCGGCCTGTATGCCG
GTTGTGGCGGTCGTTTGCAGATTGCTTTTTTCTTCGAATCGGTGCTG
TAACCCTCGCGAAGAACGACGCTGCAAACGACTTCTCGAACTCTCAGT
CGTGTACTTTACGTGCTTCCTTTCAGGGACCTCCCCCCGCGTTACTCA
TTTGTATTCACAGCTACGAAGTGTCTTGCAAGGTGGATTTCTGCCAGG

CTCCATGTCTCACTCGTTGCGTTTTCGGAAAAGTTCATTGTGAACGTT
CCCCTTGCGTGTCATGACTTTATCAGGTTTCCCAATGTGGTG

>TgCatJpOk2
GAAGAAAGCATTCTCCAGGACATCATCACGAGGTAATCCTTCAACCGAA
GTTTGCTTTCCGTGACTCTGCCTGTTGGTTATACTGCGTGGCCTTCCC
GTCCTGCGGCCCCCTTTCCTCCGCTTGCTGTTTAAATGCTCGTCCTC
GTTTTCCTTCCTGCCGCATCCCCGTATATTTTAAGGAGAGGGAAACAG
GCGTGAGTTGGACGGCATGAAAGTTCTCGGCCTGTATGCCGGTTGTG
GCGGTCGTTTGCAGATTGCTTTTTTCTTCGAATCGGTGCTGTAACCCT
CGCGAAGAACGACGCTGCAAACGACTTCTCGAACTCTCAGTCGTGTAC
TTTACGTGCTTCCTTTCAGGGACCTCCCCCCGCGTTACTCATTTGTAT
TCACAGCTACGAAGTGTCTTGCAAGGTGGATTTCTGCCAGGCTCCATG
TCTCACTCGTTGCGTTTTCGGAAAAGTTCATTGTGAACGTTCCCCTTG
CGTGTCATGACTTTATCAGGTTTCCCAATGTGGTGCTCATGAA

>TgCatJpOk3
GGTAATCCTTCAACAGAAGTTTGCTTTCCGTGACTCTGCCTGTTGGTT
ATACTGCGTGGCCTTCCCGTCCTGCGGCCCCCTTTCCTCCGCTTGCT
GTTTAAATGCTCGTCCTCGTTTTCCTTCCTGCCGCATCCCCGTATATT
TTAAGGAGAGGGAAACAGGCGTGAGTTGGACGGCATGAAAGTTCTCG
GCCTGTATGCCGGTTGTGGCGGTCGTTTGCAGATTGCTTTTTTCTTC
GAATCGGTGCTGTAACCCTCGCGAAGAACGACGCTGCAAACGACTTCT
CGAACTCTCAGTCGTGTACTTTACGTGCTTCCTTTCAGGGACCTCCCC
CCGCGTTACTCATTTGTATTCACAGCTACGAAGTGTCTTGCAAGGTGG
ATTTCTGCCAGGCTCCATGTCTCACTCGTTGCGTTTTCGGAAAAGTTC
ATTGTGAACGTTCCCCTTGCGTGTCATGACTTTATCAGGTTTCCCAAT
GTGGTGCTCATGAA

>TgCatJpOk4
ACCGAAGTTTGCTTTCCGTGACTCTGCCTGTTGGTTATACTGCGTGGC
CTTCCCGTCCTGCGGCCCCCTTTCCTCCGCTTGCTGTTTAAATGCTCG
TCCTCGTTTTCCTTCCTGCCGCATCCCCGTATATTTTAAGGAGAGGGA
AACAGGCGTGAGTTGGACGGCATGAAAGTTCTCGGCCTGTATGCCGG
TTGTGGCGGTCGTTTGCAGATTGCTTTTTTCTTCGAATCGGTGCTGT
AACCCTCGCGAAGAACGACGCTGCAAACGACTTCTCGAACTCTCAGTC

GTGTACTTTACGTGCTTCCTTTCAGGGACCTCCCCCCGCGTTACTCAT
TTGTATTCACAGCTACGAAGTGTCTTGCAAGGTGGATTTCTGCCAGGC
TCCATGTCTCACTCGTTGCGTTTTCGGAAAAGTTCATTGTGAACGTTC
CCCTTGCGTGTCATGACTTTATCANGTTTCCCAATGTGGTGCT

>TgCatJpTy1/k-3
CCCGATATTCGACAAACGACCAGGAAGAAAGCATTCTCCAGGACATCAT
CACGAGGTAATCCTTCAACCGAAGTTTGCTTTCCGTGACTCTGCCTGT
TGGTTATACTGCGTGGCCTTCCCGTCCTGCGGCCCCCTTTCCTCCGC
TTGCTGTTTAAATGCTCGTCCTCGTTTTCCTTCCTGCCGCATCCCCGT
ATATTTTAAGGAGAGGGAAACAGGCGTGAGTTGGACGGCATGAAAGTT
CTCGGCCTGTATGCCGGTTGTGGCGGTCGTTTGCAGATTGCTTTTTT
CTTCGAATCGGTGCTGTAACCCTCGnCGAAGAACGACGCTGCAAACGA
CTTCTCGAACTCTCAGTCGTGTACTTTACGTGCTTCCTTTCAGGGACC
TCCCCCCGCGTTACTCATTTGTATTCACAGCTACGAAGTGTCTTGCAA
GGTGGATTTCTGCCAGGCTCCATGTCTCACTCGTTGCGTTTTCGGAA
AAGTTCATTGTGAACGTTCCCCTTGCGTGTCATGACTTTATCAGGTTT
CCCAATGTGGTGCTCATGAAGCAGACGGCTC

>TgCatJpGi1/TaJ
GCAAACGACCAGGAAGAAAGCATTCTCCAGGACATCATCACGAGGTAA
TCCTTCAACCGAAGTTTGCTTTCCGTGACTCTGCCTGTTGGTTATACT
GCGTGGCCTTCCCGTCCTGCGGCCCCCTTTCCTCCGCTTGCTGTTTA
AATGCTCGTCCTCGTTTTCCTTCCTGCCGCATCCCCGTATATTTTAAG
GAGAGGGAAACAGGCGTGAGTTGGACGGCATGAAAGTTCTCGGCCTG
TATGCCGGTTGTGGCGGTCGTTTGCAGATTGCTTTTTTCTTCGAATC
GGTGCTGTAACCCTCGCGAAGAACGACGCTGCAAACGACTTCTCGAAC
TCTCAGTCGTGTACTTTACGTGCTTCCTTTCAGGGACCTCCCCCCGCG
TTACTCATTTGTATTCACAGCTACGAAGTGTCTTGCAAGGTGGATTTC
TGCCAGGCTCCATGTCTCACTCGTTGCGTTTTCGGAAAAGTTCATTGT
GAACGTTCCCCTTGCGTGTCATGACTTTATCAG

>TgGoatJpOk1
CAGGAAGAAAGCATTCTCCAGGACATCATCACGAGGTAATCCTTCAACC
GAAGTTTGCTTTCCGTGACTCTGCCTGTTGGTTATACTGCGTGGCCTT
CCCGTCCTGCGGCCCCCTTTCCTCCGCTTGCTGTTTAAATGCTCGTCC

TCGTTTTCCTTCCTGCCGCATCCCCGTATATTTTAAGGAGAGGGAAAC
AGGCGTGAGTTGGACGGCATGAAAGTTCTCGGCCTGTATGCCGGTTG
TGGCGGTCGTTTGCAGATTGCTTTTTTCTTCGAATCGGTGCTGTAAC
CCTCGCGAAGAACGACGCTGCAAACGACTTCTCGAACTCTCAGTCGTG
TACTTTACGTGCTTCCTTTCAGGGACCTCCCCCCGCGTTACTCATTTG
TATTCACAGCTACGAAGTGTCTTGCAAGGTGGATTTCTGCCAGGCTCC
ATGTCTCACTCGTTGCGTTTTCGGAAAAGTTCATTGTGAACGTTCCCC
TTGCGTGTCATGACTTTATCAGGTTTCCCAATGTGGTGCTCAT

>TgGoatJpOk2
CACGACCAGGAAGAAAGCATTCTCCAGGACATCATCACGAGGTAATCC
TTCAACCGAAGTTTGCTTTCCGTGACTCTGCCTGTTGGTTATACTGCG
TGGCCTTCCCGTCCTGCGGCCCCCTTTCCTCCGCTTGCTGTTTAAAT
GCTCGTCCTCGTTTTCCTTCCTGCCGCATCCCCGTATATTTTAAGGAG
AGGGAAACAGGCGTGAGTTGGACGGCATGAAAGTTCTCGGCCTGTAT
GCCGGTTGTGGCGGTCGTTTGCAGATTGCTTTTTTCTTCGAATCGGT
GCTGTAACCCTCGCGAAGAACGACGCTGCAAACGACTTCTCGAACTCT
CAGTCGTGTACTTTACGTGCTTCCTTTCAGGGACCTCCCCCCGCGTTA
CTCATTTGTATTCACAGCTACGAAGTGTCTTGCAAGGTGGATTTCTGC
CAGGCTCCATGTCTCACTCGTTGCGTTTTCGGAAAAGTTCATTGTGAA
CGTTCCCCTTGCGTGTCATGACTTTATCAGGTTTCCCAATGTGGTGCT
C

>TgGoatJpOk3
ACGACCAGGAAGAAAGCATTCTCCAGGACATCATCACGAgGTAATCCTT
CaACCGAAGTTTGCTTTCCGTGACTCTGCCTGTTGGTTATACTGCGTG
GCCTTCCCGTCCTGCGGCCCCCTTTCCTCCGCTTGCTGTTTAAATGCT
CGTCCTCGTTTTCCTTCCTGCCGCATCCCCGTATATTTTAAGGAGAGG
GAAACAGGCGTGAGTTGGACGGCATGAAAGTTCTCGGCCTGTATGCC
GGTTGTGGCGGTCGTTTGCAGATTGCTTTTTTCTTCGAATCGGTGCT
GTAACCCTCGCGAAGAACGACGCTGCAAACGACTTCTCGAACTCTCAG
TCGTGTACTTTACGTGCTTCCTTTCAGGGACCTCCCCCCGCGTTACTC
ATTTGTATTCACAGCTACGAAGTGTCTTGCAAGGTGGATTTCTGCCAG
GCTCCATGTCTCACTCGTTGCGTTTTCGGAAAAGTTCATTGTGAACGT
TCCCCTTGCGTGTCATGACTTTATCAGGTTTCCCAATGTGGTGCTCAT
G

>TgGoatJpOk4
CACGAGGTAATCCTTCAACCGAAGTTTGCTTTCCGTGACTCTGCCTGT
TGGTTATACTGCGTGGCCTTCCCGTCCTGCGGCCCCCTTTCCTCCGC
TTGCTGTTTAAATGCTCGTCCTCGTTTTCCTTCCTGCCGCATCCCCGT
ATATTTTAAGGAGAGGGAAACAGGCGTGAGTTGGACGGCATGAAAGTT
CTCGGCCTGTATGCCGGTTGTGGCGGTCGTTTGCAGATTGCTTTTTT
CTTCGAATCGGTGCTGTAACCCTCGCGAAGAACGACGCTGCAAACGAC
TTCTCGAACTCTCAGTCGTGTACTTTACGTGCTTCCTTTCAGGGACCT
CCCCCCGCGTTACTCATTTGTATTCACAGCTACgAAGTGTCTTGCAAGG
TGGATTTCTGCCAGGCTCCATGTCTCACTCGTTGCGTTTTCGGAAAAG
TTCATTGTGAACGTTCCCCTTGCGTGTCATGACTTTATCAGGTTTCCC
AATGTGGTGCTCATGAAGCAGACGGCTCT

>TgGoatJpOk5
GACCAGGAAGAAAGCATTCTCCAGGACATCATCACGAGGTAATCCTTC
AACCGAAGTTTGCTTTCCGTGACTCTGCCTGTTGGTTATACTGCGTGG
CCTTCCCGTCCTGCGGCCCCCTTTCCTCCGCTTGCTGTTTAAATGCTC
GTCCTCGTTTTCCTTCCTGCCGCATCCCCGTATATTTTAAGGAGAGGG
AAACAGGCGTGAGTTGGACGGCATGAAAGTTCTCGGCCTGTATGCCG
GTTGTGGCGGTCGTTTGCAGATTGCTTTTTTCTTCGAATCGGTGCTG
TAACCCTCGCGAAGAACGACGCTGCAAACGACTTCTCGAACTCTCAGT
CGTGTACTTTACGTGCTTCCTTTCAGGGACCTCCCCCCGCGTTACTCA
TTTGTATTCACAGCTACGAAGTGTCTTGCAAGGTGGATTTCTGCCAGG
CTCCATGTCTCACTCGTTGCGTTTTCGGAAAAGTTCATTGTGAACGTT
CCCCTTGCGTGTCATGACTTTATCAGGTTTCCCAATGTGGTGCTCA

>TgGoatJpOk6
CGACCAGGAAGAAAGCATTCTCCAGGACATCATCACGAGGTAATCCTT
CaACCGAAGTTTGCTTTCCGTGACTCTGCCTGTTGGTTATACTGCGTG
GCCTTCCCGTCCTGCGGCCCCCTTTCCTCCGCTTGCTGTTTAAATGCT
CGTCCTCGTTTTCCTTCCTGCCGCATCCCCGTATATTTTAAGGAGAGG
GAAACAGGCGTGAGTTGGACGGCATGAAAGTTCTCGGCCTGTATGCC
GGTTGTGGCGGTCGTTTGCAGATTGCTTTTTTCTTCGAATCGGTGCT
GTAACCCTCGCGAAGAACGACGCTGCAAACGACTTCTCGAACTCTCAG
TCGTGTACTTTACGTGCTTCCTTTCAGGGACCTCCCCCCGCGTTACTC

ATTTGTATTCACAGCTACGAAGTGTCTTGCAAGGTGGATTTCTGCCAG
GCTCCATGTCTCACTCGTTGCGTTTTCGGAAAAGTTCATTGTGAACGT
TCCCCTTGCGTGTCATGACTTTATCAGGTTTCCCAATGTGGTGCTCAT
GAA

>TgGoatJpOk7
CAGGAAGAAAGCATTCTCCAGGACATCATCaCGAGGTAATCCTTCAaCC
GAAGTTTGCTTTCCGTGACTCTGCCTGTTGGTTATACTGCGTGGCCTT
CCCGTCCTGCGGCCCCCTTTCCTCCGCTTGCTGTTTAAATGCTCGTCC
TCGTTTTCCTTCCTGCCGCATCCCCGTATATTTTAAGGAGAGGGAAAC
AGGCGTGAGTTGGACGGCATGAAAGTTCTCGGCCTGTATGCCGGTTG
TGGCGGTCGTTTGCAGATTGCTTTTTTCTTCGAATCGGTGCTGTAAC
CCTCGCGAAGAACGACGCTGCAAACGACTTCTCGAACTCTCAGTCGTG
TACTTTACGTGCTTCCTTTCAGGGACCTCCCCCCGCGTTACTCATTTG
TATTCACAGCTACGAAGTGTCTTGCAAGGTGGATTTCTGCCAGGCTCC
ATGTCTCACTCGTTGCGTTTTCGGAAAAGTTCATTGTGAACGTTCCCC
TTGCGTGTCATGACTTTATCAGGTTTCCCAATGTGGTGCTCATGAAGC
AGACGGCTC

>TgGoatJpOk8
GACCAGGAAGAAAGCATTCTCCAGGACATCATCACGAGGTAATCCTTC
AACCGAAGTTTGCTTTCCGTGACTCTGCCTGTTGGTTATACTGCGTGG
CCTTCCCGTCCTGCGGCCCCCTTTCCTCCGCTTGCTGTTTAAATGCTC
GTCCTCGTTTTCCTTCCTGCCGCATCCCCGTATATTTTAAGGAGAGGG
AAACAGGCGTGAGTTGGACGGCATGAAAGTTCTCGGCCTGTATGCCG
GTTGTGGCGGTCGTTTGCAGATTGCTTTTTTCTTCGAATCGGTGCTG
TAACCCTCGCGAAGAACGACGCTGCAAACGACTTCTCGAACTCTCAGT
CGTGTACTTTACGTGCTTCCTTTCAGGGACCTCCCCCCGCGTTACTCA
TTTGTATTCACAGCTACGAAGTGTCTTGCAAGGTGGATTTCTGCCAGG
CTCCATGTCTCACTCGTTGCGTTTTCGGAAAAGTTCATTGTGAACGTT
CCCCTTGCGTGTCATGACTTTATCAGGTTTCCCAATGTGGTGCTCAT

>TgGoatJpOk9
AACGACCAGGAAGAAAGCATTCTCCAGGACATCATCACGAGGTAATCC
TTCaACCGAAGTTTGCTTTCCGTGACTCTGCCTGTTGGTTATACTGCG
TGGCCTTCCCGTCCTGCGGCCCCCTTTCCTCCGCTTGCTGTTTAAAT

GCTCGTCCTCGTTTTCCTTCCTGCCGCATCCCCGTATATTTTAAGGAG
AGGGAAACAGGCGTGAGTTGGACGGCATGAAAGTTCTCGGCCTGTAT
GCCGGTTGTGGCGGTCGTTTGCAGATTGCTTTTTTCTTCGAATCGGT
GCTGTAACCCTCGCGAAGAACGACGCTGCAAACGACTTCTCGAACTCT
CAGTCGTGTACTTTACGTGCTTCCTTTCAGGGACCTCCCCCCGCGTTA
CTCATTTGTATTCACAGCTACGAAGTGTCTTGCAAGGTGGATTTCTGC
CAGGCTCCATGTCTCACTCGTTGCGTTTTCGGAAAAGTTCATTGTGAA
CGTTCCCCTTGCGTGTCATGACTTTATCAGGTTTCCCAATGTGGTGCT
CATGAAGCA

>TgGoatJpOk10
AAACGACCAGGAAGAAAGCATTCTCCAGGACATCATCACGAGGTAATCC
TTCAACCGAAGTTTGCTTTCCGTGACTCTGCCTGTTGGTTATACTGCG
TGGCCTTCCCGTCCTGCGGCCCCCTTTCCTCCGCTTGCTGTTTAAAT
GCTCGTCCTCGTTTTCCTTCCTGCCGCATCCCCGTATATTTTAAGGAG
AGGGAAACAGGCGTGAGTTGGACGGCATGAAAGTTCTCGGCCTGTAT
GCCGGTTGTGGCGGTCGTTTGCAGATTGCTTTTTTCTTCGAATCGGT
GCTGTAACCCTCGCGAAGAACGACGCTGCAAACGACTTCTCGAACTCT
CAGTCGTGTACTTTACGTGCTTCCTTTCAGGGACCTCCCCCCGCGTTA
CTCATTTGTATTCACAGCTACGAAGTGTCTTGCAAGGTGGATTTCTGC
CAGGCTCCATGTCTCACTCGTTGCGTTTTCGGAAAAGTTCATTGTGAA
CGTTCCCCTTGCGTGTCATGACTTTATCAGGTTTCCCAATGTGGTGCT
CAT

>TgGoatJpOk11
CGACCAGGAAGAAAGCATTCTCCAGGACATCATCACGAGGTAATCCTT
CAACCGAAGTTTGCTTTCCGTGACTCTGCCTGTTGGTTATACTGCGTG
GCCTTCCCGTCCTGCGGCCCCCTTTCCTCCGCTTGCTGTTTAAATGCT
CGTCCTCGTTTTCCTTCCTGCCGCATCCCCGTATATTTTAAGGAGAGG
GAAACAGGCGTGAGTTGGACGGCATGAAAGTTCTCGGCCTGTATGCC
GGTTGTGGCGGTCGTTTGCAGATTGCTTTTTTCTTCGAATCGGTGCT
GTAACCCTCGCGAAGAACGACGCTGCAAACGACTTCTCGAACTCTCAG
TCGTGTACTTTACGTGCTTCCTTTCAGGGACCTCCCCCCGCGTTACTC
ATTTGTATTCACAGCTACGAAGTGTCTTGCAAGGTGGATTTCTGCCAG
GCTCCATGTCTCACTCGTTGCGTTTTCGGAAAAGTTCATTGTGAACGT
TCCCCTTGCGTGTCATGACTTTATCAGGTTTCCCAATGTGGTGCTCAT

Uracil phosphoribosyl transferase intron 7 (UPRT7)

>GT1
GTAAGAAATAACGCTTTGAAGGTCGTTGGCTTGTATTACCGTCACTAC
TGCGACGACATACTGGAGAACTCGAGCATCCTTTATGGGTTGGTGTTG
ATGAACGTAGGACTACCAGATGTCATTTCTTCATTGGAACACTTTATTG
TACACGCATGCCGTTTATGCGCTGCTTATCCGAGGGTGGCATCACAGG
GACCCTACAAGAAGCTGACGGGGCACCTCCACTATGAAGTGACATGCC
AATGCACGCCCAGGTTGCATGTTCCAAGTGTCTGGTATCTTTGGCTAC
GCTGTCTTTTACTAATCGCAAGAAACAGTGCTCGTCATGGGTGGTAAC
TCCTTGGTGCCACCAGAACGCATGTGGTGCGTTTGTTGTTCCGCTTT
GTTTTCTTGGGAGATTTCGCTGCGGCCTAATGCAGCCAGCCCTTTATG
GAATTGTGTGCCCTGCTGTCTTGTCAG

>ME49
TCTTGTTTGCTTTCCTCGGCGGTCTGCAGTGTTGACATCTGCCTGAA
CTCGAGGTACTACATCGTCCCCGGCATTGGTGATTTCGGTGACCGGTA
AGAAATAACGCTTTGAAGGTCGTTGGCTTGTATTACCGTCACTACTGC
GACGACATACTGGAGAACTCGAGCATCCTTTATGGGTTTGTGTTGATG
AACGTAGGACTACCAGATATCATTTCTTCATTGGAACACTTTATTGTAC
ACGCATGCCGTTTATGCGCTGCTTATCCGAGGGTGGCATCACAGGGAC
CCTACAAGAAGCTGACGGGGCACCTCCACTATGAAGTGACATGCCAAT
GCATGCCCAGGTTGCATGTTCCAAGTGTCTGGTATCTTTGGCTACGCT
GTCTTTTACTAATCGCAAGAAACAGTGCTCGTCATGGGTGGTAACTCC
TTGGTGCCACCAGAACGCATGTGGTGCGTTTGTTGTTCCGCTTTGTT
TTCTTGGGAGATTTCGCTGCGGCCTAATGCAGCCAACCCTTTATGGAA
TTGTGTGCCCTGCTGTCTTGTCAGGTACTTTGGAACCATGTAGGTCTC
TAGTTTTTTTGACAGACCGCTGACGG

>VEG
GTAAGAAATAACGCTTTGAAGGTCGTTGGCTTGTATTACCGTCACTAC
TGCGACGACATACTGGAGAACTCGAGCATCCTTTATGGGTTTGTGTTG
ATGAACGTAGGACTACCAGATATCATTTCTTCATTGGAACACTTTATTG
TACACGCATGCCGTTTATGCGCTGCTTATCCGAGGGTGGCATCACAGG
GACCCTACAAGAAGCTGACGGGGCACCTCCACTATGAAGTGACATGCC
AATGCATGCCCAGGTTGCATGTTCCAAGTGTCTGGTATCTTTGGCTAC

GCTGTCTTTTACTAATCGCAAGAAACAGTGCTCGTCATGGGTGGTAAC
TCCTTGGTGCCACCAGAACGCATGTGGTGCGTTTGTTGTTCCGCTTT
GTTTTCTTGGGAGATTTCGCTGCGGCCTAATGCAGCCAACCCTTTATG
GAATTGTGTGCCCTGCTGTCTTGTCAG

>MAS
GTAAGAAATAACGCTTTGAAGGTCGTTGGCTTGTATTACCGTCACTAC
TGCGACGACATACTGGAGAACTCGAGCATCCTTTATGGGTTTGTGTTG
ATGAACGTAGGACTACCAGATGTCATTTCTTCATTGGAACACTTTATTG
TACACGCATGCCGTTTATGCGCTGCTTATCCGAGGGTGGCATCACAGG
GACCCTACAAGAAGCTGACGGGGCACCTCCACTATGAAGTGACATGCC
AATGCATGCCCAGGTTGCATGTTCCAAGTGTCTGGTATCTTTGGCTAC
GCTGTCTTTTACTAATCGCAAGAAGCAGTGCTCGTCATGGATGGTAAC
TCCTTGGTGCCACCAGAACGCATGTGGTGCGTTTGTTGTTCCGCTTT
GTTTTCTTGGGAGATTTCGCTGCGGCCTAATGCAGCCAGCCCTTTATG
GAATTGTGTGCCCTGCTGTCTTGTCAG

>RUB
GTAAGAAATAACGCTTTGAAGGTCGTTGGCTTGTATTATCGTCACTAC
TGCGACGACATACTGGAGAACTCGAGCATCCTTTATGGGTTTGTGTTG
ATGAACGTAGGACTAACAGATGTCATTTCTTCATTGGAACACTTTATTG
TACACGCATGCCGTTTATGCGCTGCTTATCCGAGGGTGGCATCACAGG
GACCCTACAAGAAGCTGACGGGGCACCTCCACTATGAAGTGACATGCC
AATTCATGCCCAGGTTGCATGTTCCAAGTGTCTGGTATCTTTGGCTAC
GCTGTCTTTTACTAATCGCAAGAAACAGTGCTCGTCATGGGTGGTAAC
TCCTTGGTGCCACCAGAACGCATGTGGTGCGTTTGTTGTTCCGCTTT
GTTTTCTTGGGAGATTTCGCTGCGGCCTAATGCAGCCAGCCCTTTATG
GAATTGTGTGCCCTGTTGTCTTGTCAG

>FOU
GTAAGAAATAACGCTTTGAAGGTCGTTGGCTTGTATTACCGTCACTAC
TGCGACGACATACTGGAGAACTCGAGCATCCTTTATGGGTTTGTGTTG
ATGAACGTAGGACTACCAGATGTCATTTCTTCATTGGAACACTTTATTG
TACACGCATGCCGTTTATGCGCTGCTTATCCGAGGGTGGCATCACAGG
GACCCTACAAGAAGCTGACGGGGCACCTCCACTATGAAGTGACATGCC
AATGCATGCCCAGGTTGCATGTTCCAAGTGTCTGGTATCTTTGGCTAC

GCTGTCTTTTACTAATCGCAAGAAACAGTGCTCGTCATGGGTGGTAAC
TCCTTGGTGCCACCAGAACGCATGTGGTGCGTTTGTTGTTCCGCTTT
GTTTTCTTGGGAGATTTCGCTGCGGCCTAATGCAGCCAGCCCTTTATG
GAATTGTGTGCCCTGCTGTCTTGTCAG

>CAST
GTAAGAAATAACGCTTTGAAGGTCGTTGGCTTGTATTACCGTCACTAC
TGCGACGACATACTGGAGAACTCGAGCATCCTTTATGGGTTTGTGTTG
ATGAACGTAGGACTAACAGATGTCATTTCTTCATTGGAACACTTTATTG
TACACGCATGCCGTTTATGCGCTGCTTATCCGAGGGTGGCATCACAGG
GACCCTACAAGAACCTGACGGGGCACCTCCACTATGAAGTGACATGCC
AATGCATGCCCAGGTTGCATGTTCCAAGTGTCTGGTATCTTTGGCTAC
GCTGTCTTTTACTAATCGCAAGAAACAGTGCTCGTCATGGGTGGTAAC
TCCTTGGTGCCACCAGAACACATGTGGCGCGTTTGTTGTTCCGCTTT
GTTTTCTTGGGAGATTTCGCTGCGGCCTAATGCAGCCAACCCTTTATG
GAATTGTGTGCCATGCTGTCTTGTCAG

>TgCatBr5
GTAAGAAATAACGCTTTGAAGGTCGTTGGCTTGTATTACCGTCACTAC
TGCGACGACATACTGGAGAACTCGAGCATCCTTTATGGGTTTGTGTTG
ATGAACGTAGGACTACCAGATGTCATTTCTTCATTGGAACACTTTATTG
TACACGCATGCCGTTTATGCGCTGCTTATCCGAGGGTGGCATCACAGG
GACCCTACAAGAAGCTGACGGGGCACCTCCACTATGAAGTGACATGCC
AATGCATGCCCAGGTTGCATGTTCCAAGTGTCTGGTATCTTTGGCTAC
GCTGTCTTTTACTAATCGCAAGAAGCAGTGCTCGTCATGGATGGTAAC
TCCTTGGTGCCACCAGAACGCATGTGGTGCGTTTGTTGTTCCGCTTT
GTTTTCTTGGGAGATTTCGCTGCGGCCTAATGCAGCCAGCCCTTTATG
GAATTGTGTGCCCTGCTGTCTTGTCAG

>p89
GTAAGAAATAACGCTTTGAAGGTCGTTGGCTTGTATTACCGTCACTAC
TGCGACGACATACTGGAGAACTCGAGCATCCTTTATGGGTTTGTGTTG
ATGAACGTAGGACTAACAGATGTCATTTCTTCATTGGAACACTTTATTG
TACACGCATGCCGTTTATGCGCTGCTTATCCGAGGGTGGCATCACAGG
GACCCTACAAGAAGCTGACGGGGCACCTCCACTATGAAGTGACATGCC
AATGCACGCCCAGGTTGCATGTTCCAAGTGTCTGGTATCTTTGGCTAC

GCTGTCTTTTACTAATCGCAAGAAACAGTGCTCGTCATGGGTGGTAAC
TCCTTGGTGCCACCAGAACGCATGTGGCGCGTTTGTTGTTCCGCTTT
GTTTTCTTGGGAGATTTCGCTGCGGCCTAATGCAGCCAGCCCTTTATG
GAATTGTGTGCCCTGCTGTCTTGTCAG

>VAND
GTAAGAAATAACGCTTTGAAGGTCGTTGGCTTGTATTACCGTCACTAC
TGCGACGACATACTGGAGAACTCGAGCATCCTTTATGGGTTTGTGTTG
ATGAACGTAGGACTAACAGATGTCATTTCTTCATTGGAACACTTTATTG
TACACGCATGCCGTTTATGCGCTGCTTATCCGAGGGTGGCATCACAGG
GACCCTACAAGAAGCTGACGGGGCACCTCCACTATGAAGTGACATGCC
AATGCATGCCCAGGTTGCATGTTCCAAGTGTCTGGTATCTTTGGCTAC
GCTGTCTTTTACTAATCGCAAGAAACAGTGCTCGTCATGGGTGGTAAC
TCCTTGGTGCCACCAGAACGCATGTGGTGCGTTTGTTGTTCCGCTTT
GTTTTCTTGGGAGATTTCGCTGCGGCCTAATGCAGCCAGCCCTTTATG
GAATTGTGTGCCCTGCTGTCTTGTCAG

>TgCgCa1
GTAAGAAATAACGCTTTGAAGGTCGTTGGCTTGTATTACCGTCACTAC
TGCGACGACATACTGGAGAACTCGAGCATCCTTTATGGGTTTGTGTTG
ATGAACGTAGGACTACCAGATGTCATTTCTTCATTGGAACACTTTATTG
TACACGCATGCCGTTTATGCGCTGCTTATCCGAGGGTGGCATCACAGG
GACCCTACAAGAAGCTGACGGGGCACCTCCACTATGAAGTGACATGCC
AATGCATGCCCAGGTTGCATGTTCCAAGTGTCTGGTATCTTTGGCTAC
GCTGTCTTTTACTAATCGCAAGAAACAGTGCTTGTCATGGGTGGTAAC
TCCTTGGTGCCACCAGAACGCATGTGGTGCGTTTGTTGTTCCGCTTT
GTTTTCTTGGGAGATTTCGCTGCGGCCTAATGCAGCCAACCCTTTATG
GAATTGTGTGCCCTGCTGTCTTGTCAG

>ARI
GTAAGAAATAACGCTTTGAAGGTCGTTGGCTTGTATTACCGTCACTAC
TGCGACGACATACTGGAGAACTCGAGCATCCTTTATGGGTTTGTGTTG
ATGAACGTAGGACTACCAGATATCATTTCTTCATTGGAACACTTTATTG
TACACGCATGCCGTTTATGCGCTGCTTATCCGAGGGTGGCATCACAGG
GACCCTACAAGAAGCTGACGGGGCACCTCCACTATGAAGTGACATGCC
AATGCATGCCCAGGTTGCATGTTCCAAGTGTCTGGTATCTTTGGCTAC

GCTGTCTTTTACTAATCGCAAGAAACAGTGCTCGTCATGGGTGGTAAC
TCCTTGGTGCCACCAGAACGCATGTGGTGCGTTTGTTGTTCCGCTTT
GTTTTCTTGGGAGATTTCGCTGCGGCCTAATGCAGCCAACCCTTTATG
GAATTGTGTGCCCTGCTGTCTTGTCAG

>TgCtPRC2
GTAAGAAATAACGCTTTGAAGGTCGTTGGCTTGTATTATCGTCACTAC
TGCGACGACATACTGGAGAACTCGAGCATCCTTTATGGGTTTGTGTTG
ATGAACGTAGGACTAACAGATGTCATTTCTTCATTGGAACACTTTATTG
TACACGCATGCCGTTTATGCGCTGCTTATCCGAGGGTGGCATCACAGG
GACCCTACAAGAAGCTGACGGGGCACCTCCACTATGAAGTGACATGCC
AATTCATGCCCAGGTTGCATGTTCCAAGTGTCTGGTATCTTTGGCTAC
GCTGTCTTTTACTAATCGCAAGAAACAGTGCTCGTCATGGGTGGTAAC
TCCTTGGTGCCACCAGAACGCATGTGGTGCGTTTGTTGTTCCGCTTT
GTTTTCTTGGGAGATTTCGCTGCGGCCTAATGCAGCCAGCCCTTTATG
GAATTGTGTGCCCTGCTGTCTTGTCAG

>GAB2-2007-GAL-DOM2
GTAAGAAATAACGCTTTGAAGGTCGTTGGCTTGTATTACCGTCACTAC
TGCGACGACATACTGGAGAACTCGAGCATCCTTTATGGGTTTGTGTTG
ATGAACGTAGGACTACCAGATGTCATTTCTTCATTGGAACACTTTATTG
TACACGCATGCCGTTTATGCGCTGCTTATCCGAGGGTGGCATCACAGG
GACCCTACAAGAAGCTGACGGGGCACCTCCACTATGAAGTGACATGCC
AATGCATGCCCAGGTTGCATGTTCCAAGTGTCTGGTATCTTTGGCTAC
GCTGTCTTTTACTAATCGCAAGAAACAGTGCTCGTCATGGGTGGTAAC
TCCTTGGTGCCACCAGAACGCATGTGGTGCGTTTGTTGTTCCGCTTT
GTTTTCTTGGGAGATTTCGCTGCGGCCTAATGCAGCCAGCCCTTTATG
GAATTGTGTGCCCTGCTGTCTTGTCAG

>TgCtCo5
GTAAGAAATAACGCTTTGAAGGTCGTTGGCTTGTATTACCGTCACTAC
TGCGACGACATACTGGAGAACTCGAGCATCCTTTATGGGTTTGTGTTG
ATGAACGTAGGACTAACAGATGTCATTTCTTCATTGGAACACTTTATTG
TACACGCATGCCGTTTATGCGCTGCTTATCCGAGGGTGGCATCACAGG
GACCCTACAAGAAGCTGACGGGGCACCTCCACTATGAAGTGACATGCC
AATGCATGCCCAGGTTGCATGTTCCAAGTGTCTGGTATCTTTGGCTAC

GCTGTCTTTTACTAATCGCAAGAAACAGTGCTCGTCATGGGTGGTAAC
TCCTTGGTGCCACCAGAACGCATGTGGTGCGTTTGTTGTTCCGCTTT
GTTTTCTTGGGAGATTTCGCTGCGGCCTAATGCAGCCAGCCCTTTATG
GAATTGTGTGCCCTGCTGTCTTGTCAG

>TgCatJpOk1
TCTGCAGTGTTGACATCTGCCTGAACTCGAGGTACTACATCGTCCCCG
GCATTGGTGATTTCGGTGACCGGTAAGAAATAACGCTTTGAAGGTCGT
TGGCTTGTATTACCGTCACTACTGCGACGACATACTGGAGAACTCGAG
CATCCTTTATGGGTTTGTGTTGATGAACGTAGGACTACCAGATATCAT
TTCTTCATTGGAACACTTTATTGTACACGCATGCCGTTTATGCGCTGC
TTATCCGAGGGTGGCATCACAGGGACCCTACAAGAAGCTGACGGGGCA
CCTCCACTATGAAGTGACATGCCAATGCATGCCCAGGTTGCATGTTCC
AAGTGTCTGGTATCTTTGGCTACGCTGTCTTTTACTAATCGCAAGAAA
CAGTGCTCGTCATGGGTGGTAACTCCTTGGTGCCACCAGAACGCATGT
GGTGCGTTTGTTGTTCCGCTTTGTTTTCTTGGGAGATTTCGCTGCGG
CCTAATGCAGCCAACCCTTTATGGAATTGTGTGCCCTGCTGTCTTGTC
AGGTACTTTGGAACCATGTAGGTCTCTAGTTTTT

>TgCatJpOk2
CTTTCCTCGGCGGTCTGCAGTGTTGACATCTGCCTGAACTCGAGGTAC
TACATCGTCCCCGGCATTGGTGATTTCGGTGACCGGTAAGAAATAACG
CTTTGAAGGTCGTTGGCTTGTATTACCGTCACTACTGCGACGACATAC
TGGAGAACTCGAGCATCCTTTATGGGTTTGTGTTGATGAACGTAGGAC
TACCAGATATCATTTCTTCATTGGAACACTTTATTGTACACGCATGCCG
TTTATGCGCTGCTTATCCGAGGGTGGCATCACAGGGACCCTACAAGAA
GCTGACGGGGCACCTCCACTATGAAGTGACATGCCAATGCATGCCCAG
GTTGCATGTTCCAAGTGTCTGGTATCTTTGGCTACGCTGTCTTTTACT
AATCGCAAGAAACAGTGCTCGTCATGGGTGGTAACTCCTTGGTGCCAC
CAGAACGCATGTGGTGCGTTTGTTGTTCCGCTTTGTTTTCTTGGGAG
ATTTCGCTGCGGCCTAATGCAGCCAACCCTTTATGGAATTGTGTGCCC
TGCTGTCTTGTCAGGTACTTTGGAACCATGTAGGTCTCTAGTTTTTTT
GACAGACCGCTGACGGAATCGCGGACTGGAAGAAGCACGCCTCAAGA
GGATAAACACCATTTTTATTTTTGTCTCGCTCCCACGTCCCTCACCATC
GGCAGGAACACAGAAAAAAAGTTTTAGTTGTGAGGCTGCCTGGTGCG
CGACAAGCAGTTGCGCTTTTTTGTGCCACGGGGTATATCT

>TgCatJpOk3
TTTCTGCCTCTTGTTTGCTTTCCTCGGCGGTCTGCAGTGTTGACATC
TGCCTGAACTCGAGGTACTACATCGTCCCCGGCATTGGTGATTTCGGT
GACCGGTAAGAAATAACGCTTTGAAGGTCGTTGGCTTGTATTACCGTC
ACTACTGCGACGACATACTGGAGAACTCGAGCATCCTTTATGGGTTTG
TGTTGATGAACGTAGGACTACCAGATATCATTTCTTCATTGGAACACTT
TATTGTACACGCATGCCGTTTATGCGCTGCTTATCCGAGGGTGGCATC
ACAGGGACCCTACAAGAAGCTGACGGGGCACCTCCACTATGAAGTGAC
ATGCCAATGCATGCCCAGGTTGCATGTTCCAAGTGTCTGGTATCTTTG
GCTACGCTGTCTTTTACTAATCGCAAGAAACAGTGCTCGTCATGGGTG
GTAACTCCTTGGTGCCACCAGAACGCATGTGGTGCGTTTGTTGTTCC
GCTTTGTTTTCTTGGGAGATTTCGCTGCGGCCTAATGCAGCCAACCCT
TTATGGAATTGTGTGCCCTGCTGTCTTGTCAGGTACTTTGGAACCATG
TAGGTCTCTAGTTTTTTTGACAGACCGCTGACGGAATCGCGGACTGGA
AGAAGCACGCCTCAAGAGGATAAACACCATTTTTATTTTTGTCTCGCTC
CCACGTCCCTCACCATCGGCAGGAACACAGAAAAAAAGTTTTAGTTGT
GAGGCTGCCTGGTGCGCGACAAGCAGTTGCGCTTTTTTGTGCCACGG
GGTATATCTTTGGTCGCGCTCCTGTC

>TgCatJpOk4
GGCGGTCTGCAGTGTTGACATCTGCCTGAACTCGAGGTACTACATCGT
CCCCGGCATTGGTGATTTCGGTGACCGGTAAGAAATAACGCTTTGAAG
GTCGTTGGCTTGTATTACCGTCACTACTGCGACGACATACTGGAGAAC
TCGAGCATCCTTTATGGGTTTGTGTTGATGAACGTAGGACTACCAGAT
ATCATTTCTTCATTGGAACACTTTATTGTACACGCATGCCGTTTATGCG
CTGCTTATCCGAGGGTGGCATCACAGGGACCCTACAAGAAGCTGACGG
GGCACCTCCACTATGAAGTGACATGCCAATGCATGCCCAGGTTGCATG
TTCCAAGTGTCTGGTATCTTTGGCTACGCTGTCTTTTACTAATCGCAA
GAAACAGTGCTCGTCATGGGTGGTAACTCCTTGGTGCCACCAGAACGC
ATGTGGTGCGTTTGTTGTTCCGCTTTGTTTTCTTGGGAGATTTCGCT
GCGGCCTAATGCAGCCAACCCTTTATGGAATTGTGTGCCCTGCTGTCT
TGTCAGGTACTTTGGAACCATGTAAGTCTCTAGTTTTTTTG

>TgCatJpTy1/k-3
TCTTGTTTGCTTTCCTCGGCGGTCTGCAGTGTTGACATCTGCCTGAA

CTCGAGGTACTACATCGTCCCCGGCATTGGTGATTTCGGTGACCGGTA
AGAAATAACGCTTTGAAGGTCGTTGGCTTGTATTACCGTCACTACTGC
GACGACATACTGGAGAACTCGAGCATCCTTTATGGGTTTGTGTTGATG
AACGTAGGACTACCAGATATCATTTCTTCATTGGAACACTTTATTGTAC
ACGCATGCCGTTTATGCGCTGCTTATCCGAGGGTGGCATCACAGGGAC
CCTACAAGAAGCTGACGGGGCACCTCCACTATGAAGTGACATGCCAAT
GCATGCCCAGGTTGCATGTTCCAAGTGTCTGGTATCTTTGGCTACGCT
GTCTTTTACTAATCGCAAGAAACAGTGCTCGTCATGGGTGGTAACTCC
TTGGTGCCACCAGAACGCATGTGGTGCGTTTGTTGTTCCGCTTTGTT
TTCTTGGGAGATTTCGCTGCGGCCTAATGCAGCCAACCCTTTATGGAA
TTGTGTGCCCTGCTGTCTTGTCAGGTACTTTGGAACCATGTAGGTCTC
TAGTTTTTTTGACAGACCGCTGA

>TgCatJpGi1/TaJ
TCCTCGGCGGTCTGCAGTGTTGACATCTGCCTGAACTCGAGGTACTAC
ATCGTCCCCGGCATTGGTGATTTCGGTGACCGGTAAGAAATAACGCTT
TGAAGGTCGTTGGCTTGTATTACCGTCACTACTGCGACGACATACTGG
AGAACTCGAGCATCCTTTATGGGTTTGTGTTGATGAACGTAGGACTAC
CAGATATCATTTCTTCATTGGAACACTTTATTGTACACGCATGCCGTTT
ATGCGCTGCTTATCCGAGGGTGGCATCACAGGGACCCTACAAGAAGCT
GACGGGGCACCTCCACTATGAAGTGACATGCCAATGCATGCCCAGGTT
GCATGTTCCAAGTGTCTGGTATCTTTGGCTACGCTGTCTTTTACTAAT
CGCAAGAAACAGTGCTCGTCATGGGTGGTAACTCCTTGGTGCCACCAG
AACGCATGTGGTGCGTTTGTTGTTCCGCTTTGTTTTCTTGGGAGATT
TCGCTGCGGCCTAATGCAGCCAACCCTTTATGGAATTGTGTGCCCTGC
TGTCTGTCAGGTA

>TgGoatJpOk1
TGCAGTGTTGACATCTGCCTGAACTCGAGGTACTACATCGTCCCCCGG
CATTGGTGATTTCGGTGACCGGTAAGAAATAACGCTTTGAAGGTCGTT
GGCTTGTATTACCGTCACTACTGCGACGACATACTGGAGAACTCGAGC
ATCCTTTATGGGTTTGTGTTGATGAACGTAGGACTACCAGATATCATT
TCTTCATTGGAACACTTTATTGTACACGCATGCCGTTTATGCGCTGCT
TATCCGAGGGTGGCATCACAGGGACCCTACAAGAAGCTGACGGGGCAC
CTCCACTATGAAGTGACATGCCAATGCATGCCCAGGTTGCATGTTCCA
AGTGTCTGGTATCTTTGGCTACGCTGTCTTTTACTAATCGCAAGAAAC

AGTGCTCGTCATGGGTGGTAACTCCTTGGTGCCACCAGAACGCATGTG
GTGCGTTTGTTGTTCCGCTTTGTTTTCTTGGGAGATTTCGCTGCGGC
CTAATGCAGCCAACCCTTTATGGAATTGTGTGCCCTGCTGTCTTGTCA
GGTACTTTGGAACCATGTAAGTCTCTAGTTTTTTT

>TgGoatJpOk2
ATCTGCCTGAACTCGAGGTACTACATCGTCCCCGGCATTGGTGATTTC
GGTGACCGGTAAGAAATAACGCTTTGAAGGTCGTTGGCTTGTATTACC
GTCACTACTGCGACGACATACTGGAGAACTCGAGCATCCTTTATGGGT
TTGTGTTGATGAACGTAGGACTACCAGATATCATTTCTTCATTGGAACA
CTTTATTGTACACGCATGCCGTTTATGCGCTGCTTATCCGAGGGTGGC
ATCACAGGGACCCTACAAGAAGCTGACGGGGCACCTCCACTATGAAGT
GACATGCCAATGCATGCCCAGGTTGCATGTTCCAAGTGTCTGGTATCT
TTGGCTACGCTGTCTTTTACTAATCGCAAGAAACAGTGCTCGTCATGG
GTGGTAACTCCTTGGTGCCACCAGAACGCATGTGGTGCGTTTGTTGT
TCCGCTTTGTTTTCTTGGGAGATTTCGCTGCGGCCTAATGCAGCCAAC
CCTTTATGGAATTGTGTGCCCTGCTGTCTTGTCAG

>TgGoatJpOk3
GTGTTGACATCTGCCTGAACTCGAGGTANTACATCGTCCCCGGCATTG
GTGATTTCGGTGACCGGTAAGAAATAACGCTTTGAAGGTCGTTGGCTT
GTATTACCGTCACTACTGCGACGACATACTGGAGAACTCGAGCATCCTT
TATGGGTTTGTGTTGATGAACGTAGGACTACCAGATATCATTTCTTCA
TTGGAACACTTTATTGTACACGCATGCCGTTTATGCGCTGCTTATCCG
AGGGTGGCATCACAGGGACCCTACAAGAAGCTGACGGGGCACCTCCAC
TATGAAGTGACATGCCAATGCATGCCCAGGTTGCATGTTCCAAGTGTC
TGGTATCTTTGGCTACGCTGTCTTTTACTAATCGCAAGAAACAGTGCT
CGTCATGGGTGGTAACTCCTTGGTGCCACCAGAACGCATGTGGTGCG
TTTGTTGTTCCGCTTTGTTTTCTTGGGAGATTTCGCTGCGGCCTAAT
GCAGCCAACCCTTTATGGAATTGTGTGCCCTGCTGTCTTGTCAGG

>TgGoatJpOk4
CTGCCTGAACTCGAGGTACTACATCGTCCCCGGCATTGGTGATTTCGG
TGACCGGTAAGAAATAACGCTTTGAAGGTCGTTGGCTTGTATTACCGT
CACTACTGCGACGACATACTGGAGAACTCGAGCATCCTTTATGGGTTT
GTGTTGATGAACGTAGGACTACCAGATATCATTTCTTCATTGGAACACT

TTATTGTACACGCATGCCGTTTATGCGCTGCTTATCCGAGGGTGGCAT
CACAGGGACCCTACAAGAAGCTGACGGGGCACCTCCACTATGAAGTGA
CATGCCAATGCATGCCCAGGTTGCATGTTCCAAGTGTCTGGTATCTTT
GGCTACGCTGTCTTTTACTAATCGCAAGAAACAGTGCTCGTCATGGGT
GGTAACTCCTTGGTGCCACCAGAACGCATGTGGTGCGTTTGTTGTTC
CGCTTTGTTTTCTTGGGAGATTTCGCTGCGGCCTAATGCAGCCAACCC
TTTATGGAATTGTGTGCCCTGCTGTCTTGTCAGG

>TgGoatJpOk5
TGANTTCGGTGACCGGTAAGAAATAACGCTTTGAAGGTCGTTGGCTT
GTATTACCGTCACTACTGCGACGACATACTGGAGAACTCGAGCATCCTT
TATGGGTTTGTGTTGATGAACGTAGGACTACCAGATATCATTTCTTCA
TTGGAACACTTTATTGTACACGCATGCCGTTTATGCGCTGCTTATCCG
AGGGTGGCATCACAGGGACCCTACAAGAAGCTGACGGGGCACCTCCAC
TATGAAGTGACATGCCAATGCATGCCCAGGTTGCATGTTCCAAGTGTC
TGGTATCTTTGGCTACGCTGTCTTTTACTAATCGCAAGAAACAGTGCT
CGTCATGGGTGGTAACTCCTTGGTGCCACCAGAACGCATGTGGTGCG
TTTGTTGTTCCGCTTTGTTTTCTTGGGAGATTTCGCTGCGGCCTAAT
GCAGCCAACCCTTTATGGAATTGTGTGCCCTGCTGTCTTGTCAGGTAC
TT

>TgGoatJpOk6
TACATCGTCCCCGGCATTGGTGATTTCGGTGACCGGTAAGAAATAACG
CTTTGAAGGTCGTTGGCTTGTATTACCGTCACTACTGCGACGACATAC
TGGAGAACTCGAGCATCCTTTATGGGTTTGTGTTGATGAACGTAGGAC
TACCAGATATCATTTCTTCATTGGAACACTTTATTGTACACGCATGCCG
TTTATGCGCTGCTTATCCGAGGGTGGCATCACAGGGACCCTACAAGAA
GCTGACGGGGCACCTCCACTATGAAGTGACATGCCAATGCATGCCCAG
GTTGCATGTTCCAAGTGTCTGGTATCTTTGGCTACGCTGTCTTTTACT
AATCGCAAGAAACAGTGCTCGTCATGGGTGGTAACTCCTTGGTGCCAC
CAGAACGCATGTGGTGCGTTTGTTGTTCCGCTTTGTTTTCTTGGGAG
ATTTCGCTGCGGCCTAATGCAGCCAACCCTTTATGGAATTGTGTGCCC
TGCTGTCTTGTCAGGTACTTTGGAACCATGTANNTCTCTAGTTT

>TgGoatJpOk7
TCTGCCTGAACTCGAGGTACTACATCGTCCCCGGCATTGGTGATTTCG

GTGACCGGTAAGAAATAACGCTTTGAAGGTCGTTGGCTTGTATTACCG
TCACTACTGCGACGACATACTGGAGAACTCGAGCATCCTTTATGGGTT
TTGTGTTGATGAACGTAGGACTACCAGATATCATTTCTTCATTGGAACA
CTTTATTGTACACGCATGCCGTTTATGCGCTGCTTATCCGAGGGTGGC
ATCACAGGGACCCTACAAGAAGCTGACGGGGCACCTCCACTATGAAGT
GACATGCCAATGCATGCCCAGGTTGCATGTTCCAAGTGTCTGGTATCT
TTGGCTACGCTGTCTTTTACTAATCGCAAGAAACAGTGCTCGTCATGG
GTGGTAACTCCTTGGTGCCACCAGAACGCATGTGGTGCGTTTGTTGT
TCCGCTTTGTTTTCTTGGGAGATTTCGCTGCGGCCTAATGCAGCCAAC
CCTTTATGGAATTGTGTGCCCTGCTGTCTTGTCAGGTACTTTGGAACC
ATG

>TgGoatJpOk8
TTCTTGTTTGCTTTCCTNGNNNGTCTGCAGTGTTGACATCTGCCTGA
ACTCGAGGTACTACATCGTCCCCGGCATTGGTGATTTCGGTGACCGGT
AAGAAATAACGCTTTGAAGGTCGTTGGCTTGTATTACCGTCACTACTG
CGACGACATACTGGAGAACTCGAGCATCCTTTATGGGTTTGTGTTGAT
GAACGTAGGACTACCAGATATCATTTCTTCATTGGAACACTTTATTGTA
CACGCATGCCGTTTATGCGCTGCTTATCCGAGGGTGGCATCACAGGGA
CCCTACAAGAAGCTGACGGGGCACCTCCACTATGAAGTGACATGCCAA
TGCATGCCCAGGTTGCATGTTCCAAGTGTCTGGTATCTTTGGCTACGC
TGTCTTTTACTAATCGCAAGAAACAGTGCTCGTCATGGGTGGTAACTC
CTTGGTGCCACCAGAACGCATGTGGTGCGTTTGTTGTTCCGCTTTGT
TTTCTTGGGAGATTTCGCTGCGGCCTAATGCAGCCAACCCTTTATGGA
ATTGTGTGCCCTGCTGTCTTGTCAGG

>TgGoatJpOk9
TTNCAGTGTTGACATCTGCCTGAACTCGAGGTACTACATCGTCCCCGG
CATTGGTGATTTCGGTGACCGGTAAGAAATAACGCTTTGAAGGTCGTT
GGCTTGTATTACCGTCACTACTGCGACGACATACTGGAGAACTCGAGC
ATCCTTTATGGGTTTGTGTTGATGAACGTAGGACTACCAGATATCATT
TCTTCATTGGAACACTTTATTGTACACGCATGCCGTTTATGCGCTGCT
TATCCGAGGGTGGCATCACAGGGACCCTACAAGAAGCTGACGGGGCAC
CTCCACTATGAAGTGACATGCCAATGCATGCCCAGGTTGCATGTTCCA
AGTGTCTGGTATCTTTGGCTACGCTGTCTTTTACTAATCGCAAGAAAC
AGTGCTCGTCATGGGTGGTAACTCCTTGGTGCCACCAGAACGCATGTG

GTGCGTTTGTTGTTCCGCTTTGTTTTCTTGGGAGATTTCGCTGCGGC
CTAATGCAGCCAACCCTTTATGGAATTGTGTGCCCTGCTGTCTTGTCA
GG

>TgGoatJpOk10
TTGACATCTGCCTGAACTCGAGGTACTACATCGTCCCCGGCATTGGTG
ATTTCGGTGACCGGTAAGAAATAACGCTTTGAAGGTCGTTGGCTTGTA
TTACCGTCACTACTGCGACGACATACTGGAGAACTCGAGCATCCTTTAT
GGGTTTGTGTTGATGAACGTAGGACTACCAGATATCATTTCTTCATTG
GAACACTTTATTGTACACGCATGCCGTTTATGCGCTGCTTATCCGAGG
GTGGCATCACAGGGACCCTACAAGAAGCTGACGGGGCACCTCCACTAT
GAAGTGACATGCCAATGCATGCCCAGGTTGCATGTTCCAAGTGTCTGG
TATCTTTGGCTACGCTGTCTTTTACTAATCGCAAGAAACAGTGCTCGT
CATGGGTGGTAACTCCTTGGTGCCACCAGAACGCATGTGGTGCGTTT
GTTGTTCCGCTTTGTTTTCTTGGGAGATTTCGCTGCGGCCTAATGCA
GCCAACCCTTTATGGAATTGTGTGCCCTGCTGTCTTGTCAGGTACTTT
GGA

>TgGoatJpOk11
TCGTCCCCGGCATTGGTGATTTCGGTGACCGGTAAGAAATAACGCTTT
GAAGGTCGTTGGCTTGTATTACCGTCACTACTGCGACGACATACTGGA
GAACTCGAGCATCCTTTATGGGTTTGTGTTGATGAACGTAGGACTACC
AGATATCATTTCTTCATTGGAACACTTTATTGTACACGCATGCCGTTTA
TGCGCTGCTTATCCGAGGGTGGCATCACAGGGACCCTACAAGAAGCTG
ACGGGGCACCTCCACTATGAAGTGACATGCCAATGCATGCCCAGGTTG
CATGTTCCAAGTGTCTGGTATCTTTGGCTACGCTGTCTTTTACTAATC
GCAAGAAACAGTGCTCGTCATGGGTGGTAACTCCTTGGTGCCACCAGA
ACGCATGTGGTGCGTTTGTTGTTCCGCTTTGTTTTCTTGGGAGATTT
CGCTGCGGCCTAATGCAGCCAACCCTTTATGGAATTGTGTGCCCTGCT
GTCTTGTCAGGTACTTTGGAACCATGTAGG
