# Supplementary material for: Molecular and biological analysis revealed genetic diversity and high virulence strain of Toxoplasma gondii in Japan
Source: PLoS One. 2020 Feb 3;15(2):e0227749. doi: 10.1371/journal.pone.0227749 (PMC6996823; doi:10.1371/journal.pone.0227749)
Supplement: S2 File — It is noted that bases in the coding sequences are shown in bold letters. (PDF) [file pone.0227749.s005.pdf]

TgCatJpOk3

>ROP5 (consensus sequence)

**ATGGCGACGAAGCTCGCTAGACTAGCCACGTGGCTTGTCTTGGTAGGTT
GCCTGTTGTGGCGGGCGGGGGCAGTTCAGCTCTCTCCGCCAAACTCCA
GGACGAATGATCTGGCTTCGGGAACCCCGCATGTGGCTCGTGGGGACA
CTGAGGCGCAGTCAGGAACTGGAGACGATTCAGATTTCCCGCAGGGCG
TGGTCGAAGAGGTGGCAGATATGAGCGGCGGCAGAGTTCCCCGAGTGC
CAGCATCGTCTACCACCACATCTGCGTCCGAGGGGATTTTCAGAAGATT
AGTTCGCAGACTTCGTCGGGGAAGAGGAACCGCAGATGGCGCAGGAGT
TGCTGACGAAACCCATCAGGGGCCGCGCCCGCCACTTCGGAAGAGACT
TGCTCAGCACTTCCGTAGGCTGAGGGGCTTCTTCGGACGCCTTACGCC
GAGGTGGCTCTCCGGTCTCGGCCGCCGGGCGCAAAGATGGTGGAGAG
GGAGACAGAGACCGCTGCTGGACCCTTCGTTTCATGGGTTGGAAGCTG
GAGATTCGTTCATGCGCGACCTGCTGAAACGTGAAGAAGAGCTGATTG
GATACTGTCGCGAAGAAGCGTTGGAAGAACCTGCAGCGATGGTTGAGG
CTGTCATGGCAACTGTATGGCCGCAAAATGCTGAAACAACCGTGGATTC
ACTTTTGAGTCAGGGAGAGCGGAAGTTGAAATTGGTGGAGCCTCTTCG
AGTCGGTGACCGATCTGTCGTATTTTTAGTAAGGGATGTAGAGCGCCTG
GAGGATTTCGCTCTGAAGGTCTTCACTATGGGTGCCGAGAATTCCCGAT
CAGAGCTGGAGCGGTTGCATGAAGCGACTTTTGCGGCAGCGAGGTTGC
TTGGGGAGAGTCCAGAGGAGGCACGGGACAGACGCAGGCTTTTACTTC
CCTCCGATGCTGTGGCAGTTCAGTCTCAGCCCCCTTTCGCTCAGCTGAG
TCCAGGACAGAGCGACTATGCAGTCGCGAACTATTTCTTTCTCATGCCC
GCTGCGTCGGTGGATCTTGAATTGCTCTTTAGGACATTGGACTTCGTGT
ATGTATTCAGGGGGGAAGAAGGTATTTTAGCGCGTCACTTACTAACGGC
ACAGCTGATCCGTCTGGCAGCCAACCTGCAGAGCAAAGGACTTGTGCA
TGGACGCTTCACACCTGAAAACCTTTTCATTATGCCCGATGGCCGCCTG
ATGATGGGGGATGTATCCACGTTGAGGAAGGTCGGAACCCGAGGACCG
GCATCAAGCGTCCCGGTTACCTATGCGCCTCGGGAGTTCTTGAATGCAA
ACACGGCAACATTTACACACGCGCTCAATGCGTGGCAACTGGGTCTTAG
CATATACCGGGTTTGGTGCCTAGTCTTGCCTTTCGGACTCGTGACACCT
GGGATCAAACGGACATGGAAAAGGCCAAGTCTACGAGTTCCAGGGACT
GACAGTCTGCTATTCGACTCATGTATACCTGTGCCTGACTTCGTGCAGA
CACTTACTAGACGGTTCCTCAACTTCGATAGGCGGCGACGCCTGCTTCC
CTTGGAGGCCATGGAGACGCCAGAGTTCCTCCAGCTCCAAAACGAAATA**

**TCGAGCAGCCTATCAACAGGACAACCTACTGCTGCGCCCTCAGTCGCTT
GA**

>ROP16

**ATGAAAGTGACCACGAAAGGGCTTGCTTTTGCTCTTGCACTGTTGTTTT
GTACACGCTGCGCAACTGCACGATACATGTCGTTTGAGGAAGCGCAAAA
AGCAAGTGAAGCAGCGAAGCGCCAGATTGCCACACTCCCCTCTCCAGA
TTCTACTCTTTCGAATCCAGGTAGCAAGCATAGAAACCGGGGAGGGTCT
CCTGCGGCAGGGCAACCTTCTCAATCCACACTACAACCTGAACAAGCGG
CGGCTGAAGTAGGTCTCGGTGCTGGTGGCTCGACTCAGGGGCAGGGAC
GCACCGGTGGCAGCGCGGGTGCTAGAGAGGAGCGGAGGAGTCCTTCC
CCCCAATCTGCTTATCCGGCGACTAGCTCAGCCTCGCTAAGGGGCTACC
AAACCCAGCTTTCACCCTCGCATCTTCCACCACGCAGCAGCGGACCGG
GAGGATGGTTTCCAACAGAGTCAATATTTACGCCATGGAGTTCTCCGCC
GCAACCATTGACACAACGAAAGCCATCTCTATCTGGGGTGGTCGTTACC
GAATTTCAAGAGCCACAAGAACAGTATGGCGCAGCGAGCAGTCTTGCG
TCCTCGCCAAAGCGATACGTCAGTGGCGCAAGCTCGAGTGCATTGTCAG
GAAAGGCGGTGCCAACGCCTGCGTCGCTTGGTCAAGAAAATCCTCTTTT
CCCTGTTCAGAGCGCTACATTGGATTCAGGAATACAGTCTCCGGCACAA
GAGCGTCGGGGATCCCCTCAAAGACAGATTGCGATGTCGACCGAAAAT
CCAGCGGATAGCGGCGCCTCGCAGCTTGCCTCCAGTGTTTCTAGTTATG
TAGCAGTACAAACTCCTCATGTGAAACGTTCAGAACGCATCCGGCGCGT
TCGACTTTCAGAAGAGGGTCTGGAAGAAGTTCAGCAGCTGAAAGCAGC
TGCCGCACAGCTTCTCGTAGCGGTTCCGGACTATGAGGCAATGCGGGCT
GTTCTGCAAGAGGCGGTCCTCTCAGAACAGAGGGTTGCTACCCGTAAG
CGGAAGAGAAAGCAACCTCCAGGAGCGGTGGAGTCAGCTGTTGACGAA
GTGTTCCCTCCAAATGAGCGTGTCATGATGATAAATGCCAACGGAGTGC
CGATCGCTCTATACAATCGTGGGCACCTCGGCAGTGGACATTTCGGGGC
TGTCATCAAGGCCAGCTTAGACGATGGGACGTTGTATGCAGCGAAGGTG
CCGTACAGCCAGATCGTCCCGAATGCTGATGCCACGTCAGCAGAACTGG
AGGCGGAAATTTCCTCAGCTAGGGCGGAGTTGGTAAAGACAATTCGACA
GGAGTTGGATGTTCGGGATAAGCTGGTGGCTAAAGGGCTCACACTTACA
GAGACTGCGGAGCAATACGGTCTACCATTGTGCCAAATGACTTTAACGC
TTCCTGAGAACAAAGCAACCGTGGTACGTCGAGGTTCTCGACTCGTTGT
CGTGTCTAAAGAAGTCATGCTGCTGCCATTAATTGATGGCTCCCCATCG
AACAGTCTAGTCCAGTCGCAACCACCATTTCTCTTCCAGCGAGCTGTGG**

CAAGGGAAGCAATTATTGCATTGGCCAAGCTTCACGAACTTGGATTCGC
GCATGGAGATGTTAAATTGAACAACATGATGATCGATGTCCACGGCTTT
GGGCATATGCTTGACATGGGCAGTGTGCGGCCTGTTGACAGCTGTGTAA
GCGAGGAAGATAAATATTACCTGCGTTTGTGGGCTCCTGAACTTGCGAA
ATCACAGCACACGTCGCAGCAGACATGTCTGAAGCGTGGCGCTCTCGAT
GTGTGGGCCTTAGGGTTGGCAATCTTCGAGTTCGTCTGCTTCAACCGAC
TTCCTTACAGCCTTTCGAATCTGCCGAGTTCACTCTGGTCGAGAGTTGA
ACACCTTTCGCGCCTTCGCCTCTCAGATTTCTCTGCCAAGGATTGTAAC
GAATCTGATCCAGCAGTGATGGGAATTGTTGCTCAATTTCTAAATCCAAA
TCCTGAAGAGCGCCCTGAACTCCCGAAATTCGTCAGCAGTTACACCTTC
TTTCGGCAAGCCCCTGGAGTTACTTCTCATCTCACTAGGATTCCAACTAC
CGAACTTTCTTCACATCGGATGTAG

>ROP18
ATTCGGGGCAAGAAAGAAGGAAGAAAATAAAGACGAGCGAGCACGCGGAC
TCCTCGGTTCCCTCGTTCGAGCTAGCGACTGCGGGAGAACCACATCTTTGGC
CCGAGATTCGAAACGCGGAAGTAACTCGAGTCGATGCCTCGCTAGATGGCGA
CCAGAAAAGAAAACGAGTGAACTCTTGAAAGCAGCACCCTGAGGAGACAG
CCAGGAAAGAAGGGACAGGAAGAAACTCGAGAGAAGCGGCAGGAGACTGT
CACAGCTCGTCGACCACACAGCTAAACTGCCCGCCCTCTTTCATTAAGTCCC
GTGTTTGAACGACGTCCGGGAGTGCCTTTTTCTTTTCGTTTCCCGACGTAGCA
GCGAACCTAGCTGAATATTCCAGTTTCGCTTTGAGTTCATAACTCCTTCGTTC
TCTCTCTTCCGCCCGTCAGTTCCCTTCCCTGGTGTCTTCGTGACATTTTATTAA
GTTCTATCGCGCCACTCGGACTTTGTTCCTGGCACCCTTTTGTAGCTAGGACT
CCTTGACGAGTCAGTCAGATTGACGAGGTCGGCTAGCTAGGACTCCTTGACG
CGTCAGTCAGATTGACGAGGCCGGCTAGCTAGGACTCCTTGACGCGTCAGTC
AGATTGACGAGGCCGGCTAGCCACGCTATGCACCTCTTGCATACAATTGTTGT
AGACAGCATGGTGGTGTGCGAGACACTGCAGTCAAATGCCTCACCCAACGC
CGCGTCTCATTCTTCCAAAAACCTGTCCGTGTCTTCGACAGATTGATACAGCC
GTTGACAAAGCAATACCATATTTTACAGTTTTGTACTCACCCCAGTCCAGTTT
GTGTGAAAGTTGTG**ATGTTTTCGGTACAGCGGCCACCTCTTACGCGTACC**
GTCGTCCGAATGGGTTTAGCGACTCTTCTCCCGAAGACAGCCTGTCTTG
CGGTGTTAAATGTAGCGCTTGTCTTCCTGCTCTTCCAAGTCCAGGATGG
GACCGGAATCACACTTGATCCTTCAAAACTCGACTCCAAACCGACAAGT
TTGGATTCGCAACAGCACGTTGCTGACAAGCGGTGGCCTGCTACAGTT
GGCCACTACAAATATTTAGCAGGAGCGACAGAAAGCACTCGAGACGTTT

**CATTGCTGGAGGAAAGGGCTCAACACCGGGTAAATGCGCAAGAAACAA
ACCAACGGCGCACGATTTTTCAGAGGCTTCTGAATCTCTTGAGACGGAG
AGAAAGAGATGGTGAAGTCTCGGGTTCCGCAGCTGATAGCTCCTCGAG
ACCCCGTCTGTCCGTACGACAGAGGCTTGCTCAACTTTGGCGTAAAGCG
AAATCGTTCTTCACACGCGGAATCCCGAGGTACTTTTCTCAAGGGCGTA
ACCGACTGCGAAGTTTGCGGGCACAAAGACGGCGATCTGAATTGTTTTT
TGAGAAGGCGGATTCTGGATGCGTCATCGGCAAACGCATCCTGGCGCA
CATGCAAGAACAAATCGGGCAGCCTCAAGCGCTAGGAAATAGTGAACGA
CTGGATAGAATTCTGACTGTCGCTGCCTGGCCTCCGGACGTTCCAGAAA
GATTTGTTTCTGTGACTACCGGTGAAACCCGGACGCTGGTGAGAGGTG
CACCCCTTGGCTCTGGTGGATTCGCCACTGTATATGAGGCTACAGACGT
GGAGACGAATGAAGAGTTGGCTGTTAAGGTTTTCATGTCAGAAAAGGA
GCCCACCGATGAGACTATGCGTGACTTGCAGAGGGAGTCGTTCTGCTAC
AGGAACTTTAGTCTAGCCAAGACGGCGAAGGATGCCCAGGAACGCTGT
AGATTCATGGTTCCTAGTGATGTTGTGATGTTAGAGGGACAGCCAGCAT
CCACAGAGGTCGTGATTGGTTTGACGACTCGGTGGGTACCAAACTATTT
TCTTCTCATGATGCGGGCAGAAACGGACATGAGCAAAGTCATTTCATGG
GTATTTGGAGATGCGTCTGTCAATAACAGTGAATTAGGCCTGGTCGTTC
GAATGTACCTATCCAGTCAGGCAATCAGACTAGTGGCCAATGTTCAAGC
TCAGGGAATTGTGCATACGGATATCAAACCGGCGAATTTCCTCCTCTTG
AAAGACGGTCGCCTGTTTCTCGGCGACTTCGGAACGTATAGAATCAATA
ATTCGGTTGGACCCGCGATAGGTACTCCCGGTTACGAGCCTCCGGAGCG
ACCGTTTCAGACTACAGGCATCACCTATACATTCACCACTGACGCGTGG
CAACTCGGTATAACTTTGTACTGCATCTGGTGCAAGGAACGTCCAACTC
CGGCCGACGGCATCTGGGACTACTTACACTTCGCAGATTGTCCTTCCAC
GCCTGAGCTGGTTCAAGACCTCATCCGAAACCTCTTGAATCGAGAGCCT
CAGAAACGGATGCTCCCGCTACAAGCCTTGGAGACCGCAGCGTTTAAC
GAGATGGATTCAGTAGTAAAACGCGCCGCGCAAAACTTCGAACAGCAG
GAACATCTCCACACAGAATAA**

TgCatJpOk4

>ROP5 (consensus sequence)

**ATGGCGACGAAGCTCGCTAGACTAGCCACGTGGCTTGTCTTGGTAGGTT
GCCTGTTGTGGCGGGCGGGGGCAGTTCAGCTCTCTCCGCCAAACTCCA
GGACGAATGATCTGGCTTCGGGAACCCCGCATGTGGCTCGTGGGGACA
CTGAGGCGCAGTCAGGAACTGGAGACGATTCAGATTTTCCGCAGGCCG
TGGCCGAAGAGGTGGCAGATATGAGCGGCGGCAGAGTTCCCCGAGTGC
CAGCATCGTCTACCACCACATCTGCGTCCGAGGGGATTTTCAGAAGATT
AGTTCGCAGACTTCGTCGGGGAAGAGGAACCGCAGATGGCGCAGGAGT
TGCTGACGAAACCCATCAGGAGCCGCGCCCGCCACTTCGGAAGAGACT
TGCTCAGCACTTCCGTAGGCTGAGGGGCTTCTTCGGACGCCTTACGCC
GAGGTGGCTCTCCGGTCTCGGCCGCCGGGCGCAAAGATGGTGGAGAG
GGAGACAGAGACCGCTGCTGGACCCTTCGTTTCATGGGTTGGAAGCTG
GAGATTCGTTCATGCGCGACCTGCTGAAACGTGAAGAAGAGCTGATTG
GATACTGTCGCGAAGAAGCGTTGAAAGAACCTGCAGCGATGGTTGAGG
CTGTCACGGCAACTGTATGGCCGCAAAATGCTGAAACAACCGTGGATTC
GCTTTTGAGTCAGGGAGAGCGGAAGTTGAAATTGGTGGAGCCTCTTCG
AGTCGGTGACCGATCTGTCGTATTTTTAGTAAGGGATGTAGAGCGCCTG
GAGGATTTCGCTCTGAAGGTCTTCACTATGGGTGCCGAGAATTCCCGAT
CAGAGCTGGAGCGGTTGCATGAAGCGACTTTTGCGGCAGCGAGGTTGC
TTGGGGAGAGTCCAGAGGAGGCACGGGACAGACGCAGGCTTTTACTTC
CCTCCGATGCTGTGGCAGTTCAGTCTCAGCCCCCTTTCGCTCAGCTGAG
TCCAGGACAGAGCGACTATGCAGTCGCGAACTACTTGCTTCTCATGCCC
GCTGCGTCGGTGGATCTTGAATTGCTCTTTAGCACATTGGACTTCGTGT
ATGTATTCAGGGGGGCAGAAGATTTTTTAGCGCTTCACATACTAACGGC
ACAGCTGATCCGTCTGGCAGCCAACCTGCAGAGCAAAGGACTTGTGCA
TGGACATTTCACACCGGATAACCTTTTCATTATGCCCGATGGCCGCCTGA
TGCTGGGGGATGTATCCGTGTTGAGGAAGGTCGGAACCCGAGGACCGG
CATCAAGCGTCCCGGTTACCTATGCGCCTCGCGAGTTCTTGAATGCAAG
CACGGCAACATTTACACACGCGCTGGATGCGTGGCAACTGGGTCTTAGC
ATATACCGGGTTTGGTGCCTATTCTTGCCTTTCGGACTCGTGACACCTG
GGATCAAAGGGTCATGGAAAAGACCAAGTCTACGAGTTCCAGGGACTG
ACAGTCTGGCATTCGGCTCATGTACACCTCTGCCTGACTTCGTGCAGAC
ACTTATTGGACGGTTCCTCAACTTCGATAGGCGTCGACGCCTGCTTCCC
TTGGAGGCCATGGAGACGCCAGAGTTCCTCCAGCTCCAAAACGAAATAT
CGAGCAGCCTATCAACAGGACAACCCATTGCTGCGCCCTCAGTCGCTTG**

A

>ROP16

ATGAAAGTGACCACGAAAGGGCTTGCTTTTGCTCTTGCACTGTTGTTTT
GTACACGCTGCGCAACTGCACGATACATGTCGTTTGAGGAAGCGCAAAA
AGCAAGTGAAGCAGCGAAGCGCCAGATTGCCACACTCCCCTCTCCAGA
TTCTACTCTTTCGAATCCAGGTAGCAAGCATAGAAACCGGGGAGGGTCT
CCTGCGGCAGGGCAACCTTCTCAATCCACACTACAACCTGAACAAGCGG
CGGCTGAAGTAGGTCTCGGTGCTGGTGGCTCGACTCAGGGGCAGGGAC
GCACCGGTGGCAGCGCGGGTGCTAGAGAGGAGCGGAGGAGTCCTTCC
CCCCAATCTGCTTATCCGGCGACTAGCTCAGCCTCGCTAAGGGGCTACC
AAACCCAGCTTTCACCCTCGCATCTTCCACCACGCAGCAGCGGACCGG
GAGGATGGTTTCCAACAGAGTCAATATTTACGCCATGGAGTTCTCCGCC
GCAACCATTGACACAACGAAAGCCATCTCTATCTGGGGTGGTCGTTACC
GAATTTCAAGAGCCACAAGAACAGTATGGCGCAGCGAGCAGTCTTGCG
TCCTCGCCAAAGCGATACGTCAGTGGCGCAAGCTCGAGTGCATTGTCAG
GAAAGGCGGTGCCAACGCCTGCGTCGCTTGGTCAAGAAAATCCTCTTTT
CCCTGTTCAGAGCGCTACATTGGATTCAGGAATACAGTCTCCGGCACAA
GAGCGTCGGGGATCCCCTCAAAGACAGATTGCGATGTCGACCGAAAAT
CCAGCGGATAGCGGCGCCTCGCAGCTTGCCTCCAGTGTTTCTAGTTATG
TAGCAGTACAAACTCCTCATGTGAAACGTTCAGAACGCATCCGGCGCGT
TCGACTTTCAGAAGAGGGTCTGGAAGAAGTTCAGCAGCTGAAAGCAGC
TGCCGCACAGCTTCTCGTAGCGGTTCCGGACTATGAGGCAATGCGGGCT
GTTCTGCAAGAGGCGGTCCTCTCAGAACAGAGGGTTGCTACCCGTAAG
CGGAAGAGAAAGCAACCTCCAGGAGCGGTGGAGTCAGCTGTTGACGAA
GTGTTCCCTCCAAATGAGCGTGTCATGATGATAAATGCCAACGGAGTGC
CGATCGCTCTATACAATCGTGGGCACCTCGGCAGTGGACATTTCGGGGC
TGTCATCAAGGCCAGCTTAGACGATGGGACGTTGTATGCAGCGAAGGTG
CCGTACAGCCAGATCGTCCCGAATGCTGATGCCACGTCAGCAGAACTGG
AGGCGGAAATTTCCTCAGCTAGGGCGGAGTTGGTAAAGACAATTCGACA
GGAGTTGGATGTTCGGGATAAGCTGGTGGCTAAAGGGCTCACACTTACA
GAGACTGCGGAGCAATACGGTCTACCATTGTGCCAAATGACTTTAACGC
TTCCTGAGAACAAAGCAACCGTGGTACGTCGAGGTTCTCGACTCGTTGT
CGTGTCTAAAGAAGTCATGCTGCTGCCATTAATTGATGGCTCCGCATTG
AACAGTCTAGTCCAGTCGCAACCACCATTTCTCTTCCAGCGAGCTGTGG
CAAGGGAAGCAATTATTGCATTGGCCAAGCTTCACGAACTTGGATTCGC

GCATGGAGATGTTAAATTGAACAACATGATGATCGATGTCCACGGCTTT
GGGCATATGCTTGACATGGGCAGTGTGCGGCGTGTTGACAGCTGTGTAA
GCGAGGAAGATAAATATTACCTGCGTTTGTGGGCTCCTGAACTTGCGAA
ATCACAGCACACGTCGCAGCAGACATGTCTGAAGCGTGGCGCTCTCGAT
GTGTGGGCCTTAGGGTTGGCAATCTTCGAGTTCGTCTGCTTCAACCGAC
TTCCTTACAGCCTTTCGAATCTGCCGAGTTCACTCTGGTCGAGAGTTGA
ACACCTTTCGCGCCTTCGCCTCTCAGATTTCTCTGCCAAGGATTGTAAC
GAATCTGATCCAGCAGTGATGGGAATTGTTGCTCAATTTCTAAATCCAAA
TCCTGAAGAGCGCCCTGAACTCCCGAAATTCGTCAGCAGTTACACCTTC
TTTCGGCAAGCCCCTGGAGTTACTTCTCATCTCACTAGGATTCCAACTAC
CGAACTTTCTTCACATCGGATGTAG

>ROP18
ATTCGGGGCAAGAAAGAAGGAAGAAAATAAAGACGAGCGAGCACGCGGAC
TCCTCGGTTCCCTCGTTCGAGCTAGCGACTGCGGGAGAACCACATCTTTGGC
CCGAGATTCGAAACGCGGAAGTAACTCGAGTCGATGCCTCGCTAGATGGCGA
CCAGAAAAGAAAACGAGTGAACTCTTGAAAGCAGCACCCTGAGGAGACAG
CCAGGAAAGAAGGGACAGGAAGAAACTCGAGAGAAGCGGCAGGAGACTGT
CACAGCTCGTCGACCACACAGCTAAACTGCCCGCCCTCTTTCATTAAGTCCC
GTGTTTGAACGACGTCCGGGAGTGCCTTTTTCTTTTCGTTTCCCGACGTAGCA
GCGAACCTAGCTGAATATTCCAGTTTCGCTTTGAGTTCATAACTCCTTCGTTC
TCTCTCTTCCGCCCGTCAGTTCCCTTCCCTGGTGTCTTCGTGACATTTTATTAA
GTTCTATCGCGCCACTCGGACTTTGTTCCTGGCACCCTTTTGTAGCTAGGACT
CCTTGACGAGTCAGTCAGATTGACGAGGTCGGCTAGCTAGGACTCCTTGACG
CGTCAGTCAGATTGACGAGGCCGGCTAGCTAGGACTCCTTGACGCGTCAGTC
AGATTGACGAGGCCGGCTAGCCACGCTATGCACCTCTTGCATACAATTGTTGT
AGACAGCATGGTGGTGTGCGAGACACTGCAGTCAAATGCCTCACCCAACGC
CGCGTCTCATTCTTCCAAAAACCTGTCCGTGTCTTCGACAGATTGATACAGCC
GTTGACAAAGCAATACCATATTTACAGTTTTGTACTCACCCCAGTCCAGTTT
GTGTGAAAGTTGTG**ATGTTTTCGGTACAGCGGCCACCTCTTACGCGTACC**
GTCGTCCGAATGGGTTTAGCGACTCTTCTCCCGAAGACAGCCTGTCTTG
CGGTGTTAAATGTAGCGCTTGTCTTCCTGCTCTTCCAAGTCCAGGATGG
GACCGGAATCACACTTGATCCTTCAAAACTCGACTCCAAACCGACAAGT
TTGGATTCGCAACAGCACGTTGCTGACAAGCGGTGGCCTGCTACAGTT
GGCCACTACAAATATTTAGCAGGAGCGACAGAAAGCACTCGAGACGTTT
CATTGCTGGAGGAAAGGGCTCAACACCGGGTAAATGCGCAAGAAACAA

ACCAACGGCGCACGATTTTTCAGAGGCTTCTGAATCTCTTGAGACGGAG
AGAAAGAGATGGTGAAGTCTCGGGTTCCGCAGCTGATAGCTCCTCGAG
ACCCCGTCTGTCCGTACGACAGAGGCTTGCTCAACTTTGGCGTAAAGCG
AAATCGTTCTTCACACGCGGAATCCCGAGGTACTTTTCTCAAGGGCGTA
ACCGACTGCGAAGTTTGCGGGCACAAAGACGGCGATCTGAATTGTTTTT
TGAGAAGGCGGATTCTGGATGCGTCATCGGCAAACGCATCCTGGCGCA
CATGCAAGAACAAATCGGGCAGCCTCAAGCGCTAGGAAATAGTGAACGA
CTGGATAGAATTCTGACTGTCGCTGCCTGGCCTCCGGACGTTCCAGAAA
GATTTGTTTCTGTGACTACCGGTGAAACCCGGACGCTGGTGAGAGGTG
CACCCCTTGGCTCTGGTGGATTCGCCACTGTATATGAGGCTACAGACGT
GGAGACGAATGAAGAGTTGGCTGTTAAGGTTTTCATGTCAGAAAAGGA
GCCCACCGATGAGACTATGCGTGACTTGCAGAGGGAGTCGTTCTGCTAC
AGGAACTTTAGTCTAGCCAAGACGGCGAAGGATGCCCAGGAACGCTGT
AGATTCATGGTTCCTAGTGATGTTGTGATGTTAGAGGGACAGCCAGCAT
CCACAGAGGTCGTGATTGGTTTGACGACTCGGTGGGTACCAAACTATTT
TCTTCTCATGATGCGGGCAGAAACGGACATGAGCAAAGTCATTTCATGG
GTATTTGGAGATGCGTCTGTCAATAACAGTGAATTAGGCCTGGTCGTTC
GAATGTACCTATCCAGTCAGGCAATCAGACTAGTGGCCAATGTTCAAGC
TCAGGGAATTGTGCATACGGATATCAAACCGGCGAATTTCCTCCTCTTG
AAAGACGGTCGCCTGTTTCTCGGCGACTTCGGAACGTATAGAATCAATA
ATTCGGTTGGACCCGCGATAGGTACTCCCGGTTACGAGCCTCCGGAGCG
ACCGTTTCAGACTACAGGCATCACCTATACATTCACCACTGACGCGTGG
CAACTCGGTATAACTTTGTACTGCATCTGGTGCAAGGAACGTCCAACTC
CGGCCGACGGCATCTGGGACTACTTACACTTCGCAGATTGTCCTTCCAC
GCCTGAGCTGGTTCAAGACCTCATCCGAAACCTCTTGAATCGAGAGCCT
CAGAAACGGATGCTCCCGCTACAAGCCTTGGAGACCGCAGCGTTTAAC
GAGATGGATTCAGTAGTAAAACGCGCCGCGCAAAACTTCGAACAGCAG
GAACATCTCCACACAGAATAA
