# Supplementary figures and images for: Molecular and biological analysis revealed genetic diversity and high virulence strain of Toxoplasma gondii in Japan
Source: PLoS One. 2020 Feb 3;15(2):e0227749. doi: 10.1371/journal.pone.0227749 (PMC6996823; doi:10.1371/journal.pone.0227749)

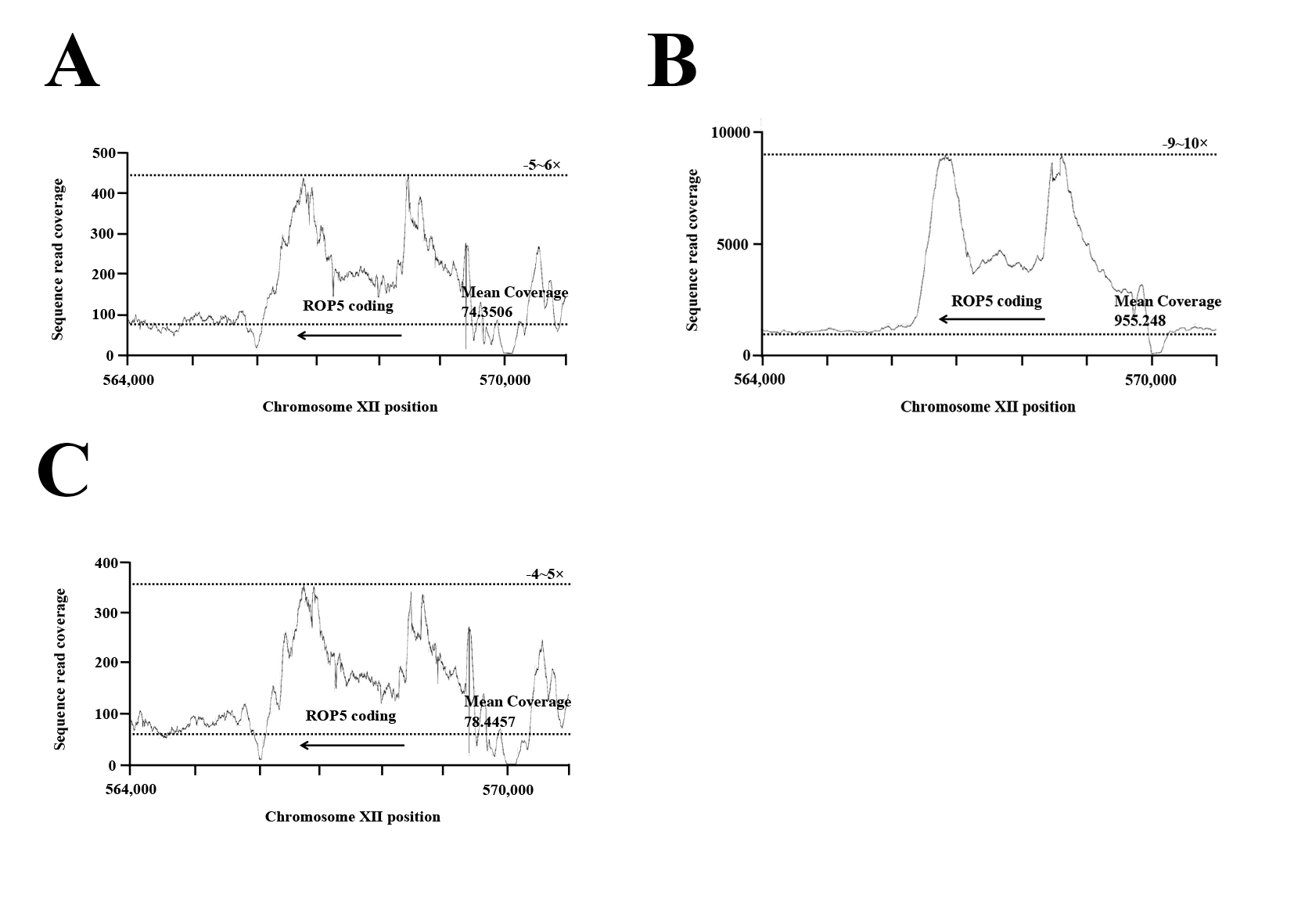

Supplement: S3 File — (A,B,C) Sequence coverages around the ROP5 coding region in GT1 (A), ME49 (B) and VEG (C) are shown. Arrows indicate the coding region of ROP5 on the chromosome XII. (TIF) [file pone.0227749.s006.tif]
